# Supplementary material for: FOXD1 and Gal-3 Form a Positive Regulatory Loop to Regulate Lung Cancer Aggressiveness
Source: Cancers (Basel). 2019 Nov 28;11(12):1897. doi: 10.3390/cancers11121897 (PMC6966623; doi:10.3390/cancers11121897)
Supplement: Supplementary file 1 [file cancers-11-01897-s001.zip › cancers-644902-supplementary/Supplementary/Cancers-644902-Supplement information.docx]

**Supplementary Information**

FOXD1 and Gal-3 Form a Positive Regulatory Loop to Regulate Lung Cancer Aggressiveness

Chien-Hsiu Li ^1,2^, Yu-Chan Chang ^3^, Michael Hsiao ^3,4,^* and Shu-Mei Liang ^1,2,^*

^1^ Graduate Institute of Life Sciences, National Defense Medical Center, Taipei 114, Taiwan

^2^ Agricultural Biotechnology Research Center, Academia Sinica, Taipei, 115, Taiwan

^3^ Genomics Research Center, Academia Sinica, Taipei 115, Taiwan

^4^ Department of Biochemistry, College of Medicine, Kaohsiung Medical University, Kaohsiung, Taiwan

* Correspondence: smyang@gate.sinica.edu.tw (S.M.L.); mhsiao@gate.sinica.edu.tw (M.H.); Tel.: +886-2-2787-2082 (S.M.L.); Tel.: +886-2-2787-1243 (M.H.)

**Supplementary Materials**

**Key Resources Table**

| REAGENT or RESOURCE | sOURCE | IDENTIFIER |
| --- | --- | --- |
| **Antibodies** |  |  |
| Rabbit polyclonal anti-FOXD1 (western blot 1:500) | GeneTex | Cat #GTX100271 |
| Mouse monoclonal anti-Gal-3 (western blot 1:1000) | Cell signaling | Cat #87985 |
| Mouse monoclonal anti-GAPDH (western blot 1:3000) | Santa cruz | Cat #32233 |
| Rabbit polyclonal anti-PCNA (western blot 1:2000) | Cell signaling | Cat #13110 |
| Rabbit polyclonal anti-ITGB1 (western blot 1:3000) | Cell signaling | Cat #4706 |
| Rabbit polyclonal anti-P-FAK (western blot 1:1000) | Cell signaling | Cat #8556 |
| Rabbit monoclonal anti-FKA (western blot 1:2000) | Cell signaling | Cat #13009 |
| Rabbit polyclonal anti-P-PI3K (western blot 1:1000) | Invitrogen | Cat #PA5-17387 |
| Rabbit polyclonal anti-PI3K (western blot 1:2000) | Cell signaling | Cat #4292 |
| Rabbit polyclonal anti-P-AKT (western blot 1:1000) | Cell signaling | Cat #4060 |
| Rabbit monoclonal anti-AKT (western blot 1:2000) | Cell signaling | Cat #4685 |
| Rabbit polyclonal anti-P-PHB1 (western blot 1:1000) | Generated by Abnova | Customized |
| Rabbit polyclonal anti-PHB (western blot 1:2000) | Abcam | Cat #ab224653 |
| Rabbit polyclonal anti-Ras (western blot 1:2000) | GeneTex | Cat #GTX132480 |
| Rabbit polyclonal anti-P-Raf-1 (western blot 1:1000) | Cell signaling | Cat #9427 |
| Rabbit polyclonal anti-Raf-1 (western blot 1:2000) | Cell signaling | Cat #9422 |
| Rabbit polyclonal anti-P-MEK-1 (western blot 1:500) | Santa Cruz | Cat #SC-7995 |
| Rabbit polyclonal anti-MEK-1 (western blot 1:2000) | Cell signaling | Cat #9124 |
| Rabbit polyclonal anti-P-ERK (western blot 1:1000) | Millopore | Cat #05-797R |
| Rabbit polyclonal anti-ERK (western blot 1:2000) | Cell signaling | Cat #9102 |
| Rabbit polyclonal anti-Snail (western blot 1:500) | Santa Cruz | Cat #sc-28199 |
| Rabbit polyclonal anti-Slug (western blot 1:500) | Santa Cruz | Cat #sc-15391 |
| Rabbit polyclonal anti-Zeb1 (western blot 1:500) | Santa Cruz | Cat #sc-25388 |
| Rabbit polyclonal anti-MMP2 (western blot 1:1000) | GeneTex | Cat #GTX104577 |
| Rabbit polyclonal anti-MMP9 (western blot 1:1000) | GeneTex | Cat #GTX100458 |
| Rabbit polyclonal anti-FOXD1 (IHC 1:50) | Aviva Systems Biology | Cat #OAAB10686 |
| Mouse monoclonal anti-Gal-3 (IHC 1:400) | R&D | Cat #MAB11541 |
| Nornal Mouse IgG | Santa Cruz | Cat #sc-2025 |
| Nornal Rabbit IgG | Santa Cruz | Cat #sc-2027 |
| EasyBlot anti-Rabbit IgG (HRP) | GeneTex | Cat #GTX221666-01 |
| EasyBlot anti-Mouse IgG (HRP) | GeneTex | Cat #GTX221667-01 |
| Rabbit IgG antibody (HRP) | GeneTex | Cat #GTX213110-01 |
| Mouse IgG antibody (HRP) | GeneTex | Cat #GTX213111-01 |
| [Alexa Fluor 488 Polyclonal Antibody](https://www.thermofisher.com/antibody/product/A11094) | Invitrogen | Cat #A-11094 |
| Rodamine Red^TM^-X, Goat anti-Mouse IgG (H+L) | Invitrogen | Cat #R-6393 |
| **Critical Commercial Assays, Reagents** |  |  |
| Nuclear/Cytosol Fractionation Kit | Biovision | Cat #K266 |
| Firefly Luciferase Assay kit | Promega | Cat #E1501 |
| EZ-Magna ChIP^TM^ A/G Chromatin Immunoprecipitation Kit | Millipore | Cat #17-10086 |
| Cell Counting Kit-8 (WST-8) | Dojindo | Cat #CK04-05 |
| TOOLSQuant II Fast RT Kit | Tools | Cat #KRT-BA06-2 |
| Luminata Forta western HRP substrate | Millipore | Cat #WBLUF0500 |
| Membrane Protein Extraction Kit | Biovision | Cat #K268 |
| Protein G Mag Sepharose® Xtra | GE | Cat #28967070 |
| **Biological Samples** |  |  |
| Lung cancer tissue array | Biomax | Cat #BC041115d |
| **Chemicals, si-RNA and Recombinant Proteins** |  |  |
| PD98059 | selleckchem | Cat #S1177 |
| si-CTRL | Santa Cruz | Cat #sc-37007 |
| si-FOXD1 | Santa Cruz | Cat #sc-60649 |
| si-Gal3 | Santa Cruz | Cat #sc-155994 |
| si-ERK1 | Santa Cruz | Cat #sc-29307 |
| si-ERK2 | Santa Cruz | Cat #sc-35335 |
| si-ITGB1 | Santa Cruz | Cat #sc-35674 |
| DMSO | Sigma | Cat #SI-D2650-100ML |
| Crystal violet solution | Sigma | Cat #SI-HT90132-1L |
| Human recombinant Gal-3 | R&D | Cat #8259-GA-050 |
| **Oligonucleotides** |  |  |
| Forward primer: 5’-ATGGGGAAGGTGAAGGTCGG-3’ | Genomic company, Taiwan | GAPDH |
| Reverse primer: 5’-CTGTGTGTCCATGGGAGATG-3’ | Genomic company, Taiwan | GAPDH |
| Forward primer: 5’-ACAACTAAGCCTTTTTGAGG-3’ | Genomic company, Taiwan | FOXD1 |
| Reverse primer: 5’-AAAAGTACACCAGACAAGTG-3’ | Genomic company, Taiwan | FOXD1 |
| Forward primer: 5’-GTGCCTCGCATGCTGATAAC-3’ | Genomic company, Taiwan | Gal-3 |
| Reverse primer: 5’-ACTGTCTTTCTTCCCTTCCCC-3’ | Genomic company, Taiwan | Gal-3 |
| Forward primer: 5’-TGGAGTCAACCCAGCCTATC-3’ | Genomic company, Taiwan | ETS-1 |
| Reverse primer: 5’-TCTGCAAGGTGTCTGTCTGG-3’ | Genomic company, Taiwan | ETS-1 |
| Forward primer: 5’-CGCTTGACATCACTGAAGGA-3’ | Genomic company, Taiwan | ZEB2 |
| Reverse primer: 5’-CTTGCCACACTCTGTGCATT-3’ | Genomic company, Taiwan | ZEB2 |
| Forward primer: 5’-TGCACTGAGTGTGGAAAAGC-3’ | Genomic company, Taiwan | ZEB1 |
| Reverse primer: 5’-TGGTGATGCTGAAAGAGACG-3’ | Genomic company, Taiwan | ZEB1 |
| Forward primer: 5’-GGTCTCTGAGGGTCAAGCAG-3’ | Genomic company, Taiwan | MMP1 |
| Reverse primer: 5’-AGTTCATGAGCTGCAACACG-3’ | Genomic company, Taiwan | MMP1 |
| Forward primer: 5’-TGCTTCTGGCAACTTCATTG-3’ | Genomic company, Taiwan | PEG10 |
| Reverse primer: 5’-TCAAATGACAGCACCTCTCG-3’ | Genomic company, Taiwan | PEG10 |
| Forward primer: 5’-AGCAACCAAGAGGCAAGAAA-3’ | Genomic company, Taiwan | CD44 |
| Reverse primer: 5’-GTGTGGTTGAAATGGTGCTG-3’ | Genomic company, Taiwan | CD44 |
| Forward primer: 5’-ATCAGCCAGTGAGGGTCAAC-3’ | Genomic company, Taiwan | FLI1 |
| Reverse primer: 5’-GGCCATTCTTCTCGTCCATA-3’ | Genomic company, Taiwan | FLI1 |
| Forward primer: 5’-GTCCGCAGTCTTACGAGGAG-3’ | Genomic company, Taiwan | TWIST1 |
| Reverse primer: 5’-TGGAGGACCTGGTAGAGGAA-3’ | Genomic company, Taiwan | TWIST1 |
| EpiTect ChIP qPCR Primer Assay For Human LGALS3, NR_003225.1 (-)10Kb | QIAGEN | Cat #GPH1003937(-)10A |
| **Software and Algorithms** |  |  |
| GraphPad Prism | GraphPad Software | Version 5.0.1 |
| Adobe Photoshop CS2 | Adobe | Version 9.0 |
| Image Scope | Aperio | Version 9.0 |
| Gropu-based Prediction System | The CUCKOO WORKGROUP | Version 5.0 |

**Supplementary Figures and Figure Legends**

**
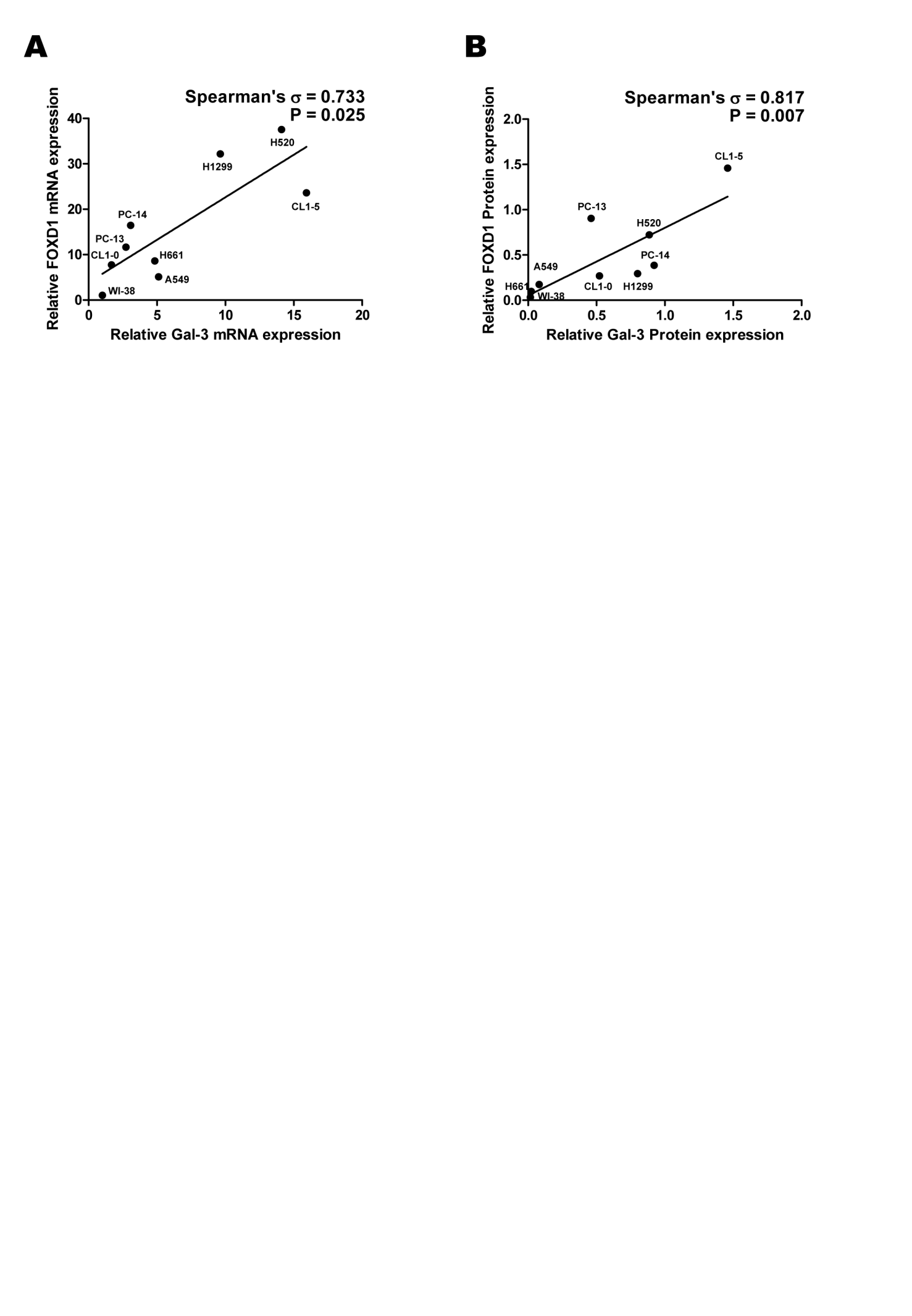
**

**Figure S1.** Relative mRNA and protein expression of FOXD1 and Gal-3 in lung cancer cell lines. (A) *FOXD1* and *Gal-3* relative mRNA expression statistical correlation. The relative expression of the genes was normalized to *GAPDH* as an internal control. (B) FOXD1 and Gal-3 relative protein expression statistical correlation. The relative expression of the genes was normalized to GAPDH as an internal control.

**
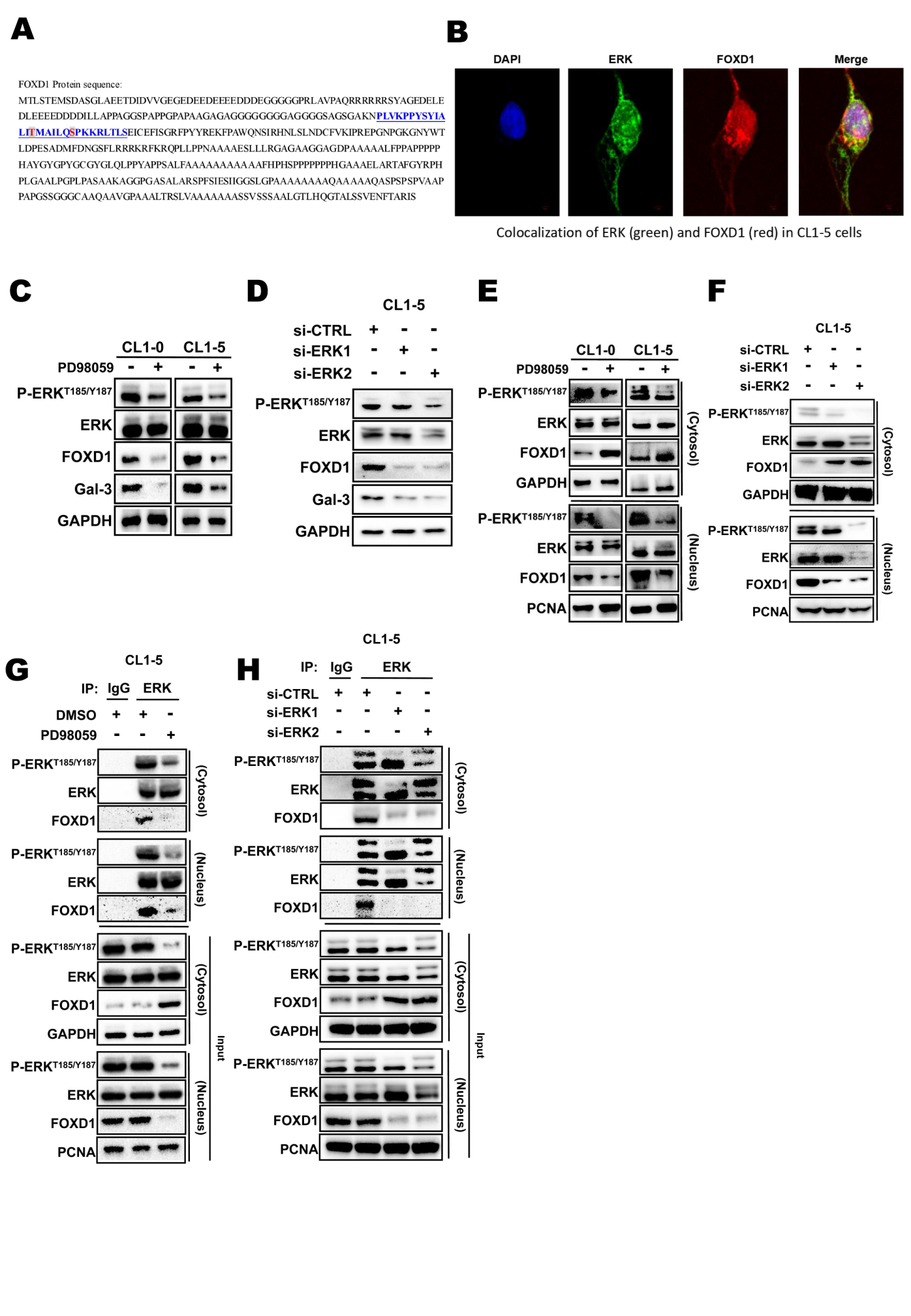
**

**Figure S2.** ERK1 and ERK2 bind FOXD1 and translocate into the nucleus. (A) Group-based prediction system (GPS) prediction identified T135 (red) and S141 (red) on FOXD1 nuclear localization signals (deep blue) may association with ERK. (B) Immunofluorescence staining shows the colocalization of ERK (green) and FOXD1 (red) in the cytosol and nucleus (blue) of CL1-5 cells. CL1-0 and CL1-5 cells were treated with 10 μM or 29 μM PD98059 for 24 h or transfected with specific siRNA for 72 h. (C, D) The whole cell lysates were collected and analyzed by immunoblotting, (E, F) cytosol or nuclear fractions were collected and analyzed by immunoblotting. (G) Cells were treated with PD98059 for 24 h or (H) transfected with ERK1 or ERK2 siRNA for 72 h, and immunoprecipitation results were analyzed by immunoblotting. Input, the whole cell lysates.

**
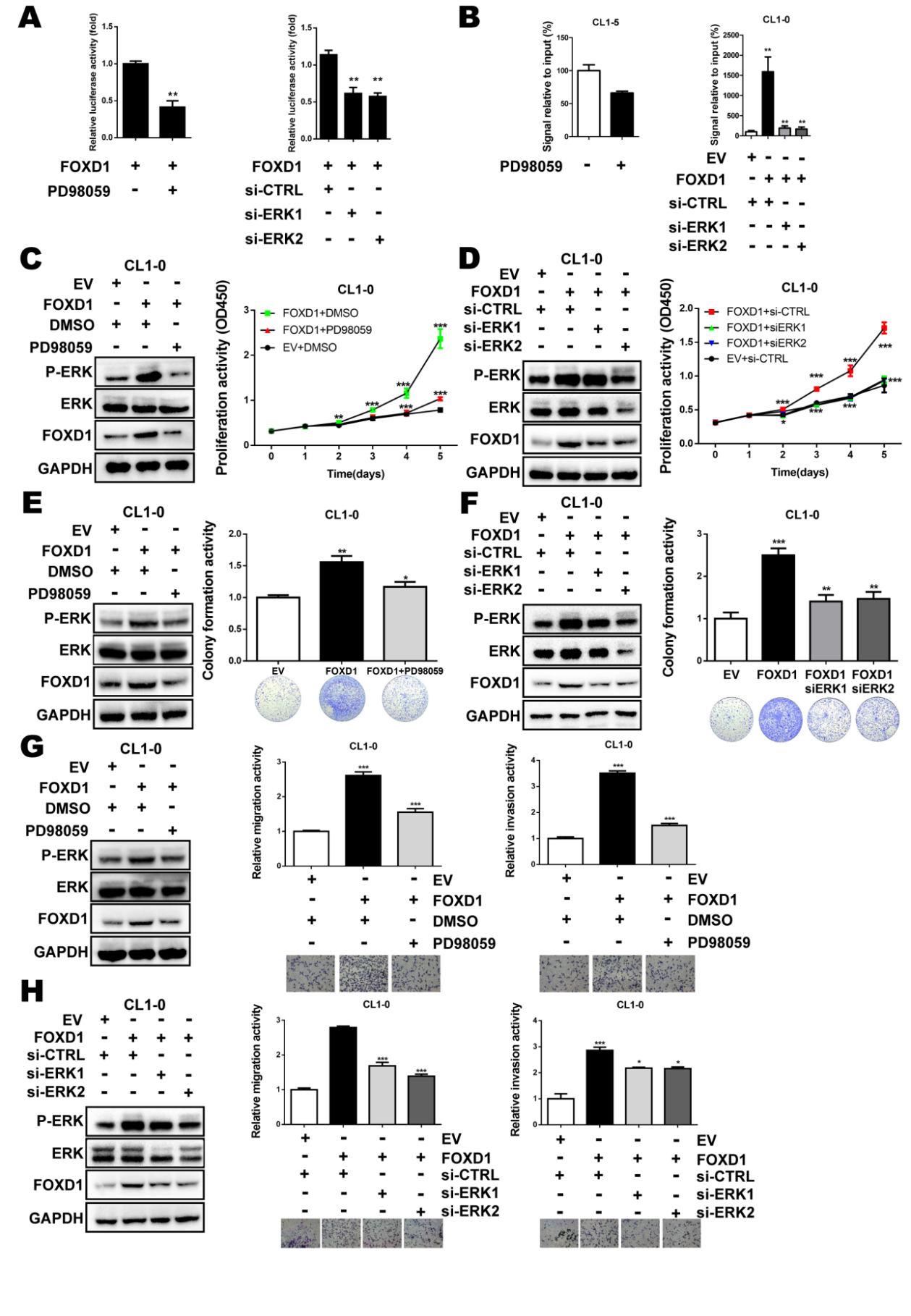
**

**Figure S3.** Interaction of ERK and FOXD1 is required for *Gal-3* transactivation. (A) CL1-0 cells were transfected with the *Gal-3* promoter reporter (pGL3-*Gal-3*) and *FOXD1* vectors for 48 h (left); CL1-0 cells were transfected with *Gal-3* promoter reporter (pGL3-*Gal-3*) and *ERK1*-siRNA or *ERK2*-siRNA for 72h and evaluated by luciferase activity (right). (B) ChIP assay in CL1-5 cells treated with PD98058 29 µM for 24 h (left); or CL1-0 cells were transfected with *FOXD1* vector and *ERK1*, *ERK2* siRNA (right) for 72h. FOXD1 binding to *Gal-3* promoter was measured by qPCR. (C-H) CL1-0 cells were treated or transfected with DMSO, PD98059, scramble, *FOXD1*, *ERK1* or *ERK2* siRNA. (C, D) Proliferation ability was analyzed by CCK-8 assay. (E, F) Representative images of colony-forming assay (G, H) Representative images of migration and invasion assay.

**
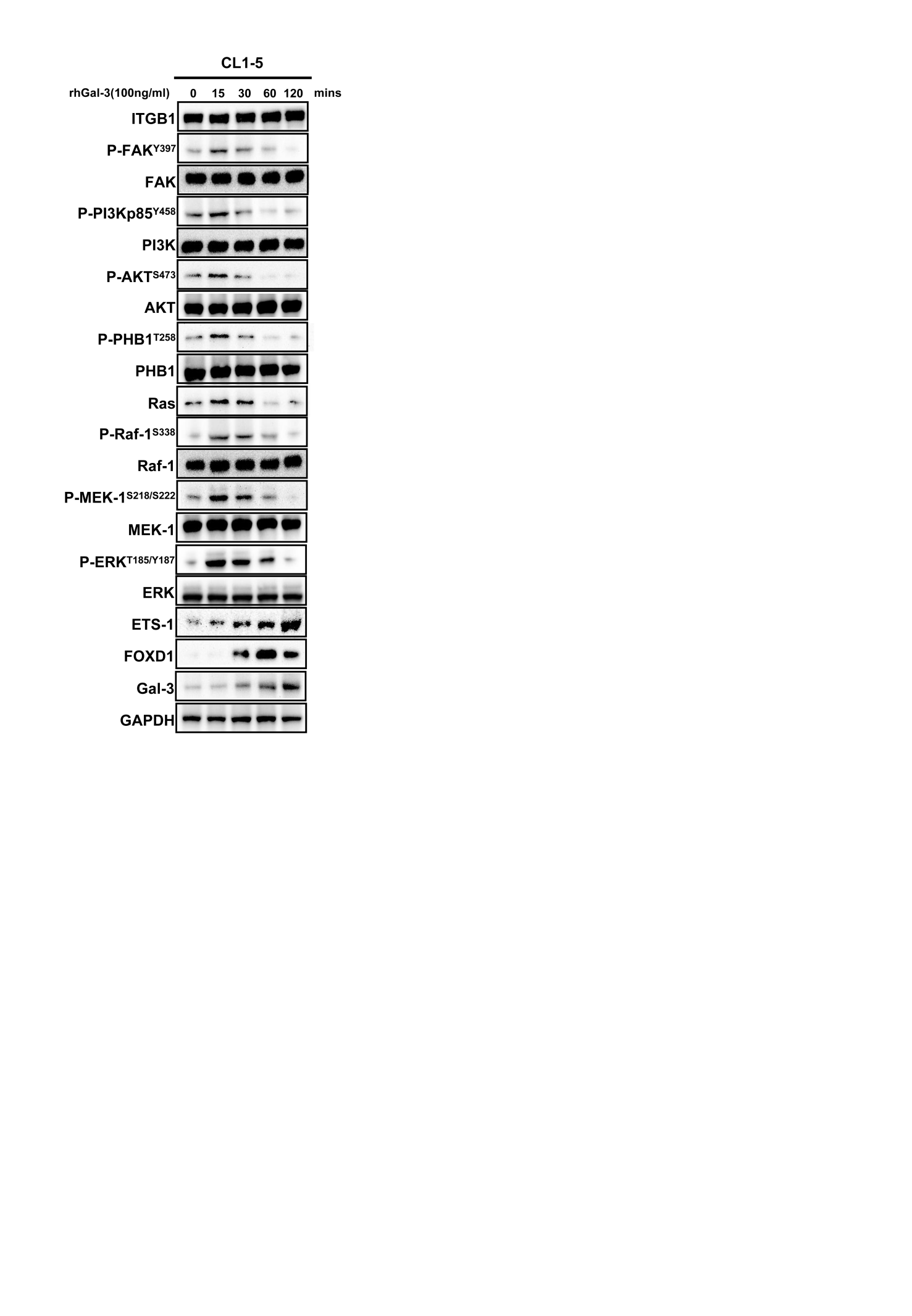
**

**Figure S4.** rhGal-3 regulates ETS-1/FOXD1 through ITGβ1/ERK signaling. CL1-5 cells were treated with rhGal-3 for the indicated times. Cells lysates were collected and analyzed by immunoblotting.


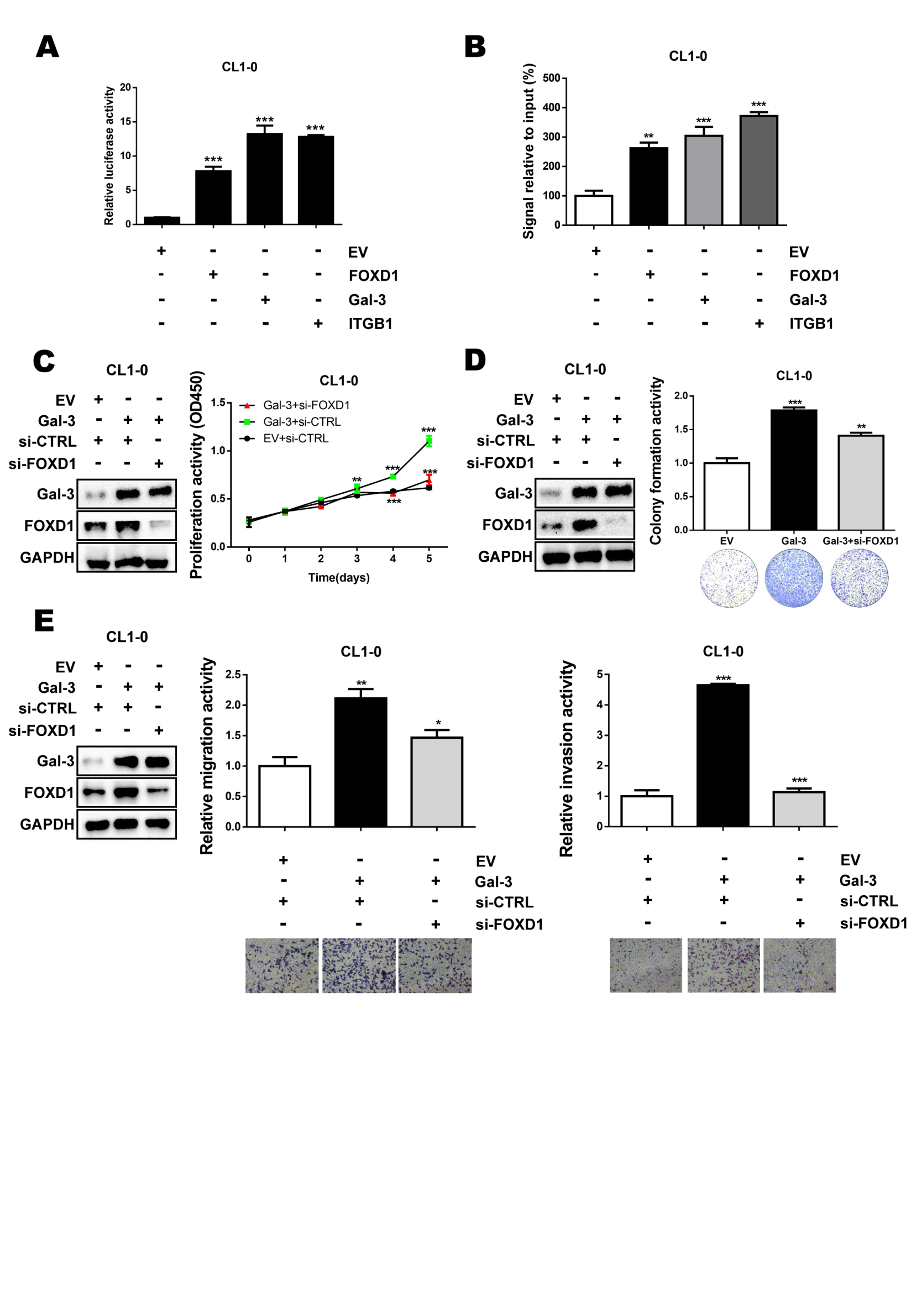


**Figure S5.** FOXD1 is required for Gal-3-mediated cell growth and motility. CL1-0 cells were transfected with pGL3-*Gal-3* reporter vector and *FOXD1*, *Gal-3* or *ITGB1* expression vector for 48 h, and subsequently evaluated by luciferase activity (a), and ChIP assay (b). CL1-0 cells were transfected with *Gal-3* expression vector or si-*FOXD1*. (c) Proliferation ability was analyzed by CCK-8. (d) Representative images of colony-forming assay (e) Representative images of the migration and invasion assays.

**
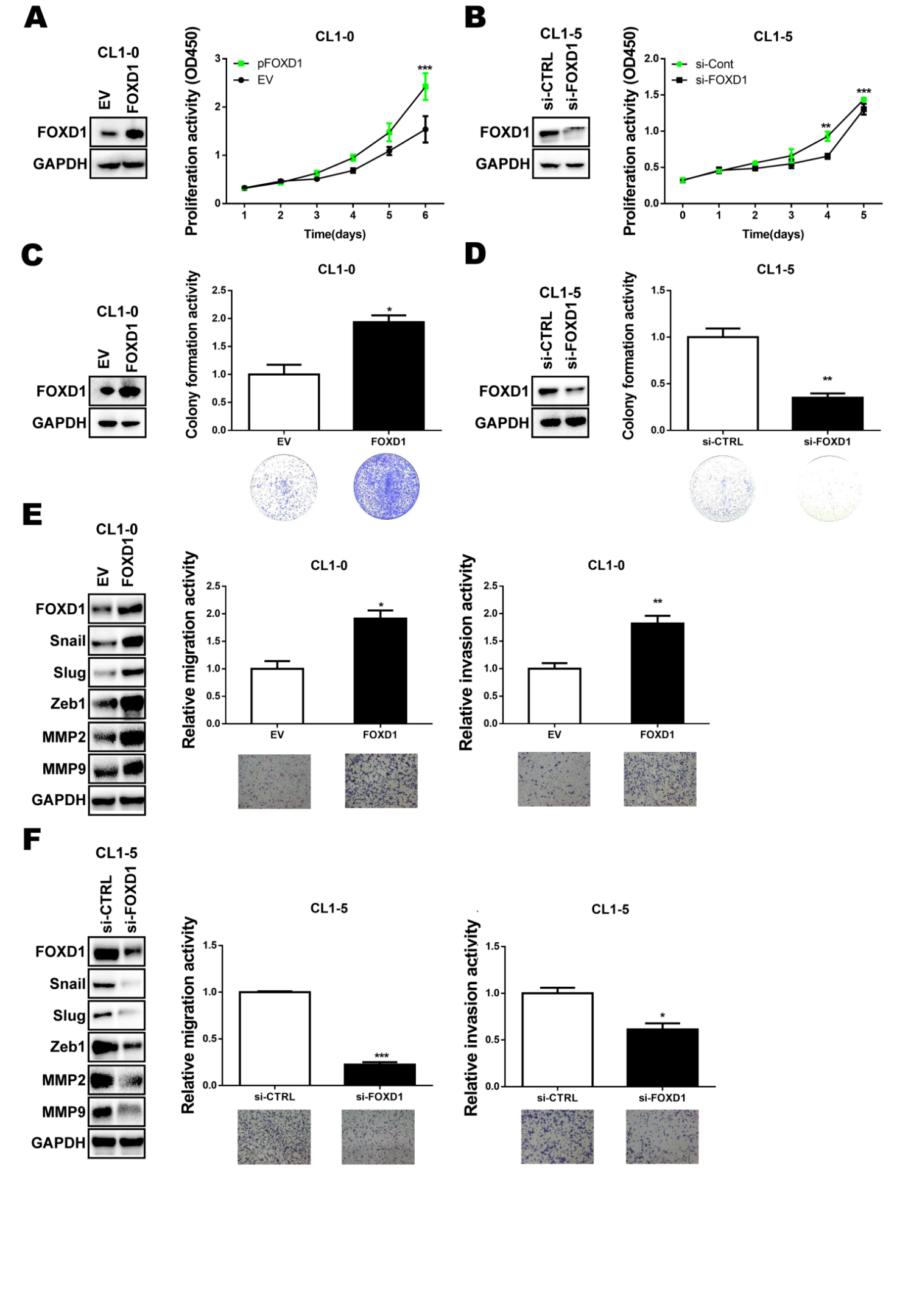
Figure S6.** FOXD1 promotes growth and motility of lung cancer cells. CL1-0 or CL1-5 cells were transfected with the empty vector or plasmid of *FOXD1* (*pFOXD1*) for 48 h or scramble and siRNA targeting *FOXD1* (*si-FOXD1*) for 72 h, respectively as indicated. Protein expression was analyzed by Western blotting. (a, b) Proliferation ability was analyzed by CCK-8 assay. (c, d) Representative images of colony-forming assay (e, f) Representative images of migration and invasion assay.

**
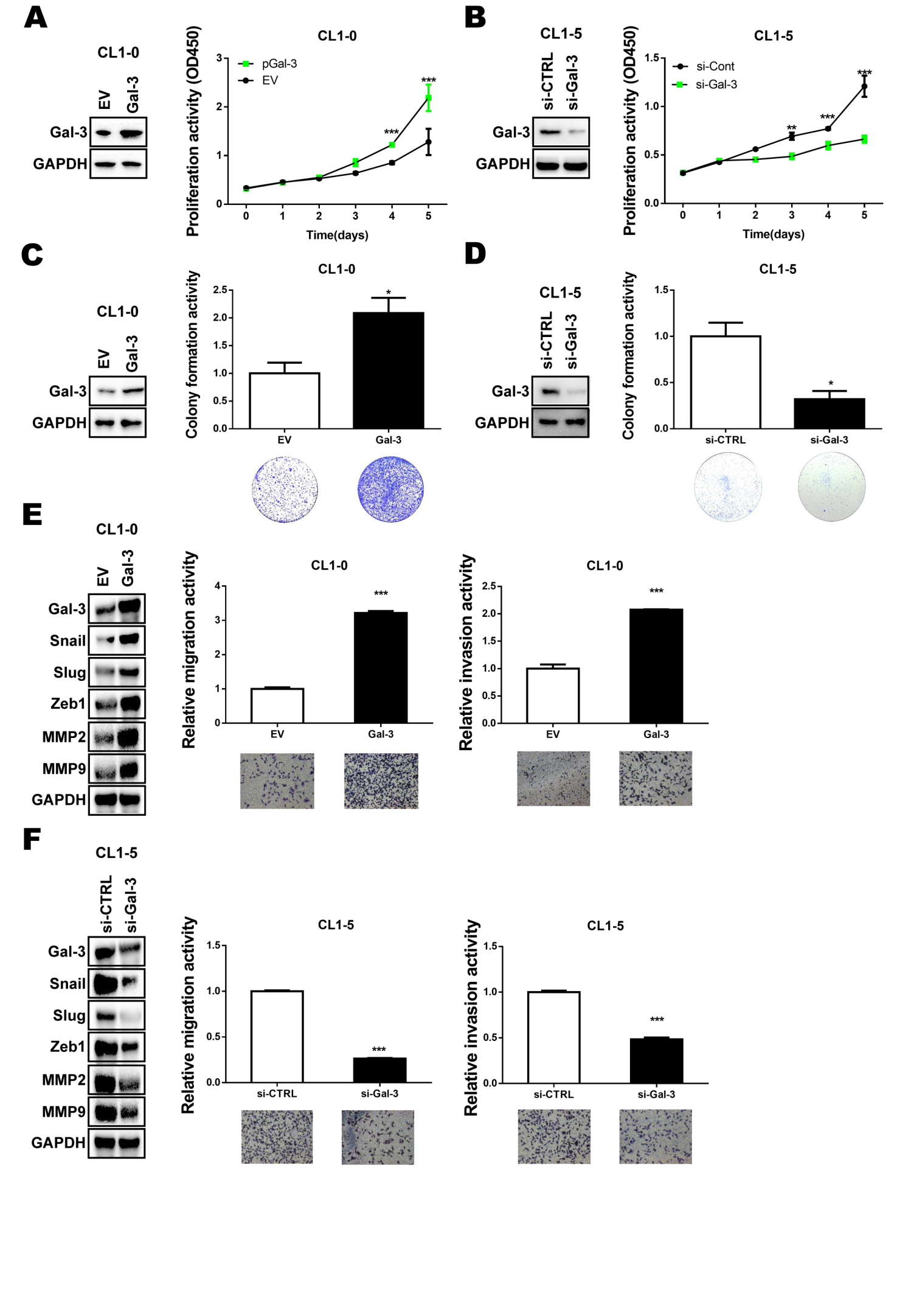
**

**Figure S7.** Gal-3 promotes growth and motility of lung cancer cells. CL1-0 or CL1-5 cells were transfected with the empty vector or plasmid of *Gal-3* (*pGal-3*) for 48 h or scramble and siRNA targeting *Gal-3* (*si-Gal-3*) for 72 h, respectively as indicated. Protein expression was analyzed by Western blotting. (a, b) Proliferation ability was analyzed by CCK-8 assay. (c, d) Representative images of colony-forming assay. (e, f) Representative images of migration and invasion assay.


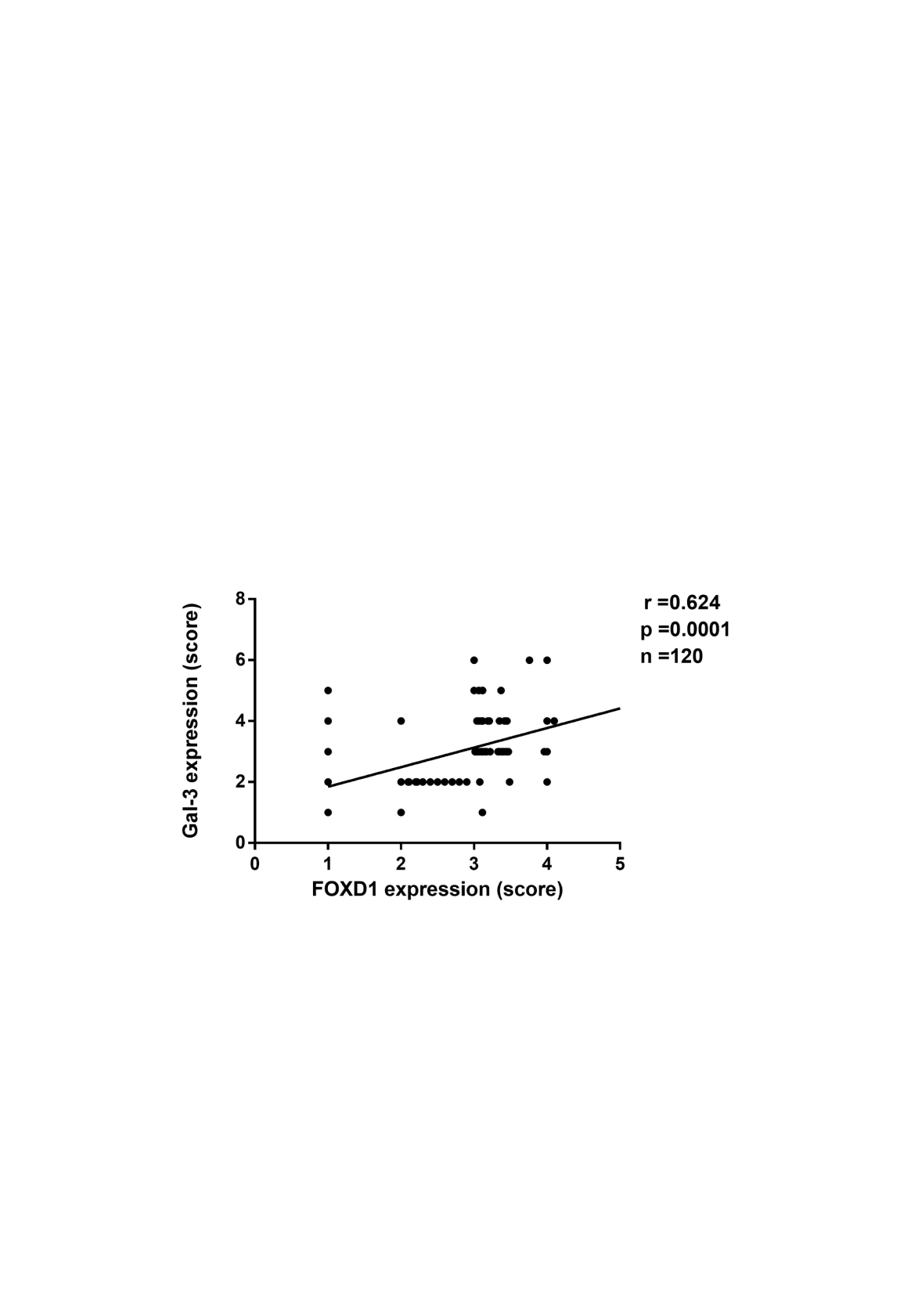


**Figure S8.** Correlation between FOXD1 and Gal-3 expression. FOXD1 expression is positively related to Gal-3 expression (r = 0.624).
