# Supplementary material for: FOXD1 and Gal-3 Form a Positive Regulatory Loop to Regulate Lung Cancer Aggressiveness
Source: Cancers (Basel). 2019 Nov 28;11(12):1897. doi: 10.3390/cancers11121897 (PMC6966623; doi:10.3390/cancers11121897)

Figure 1D
RAW data

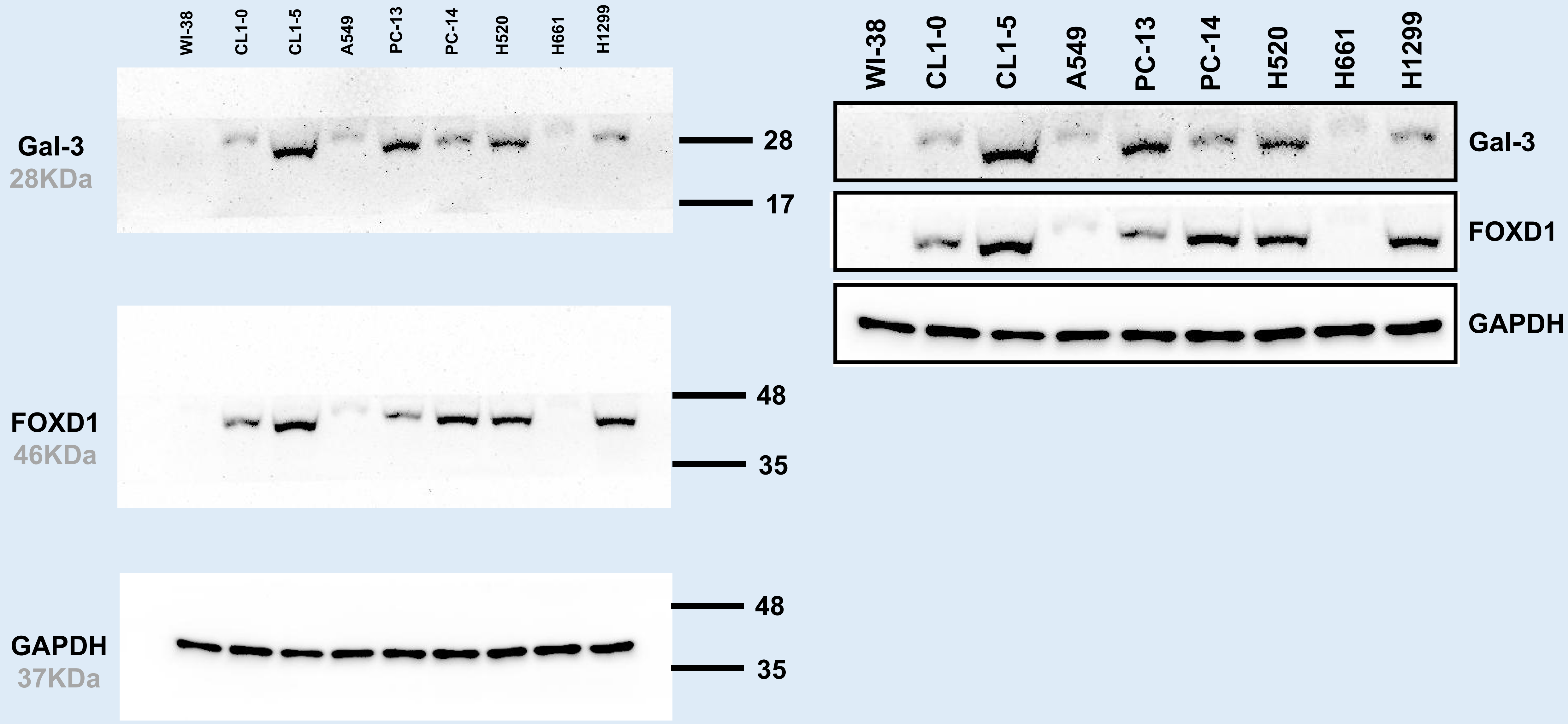

Figure 2C
RAW data

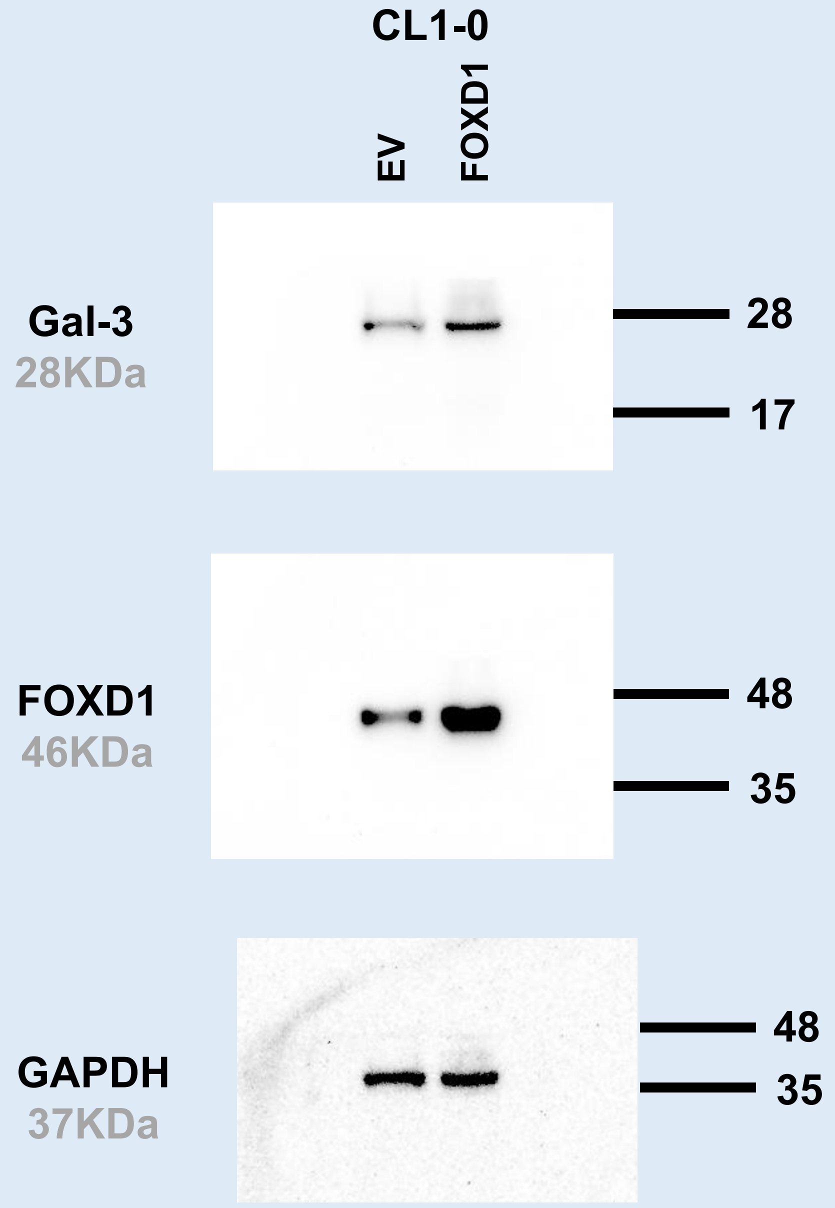

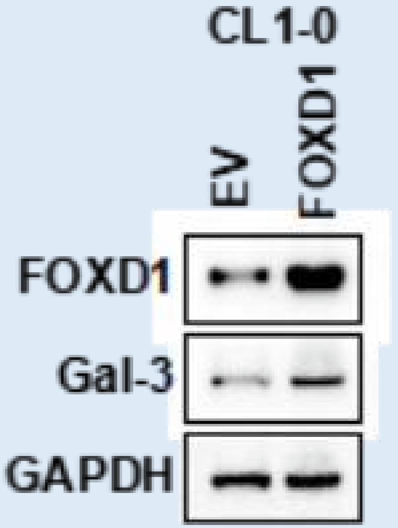

Figure 2D
RAW data

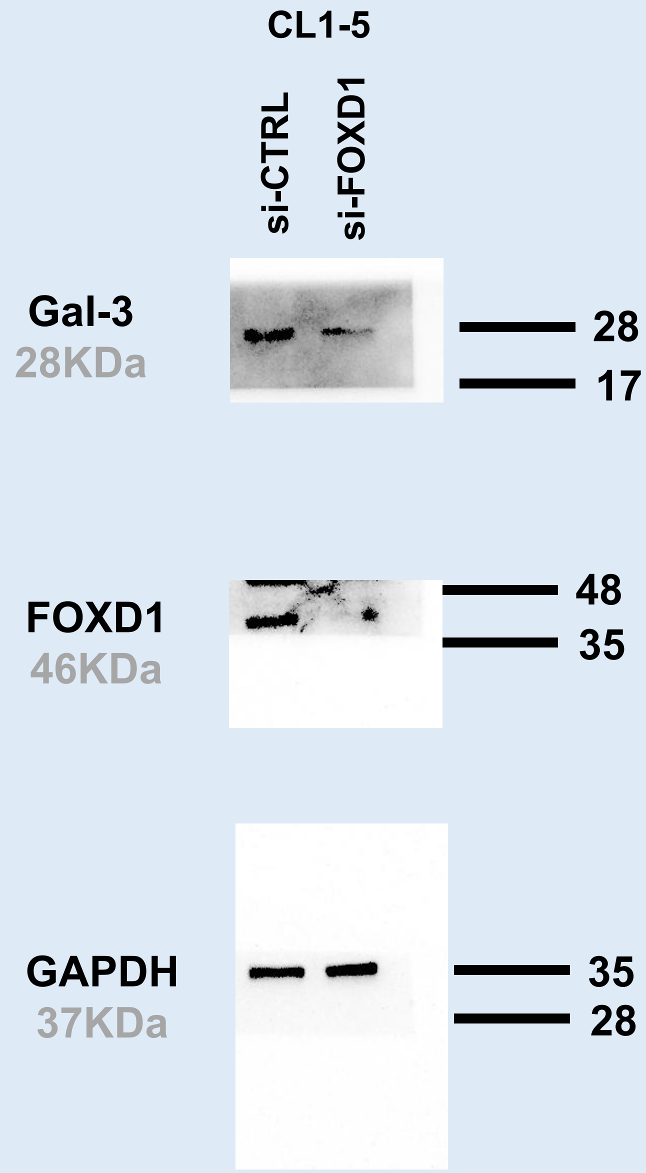

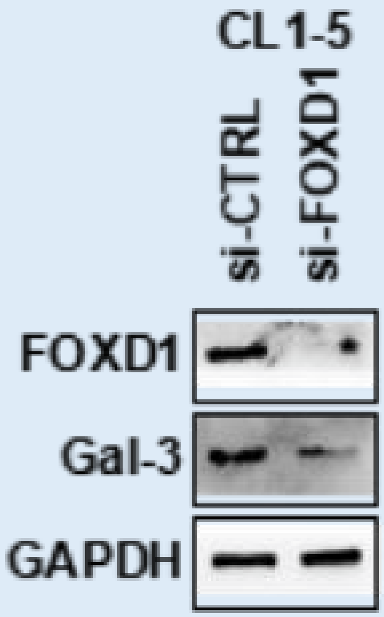

Figure 2E
RAW data

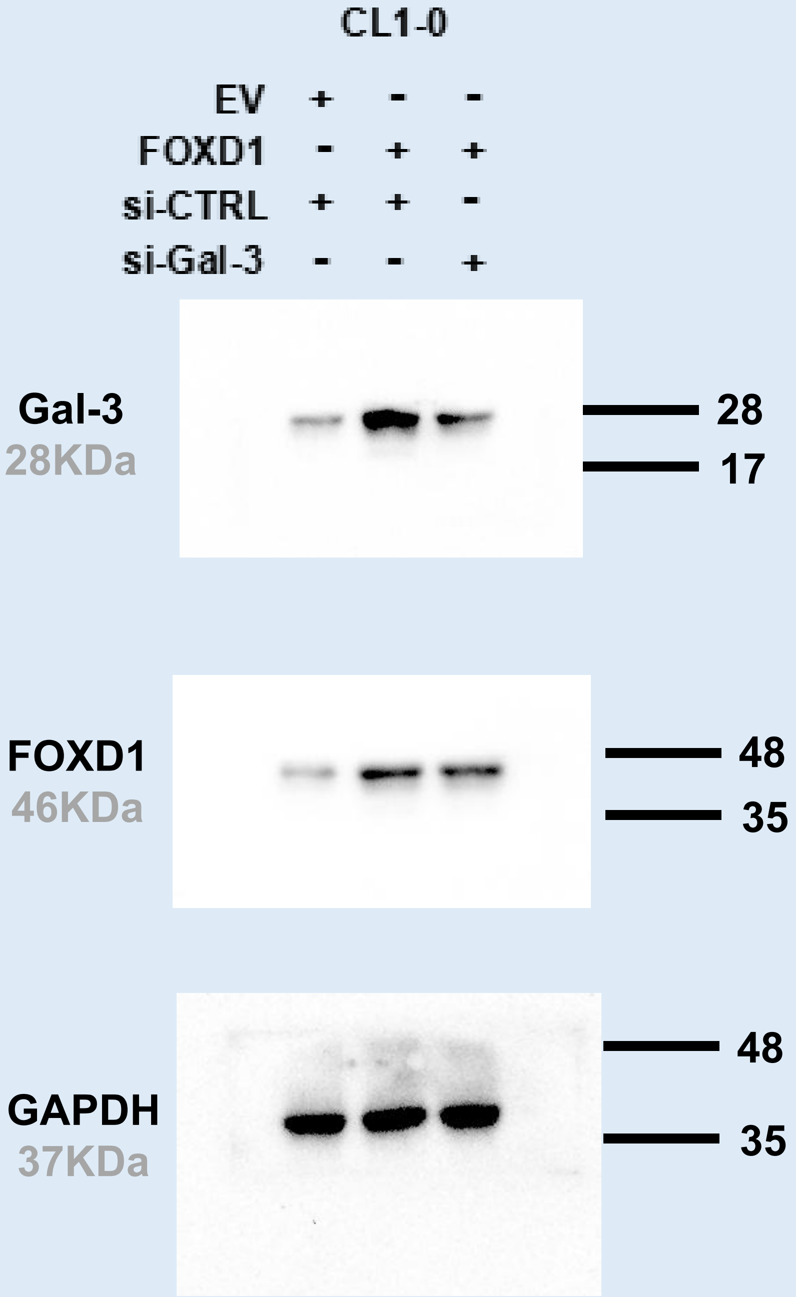

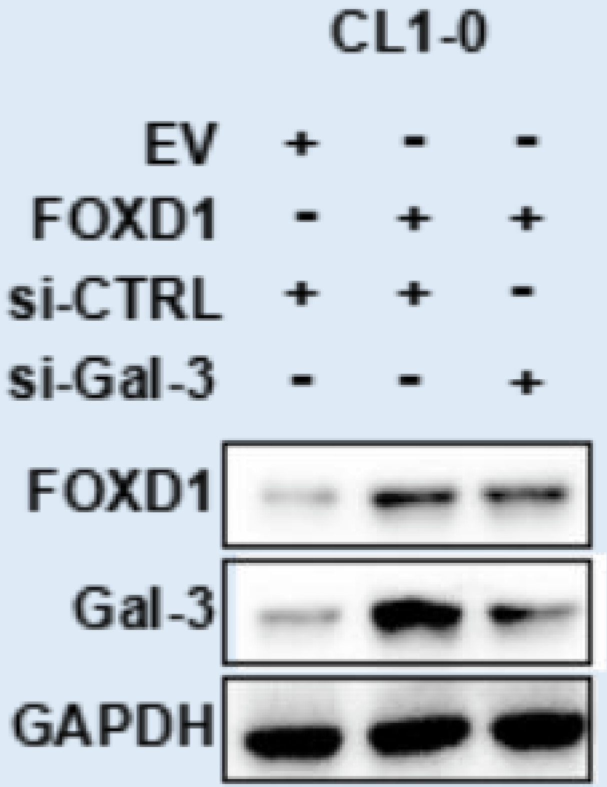

Figure 2F
RAW data

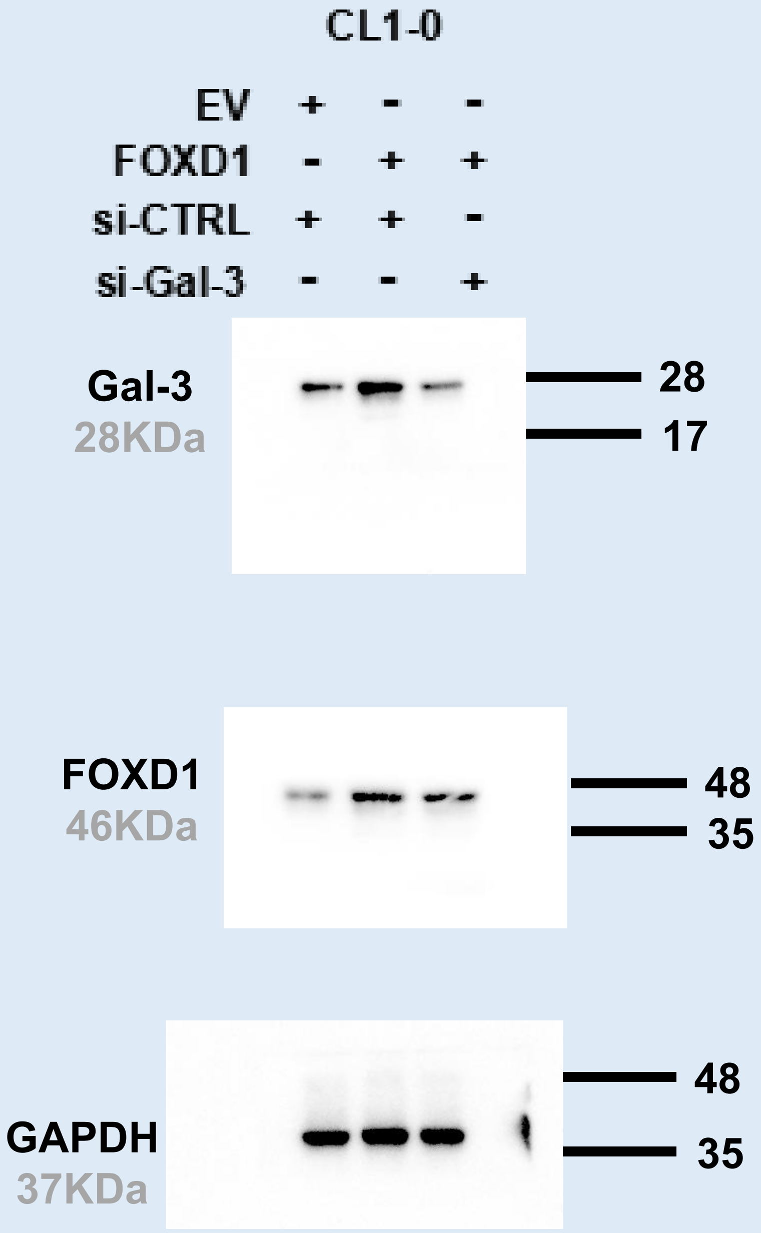

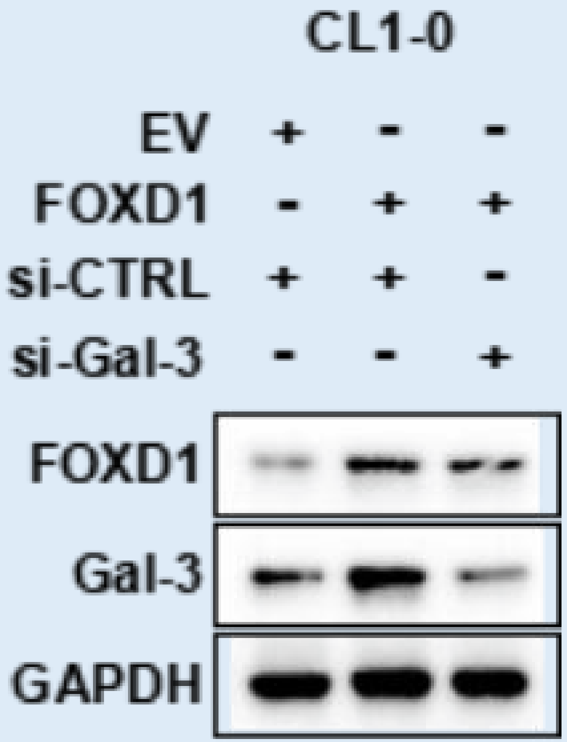

Figure 2G
RAW data

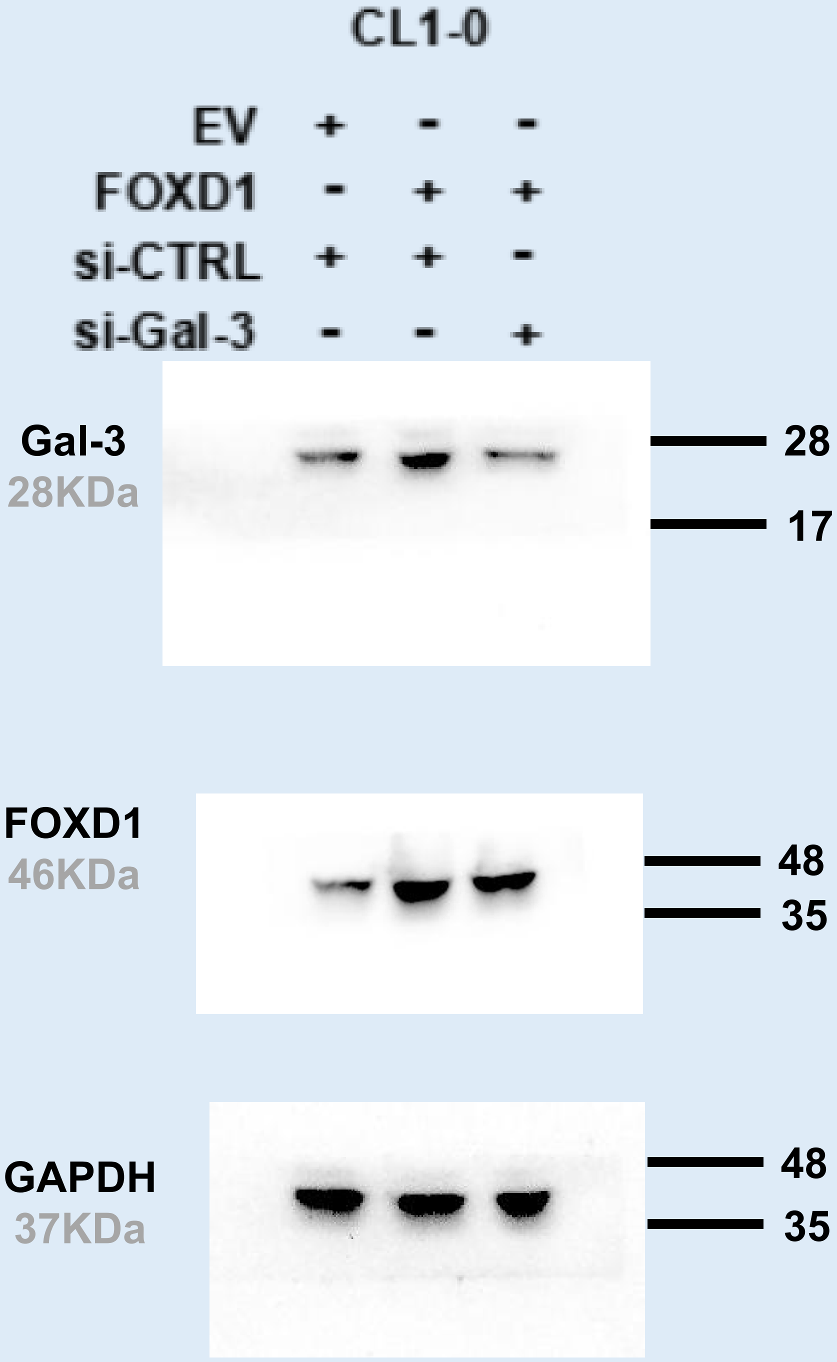

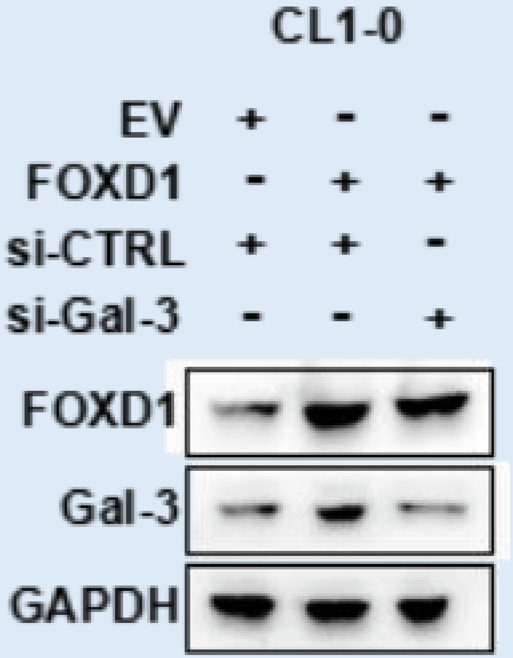

Figure 3D(L)
RAW data

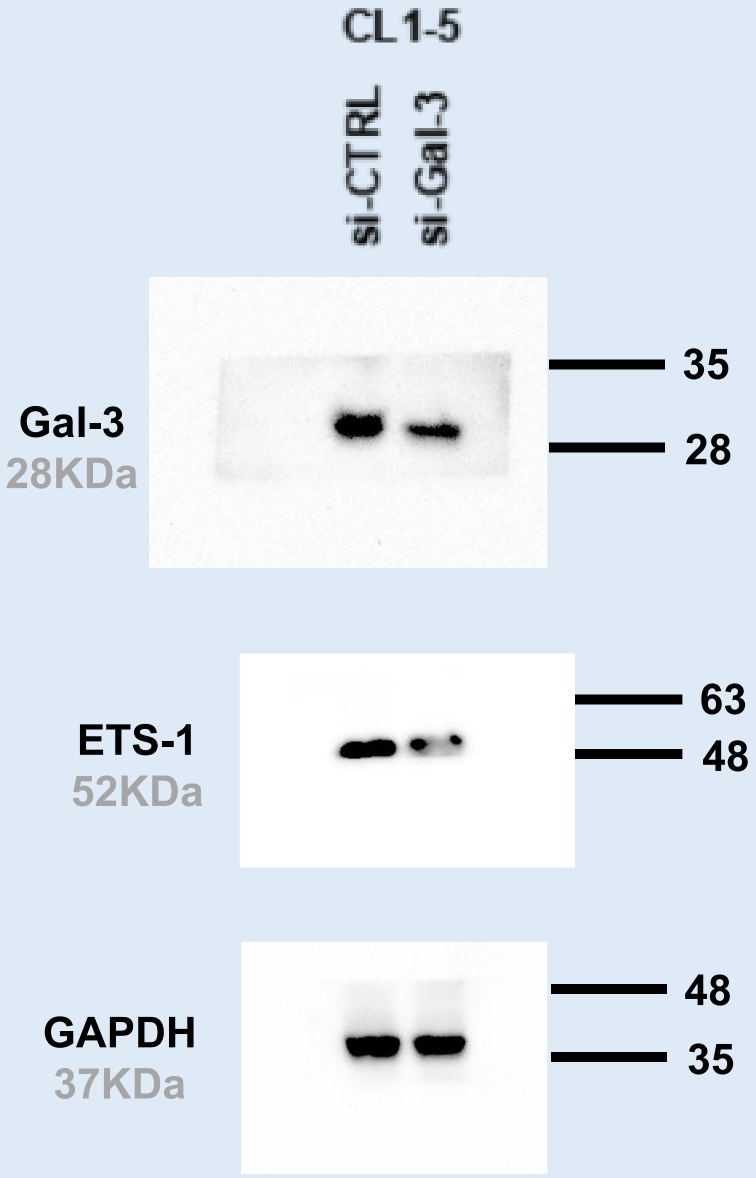

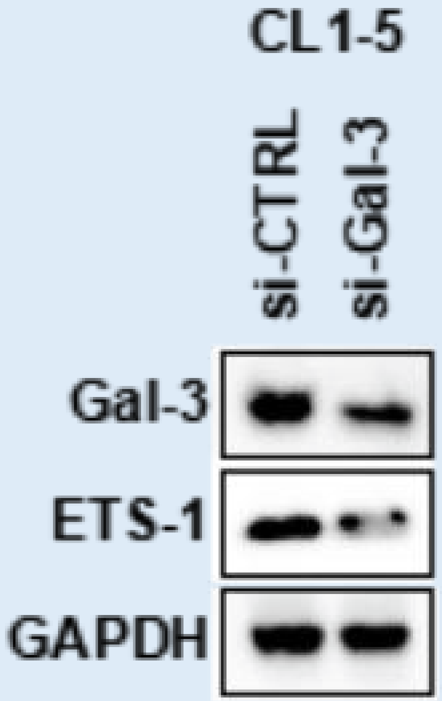

Figure 3D(R)
RAW data

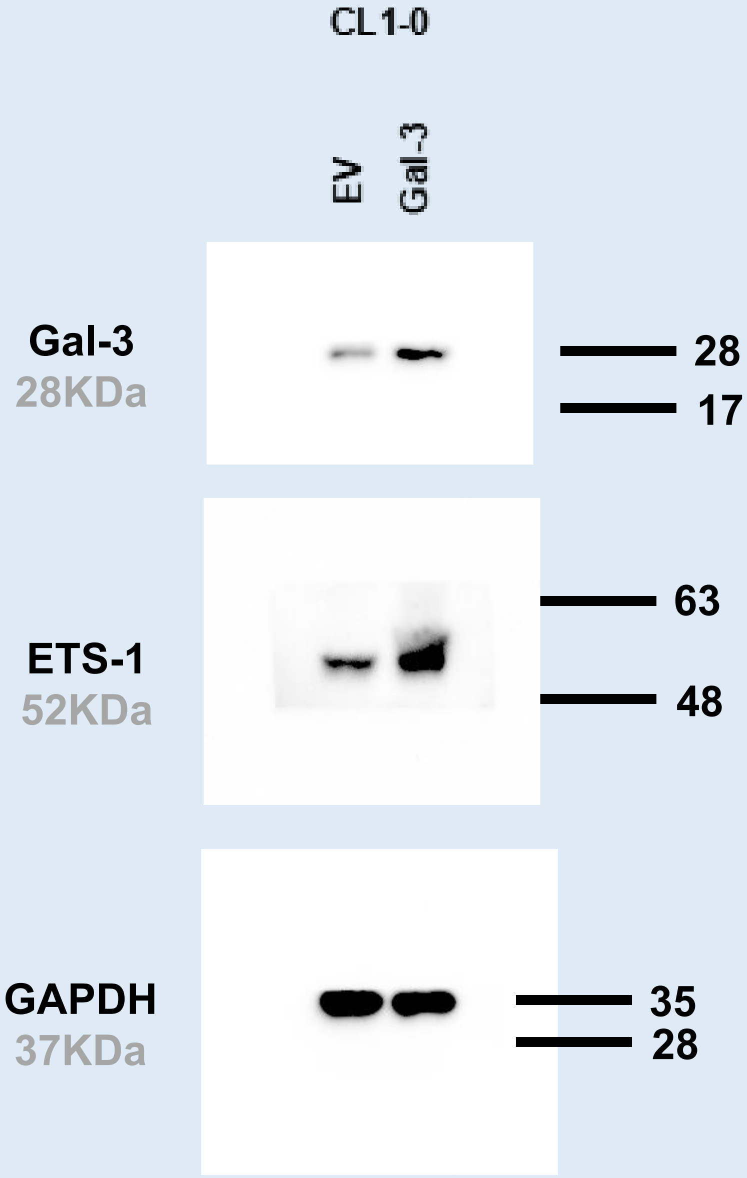

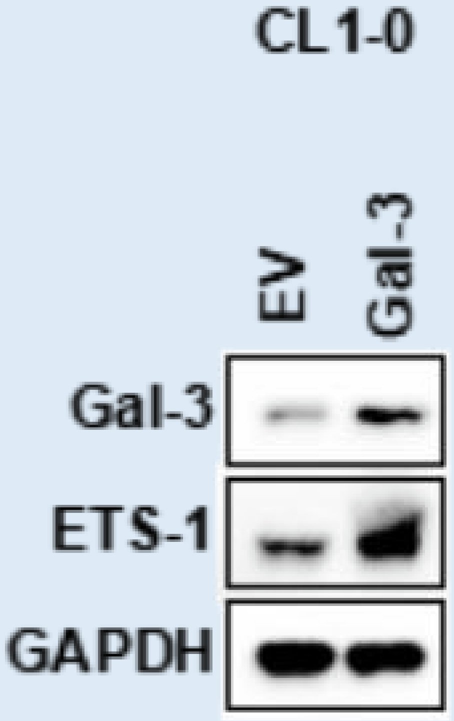

Figure 3F(L)
RAW data

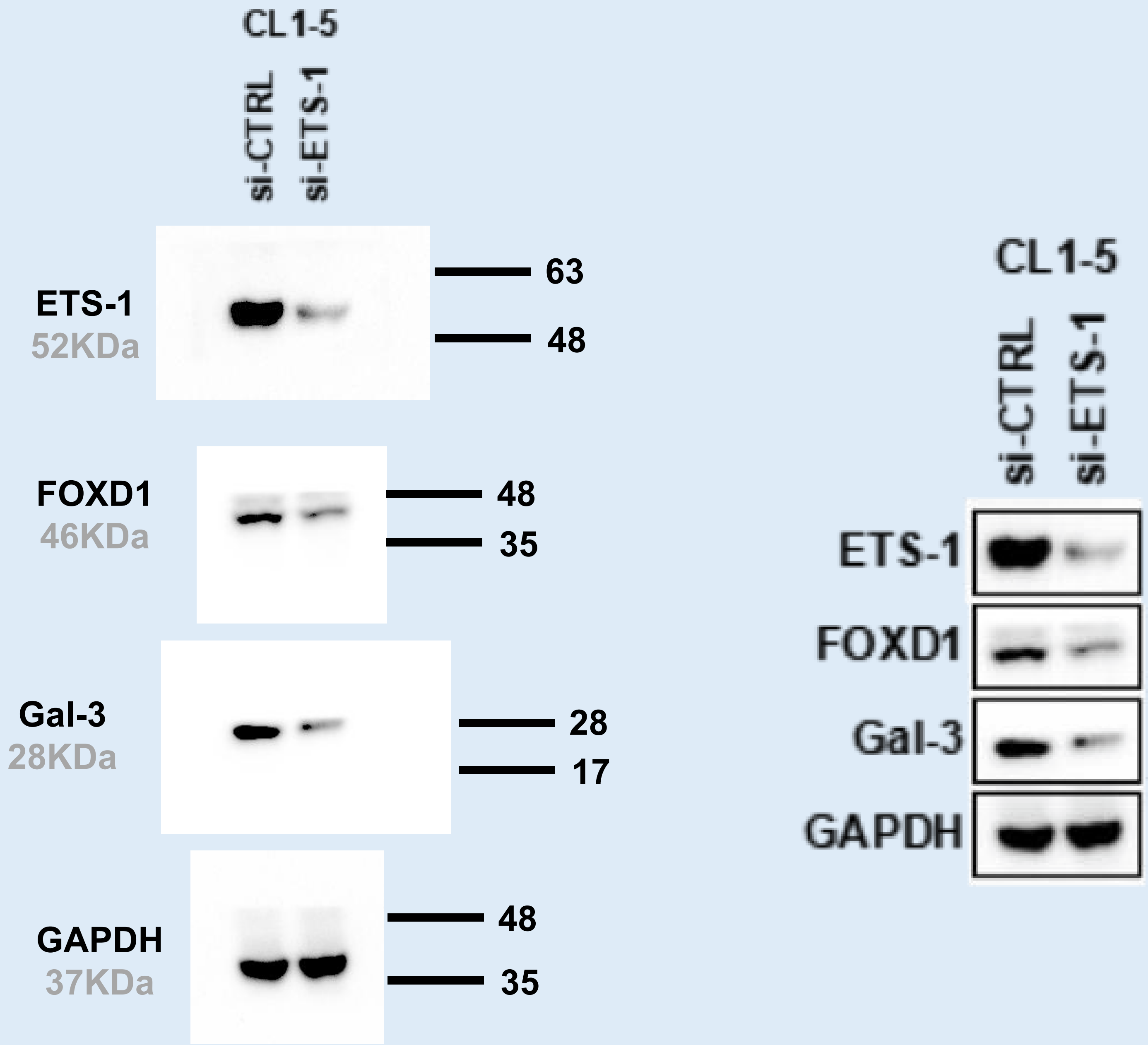

Figure 3F(R)
RAW data

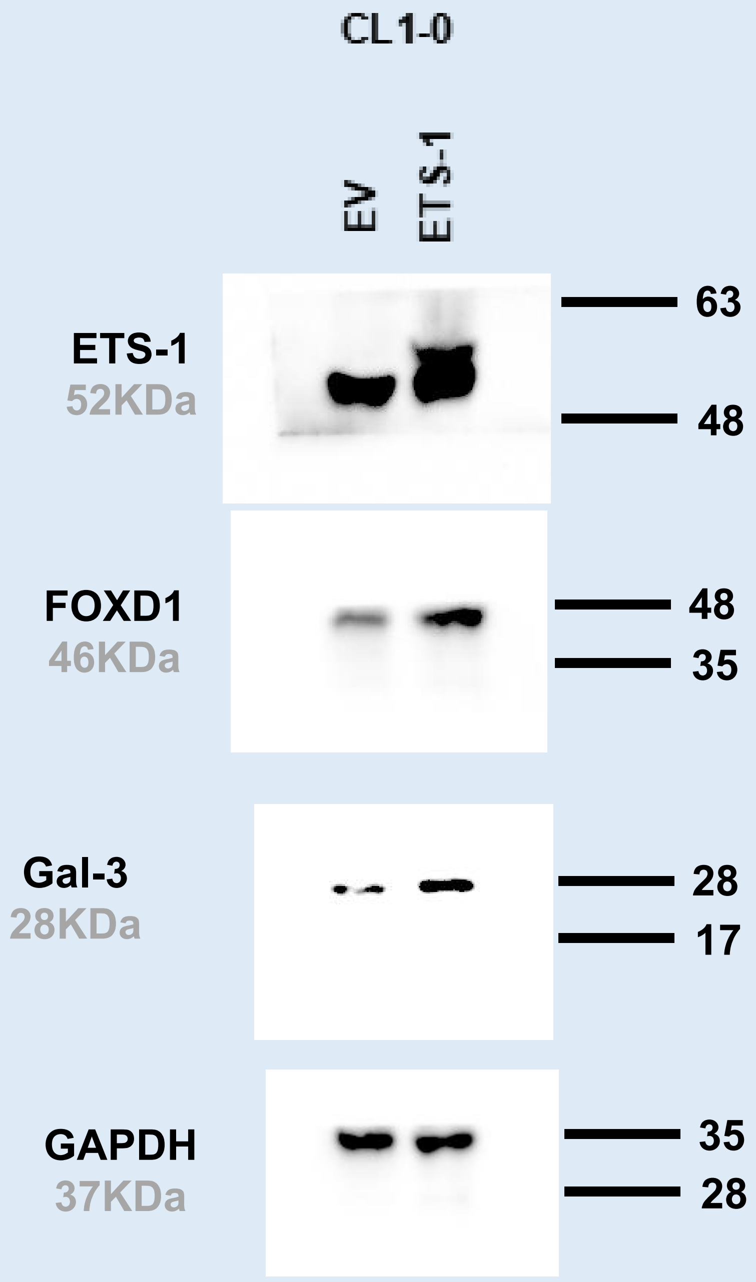

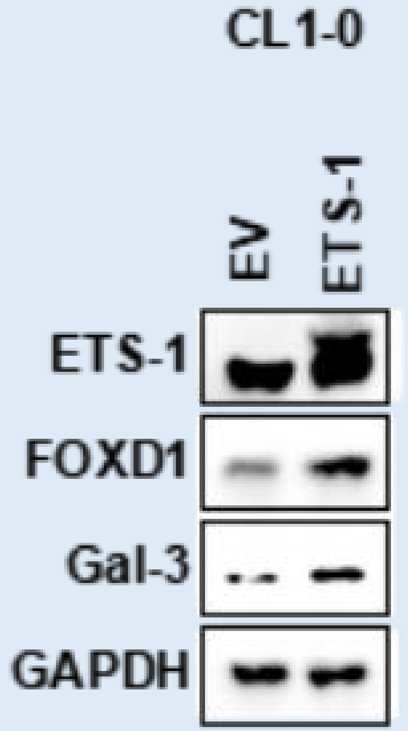

Figure 4A(L)
RAW data

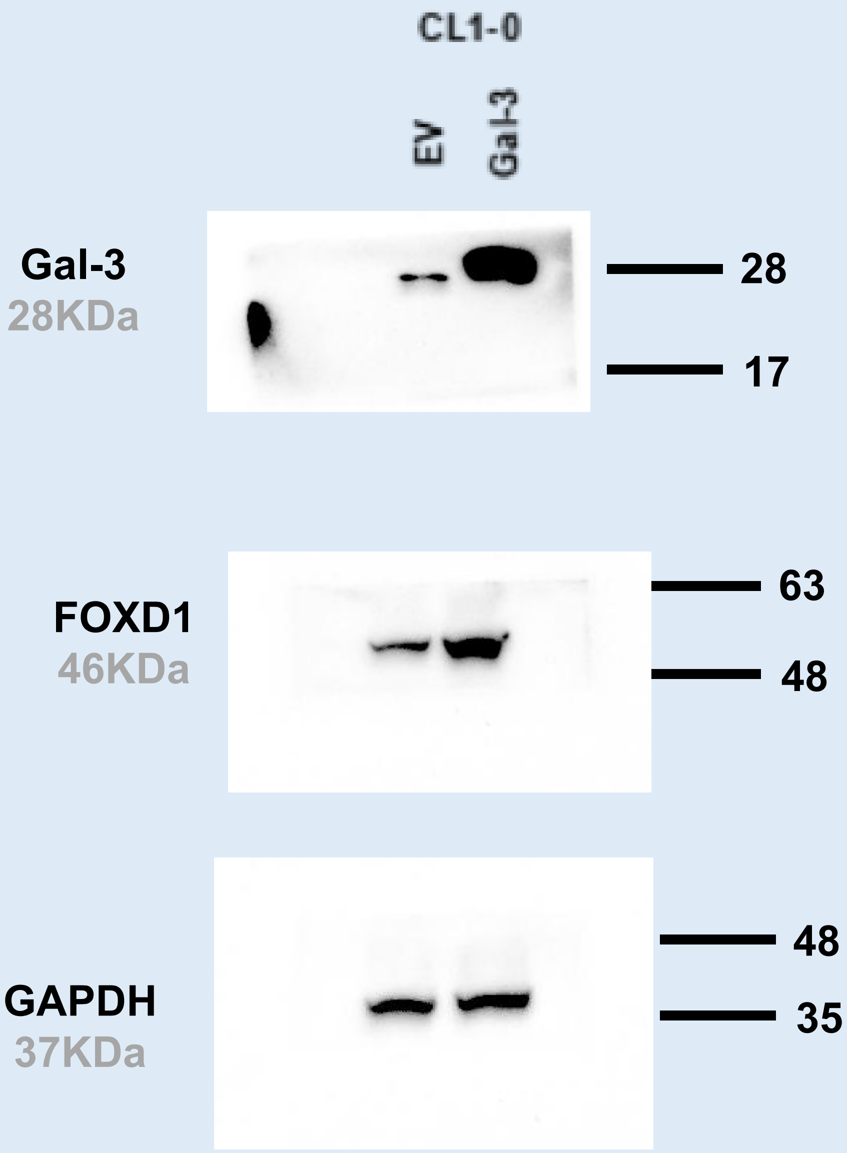

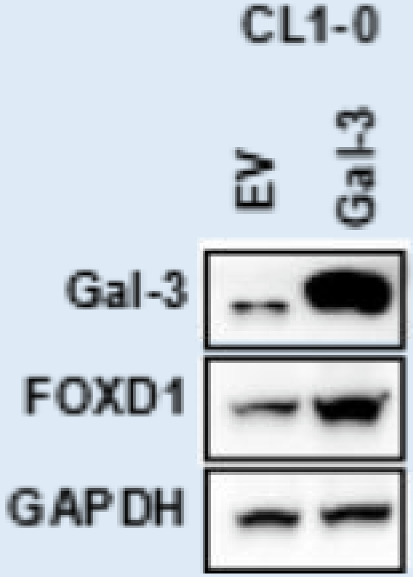

Figure 4A(R)
RAW data

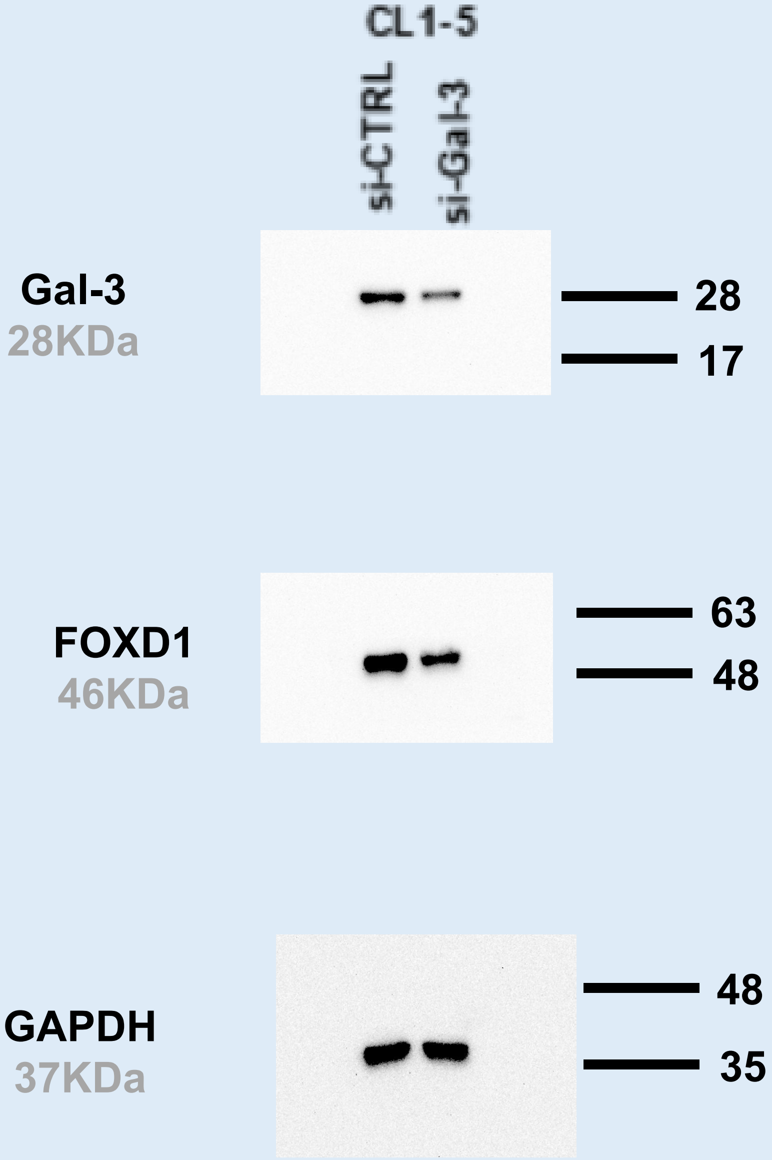

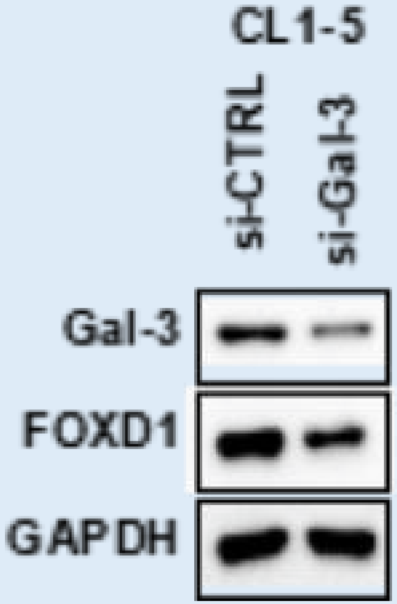

IP Raw data

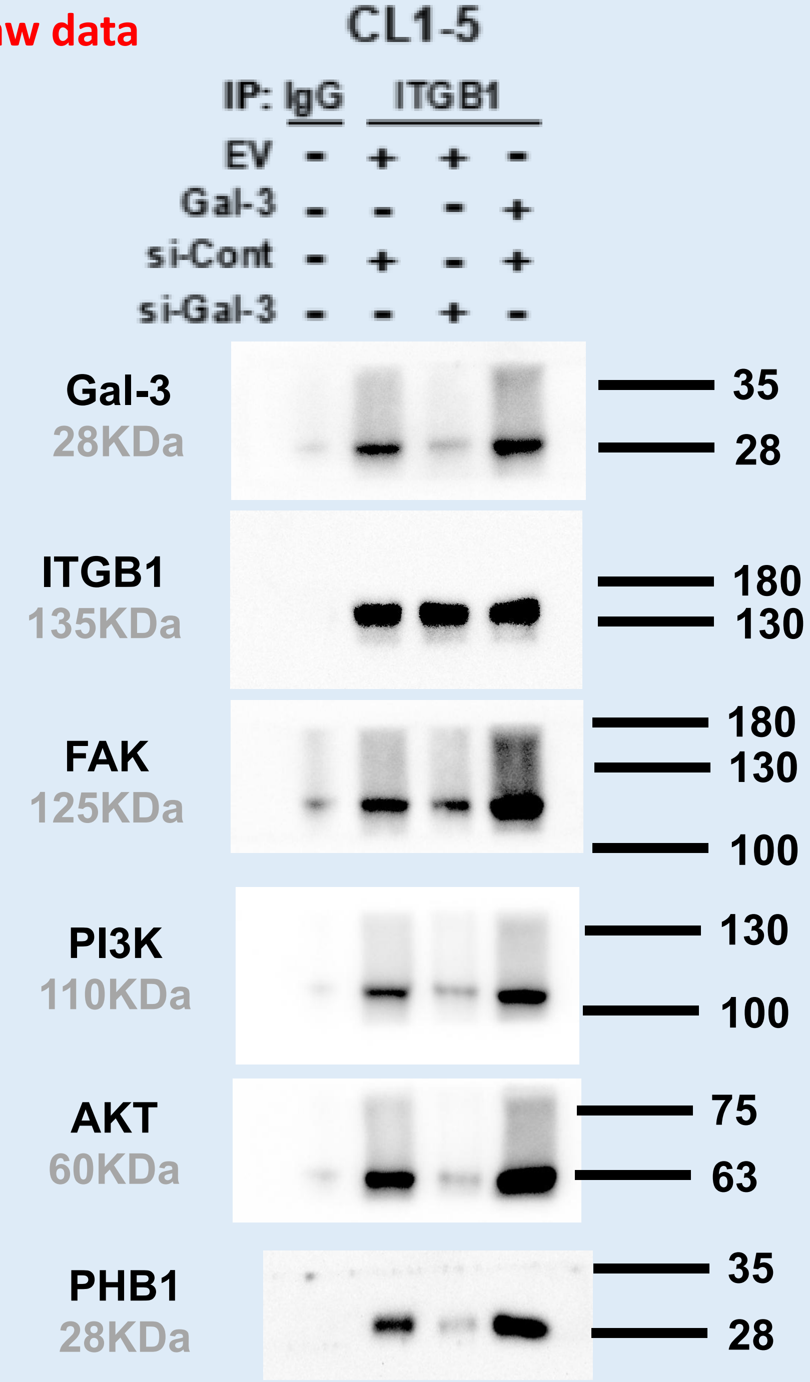

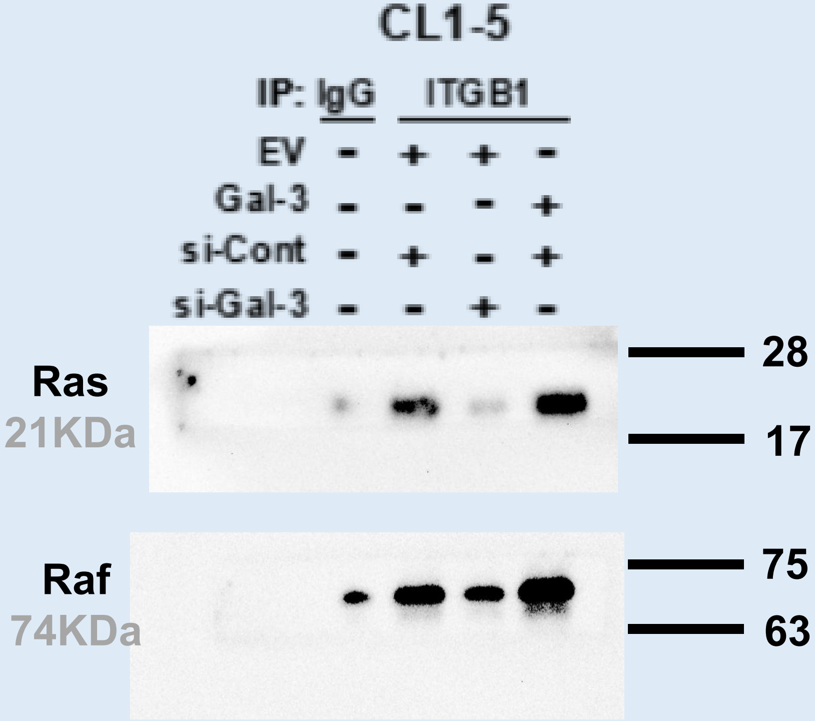

Figure 4B(L) RAW data

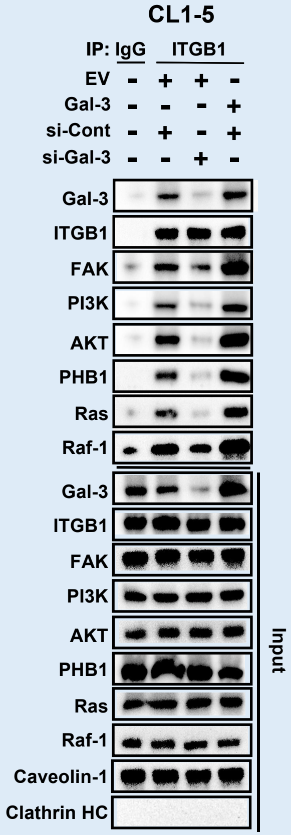

Input Raw data

Figure 4B(L) RAW data

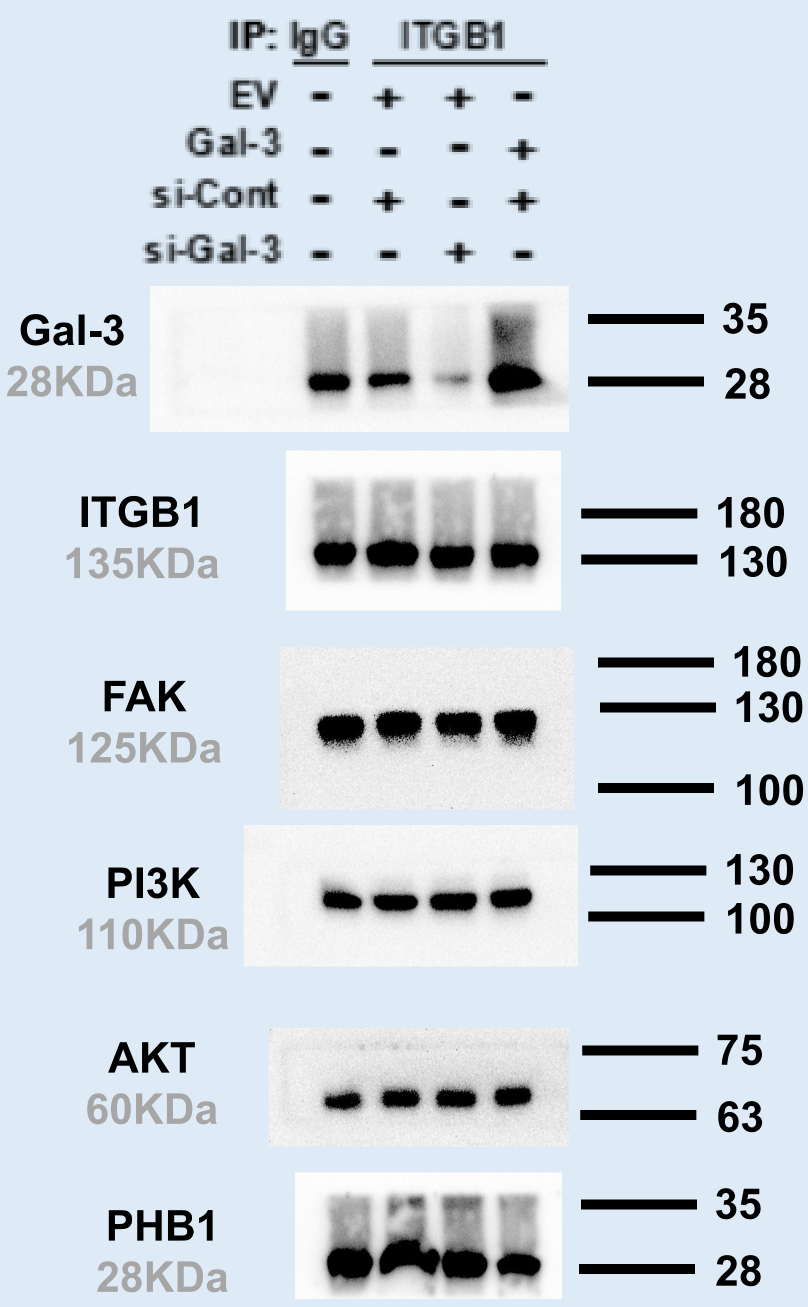

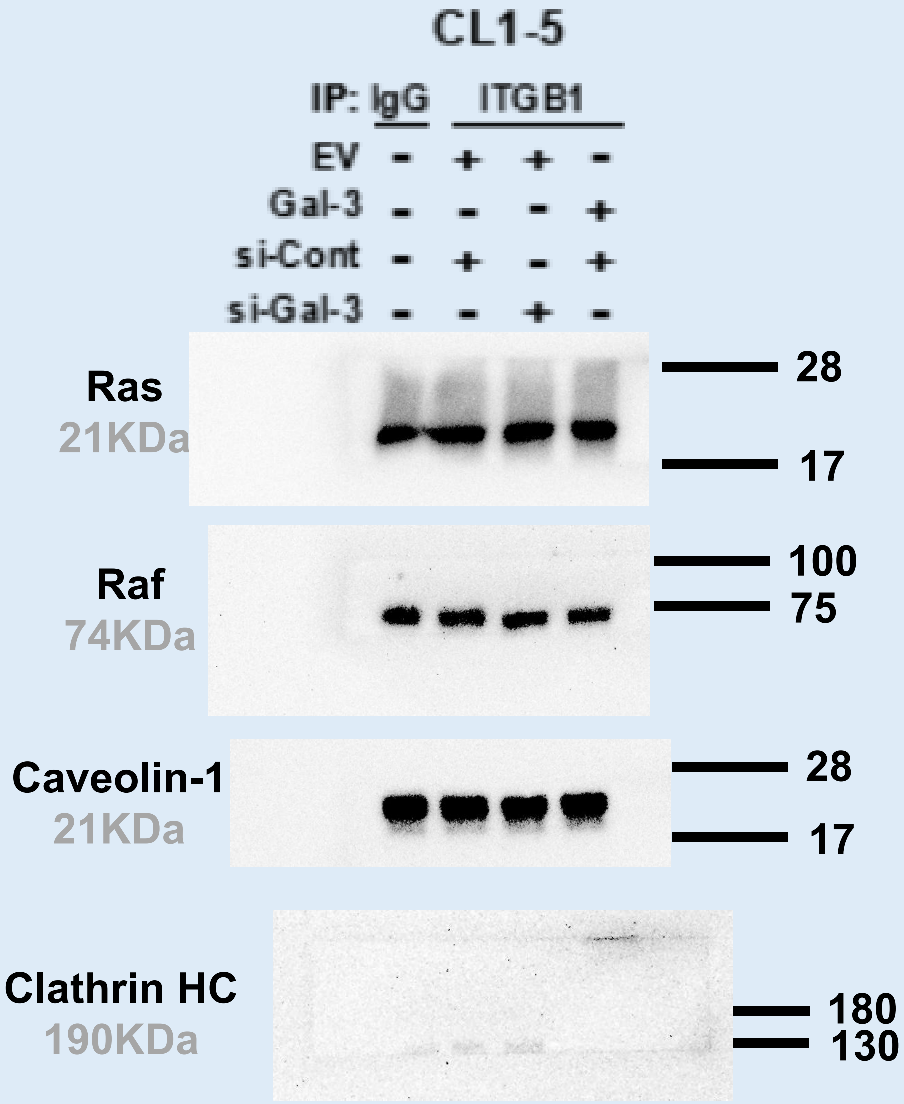

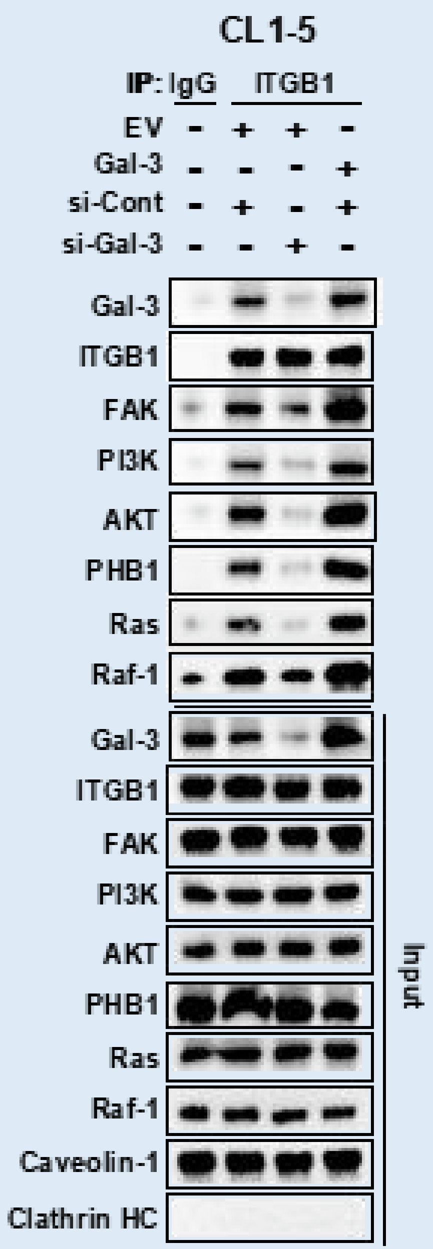

Figure 4B(R)
RAW data

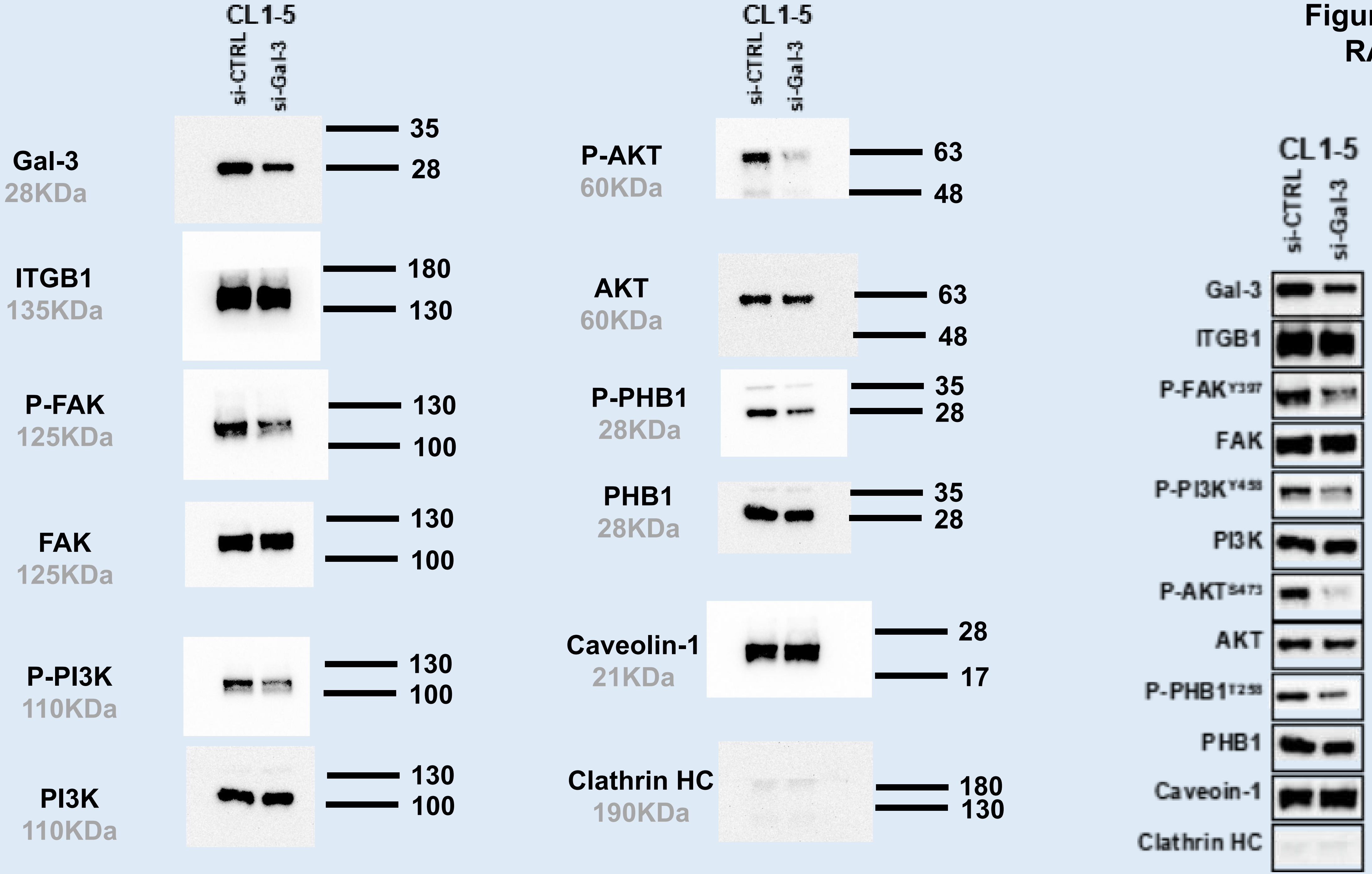

Figure 4C(L) RAW data

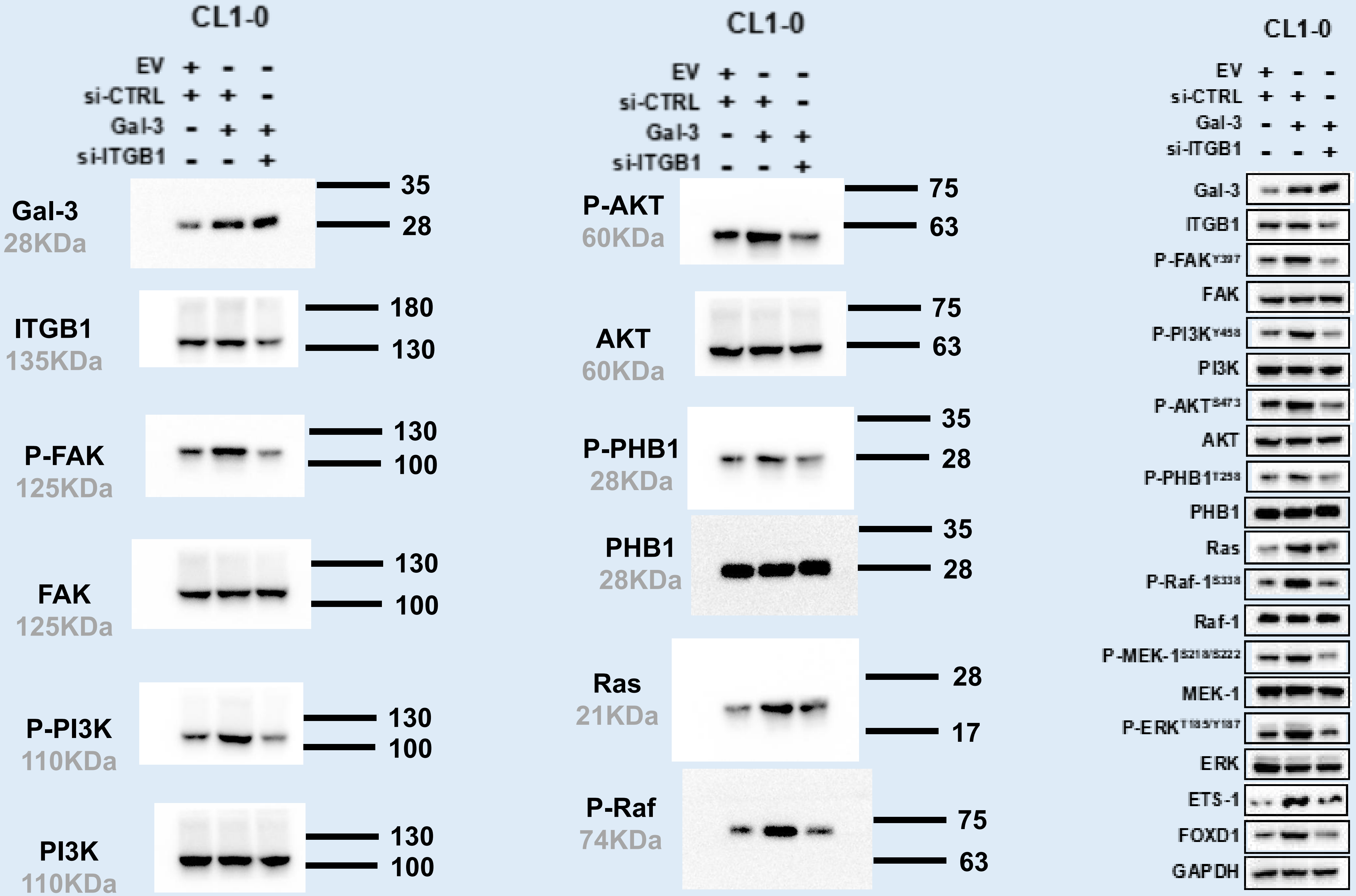

Figure 4C(L)
RAW data

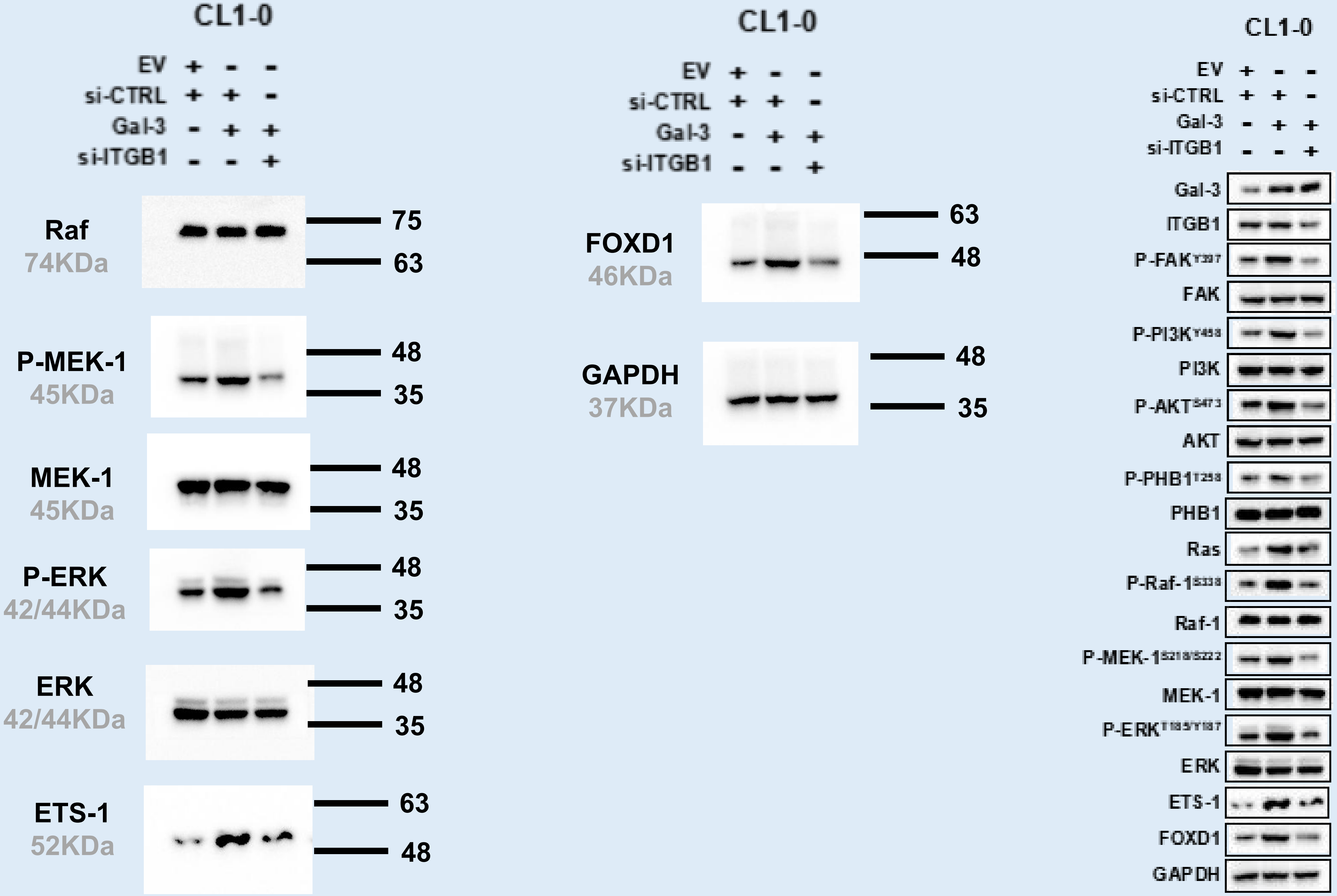

Figure 4C(R)
RAW data

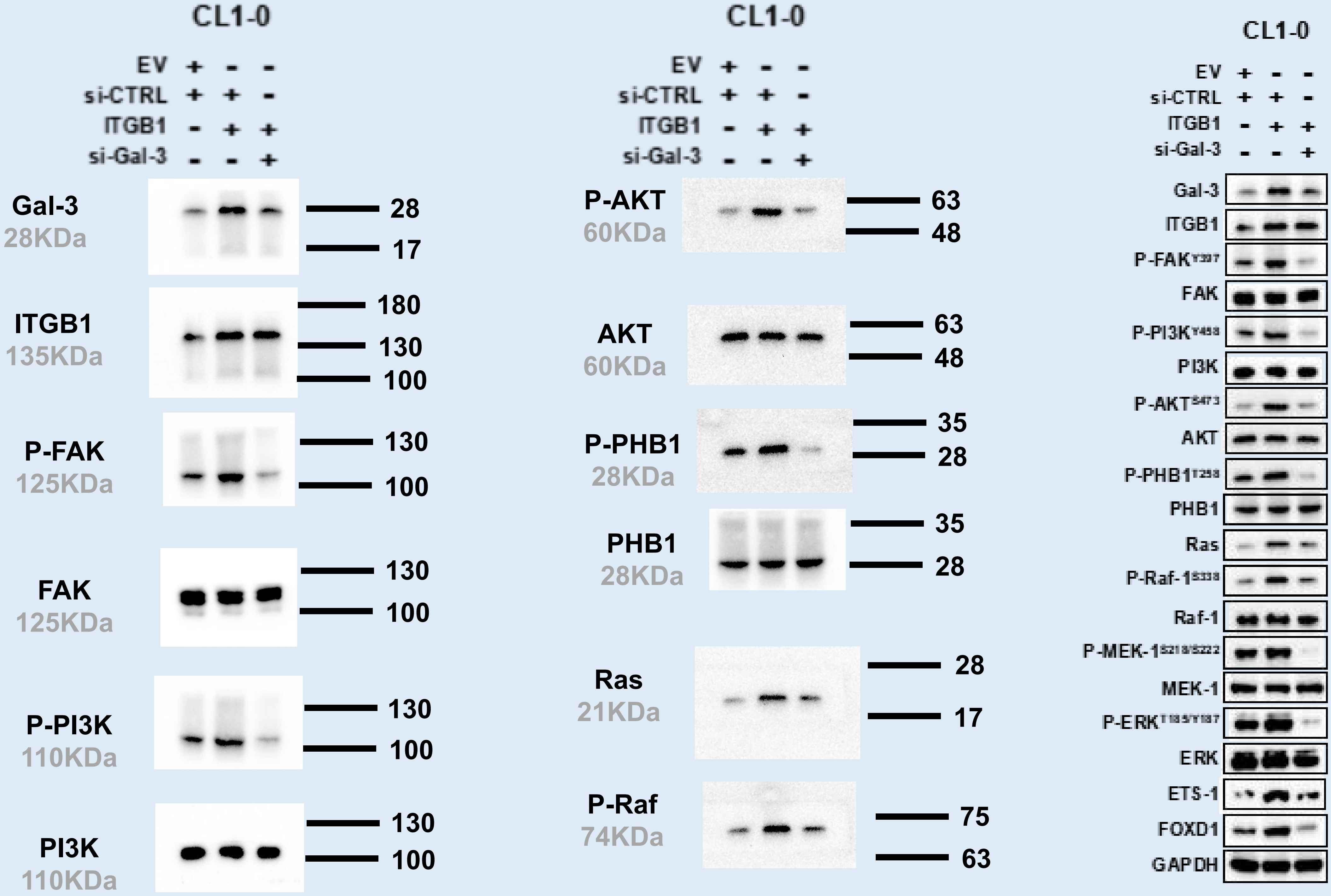

Figure 4C(R)
RAW data

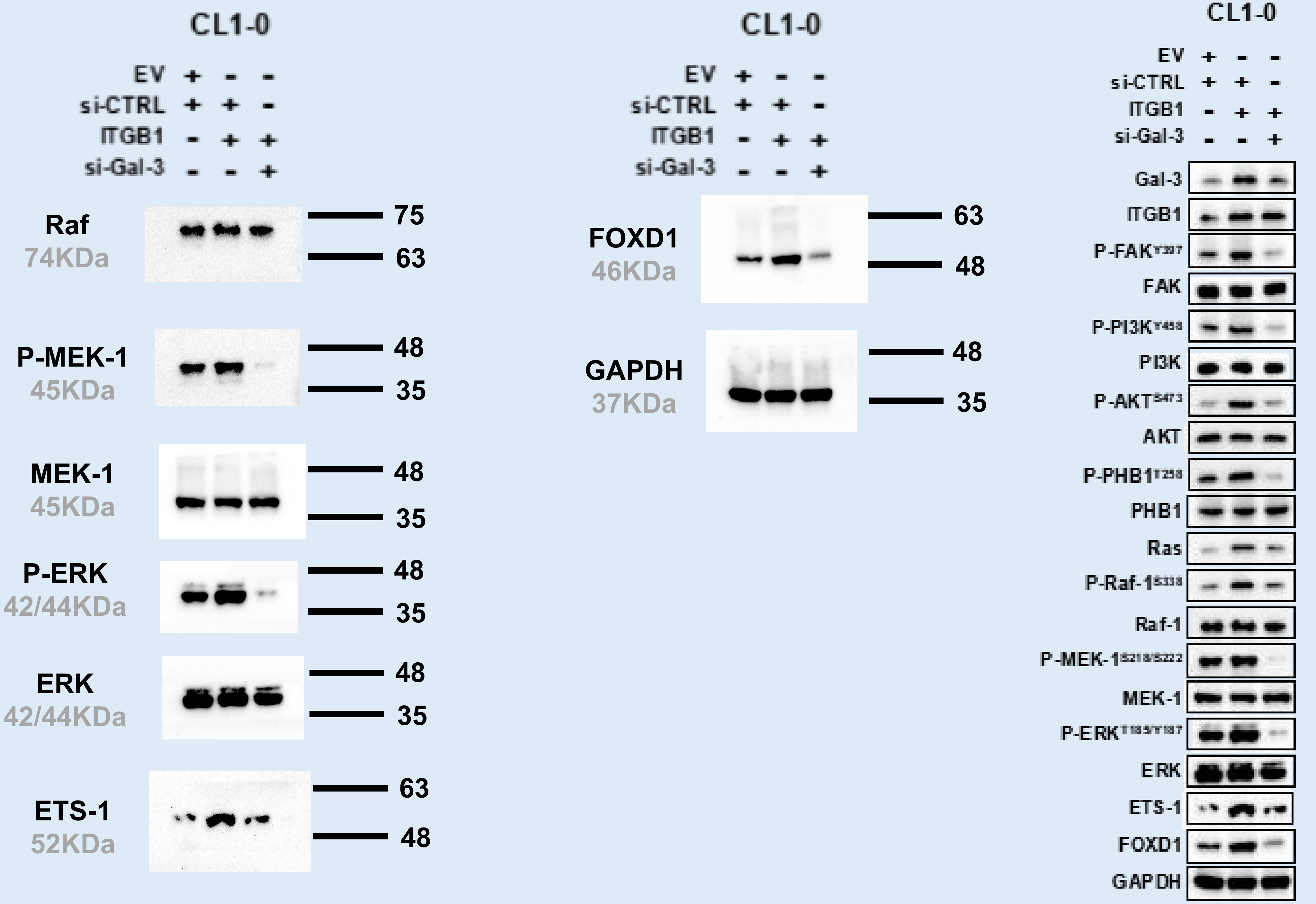

Figure 4D(L) RAW data

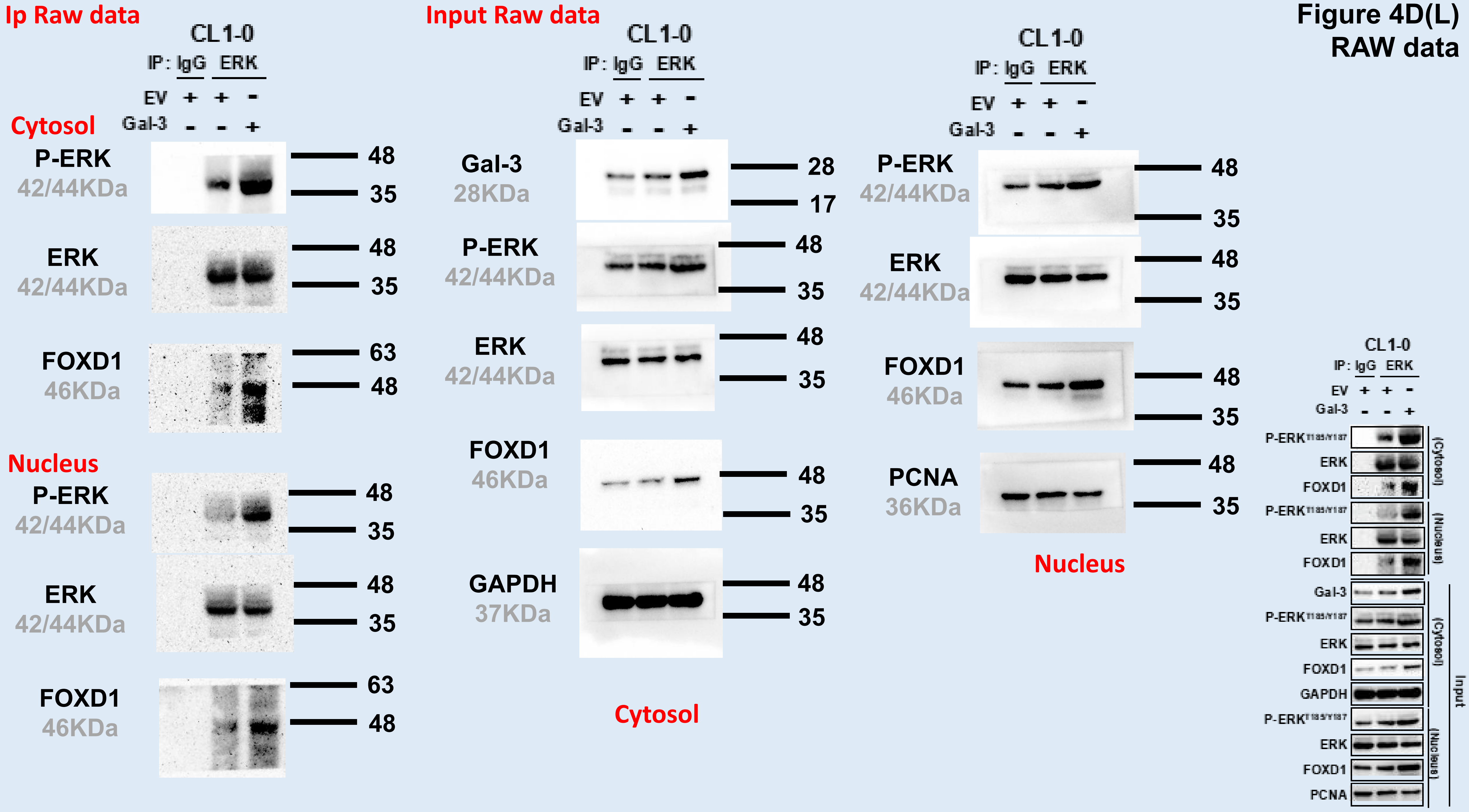

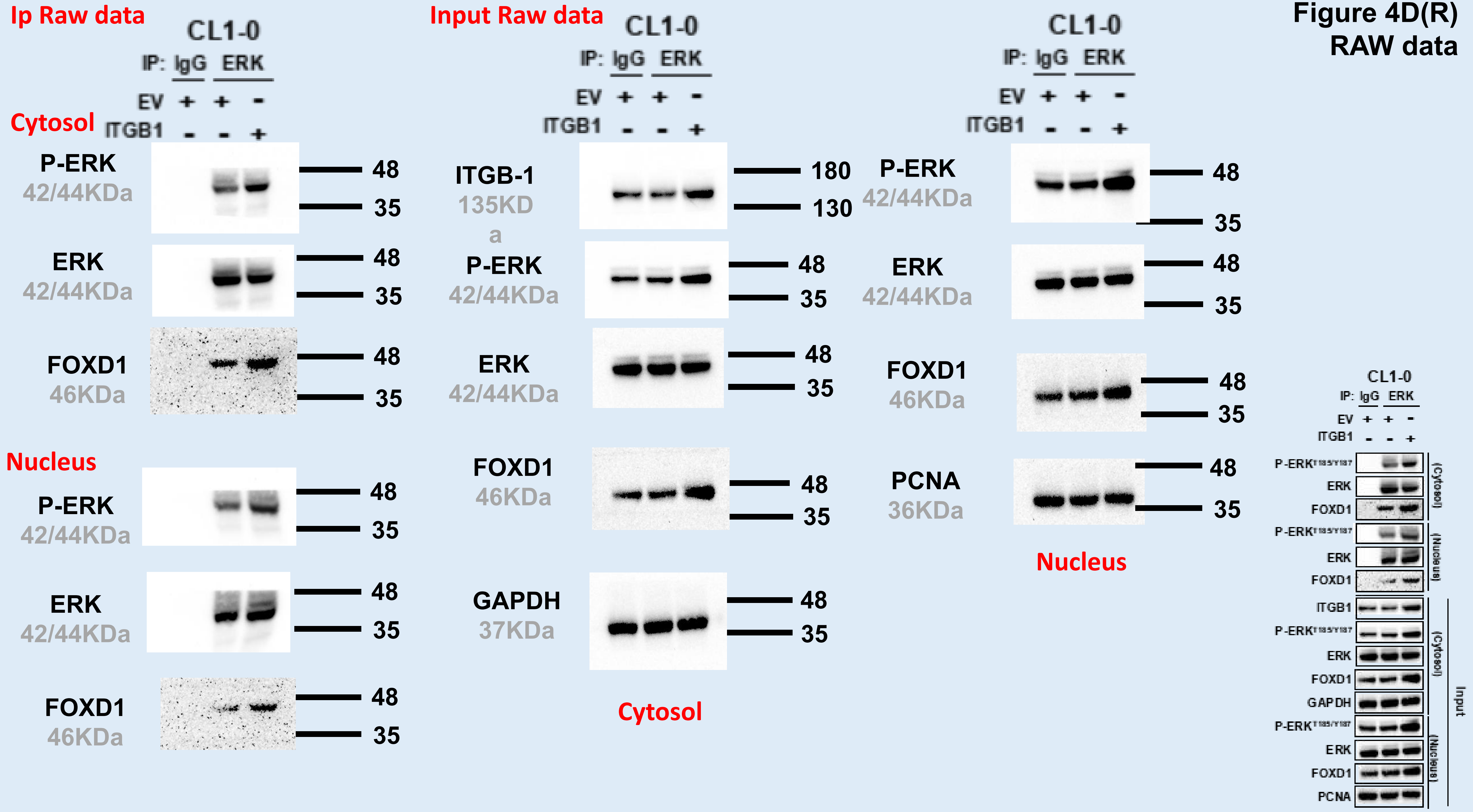

Figure 4D(R) RAW data

Figure 5A
RAW data

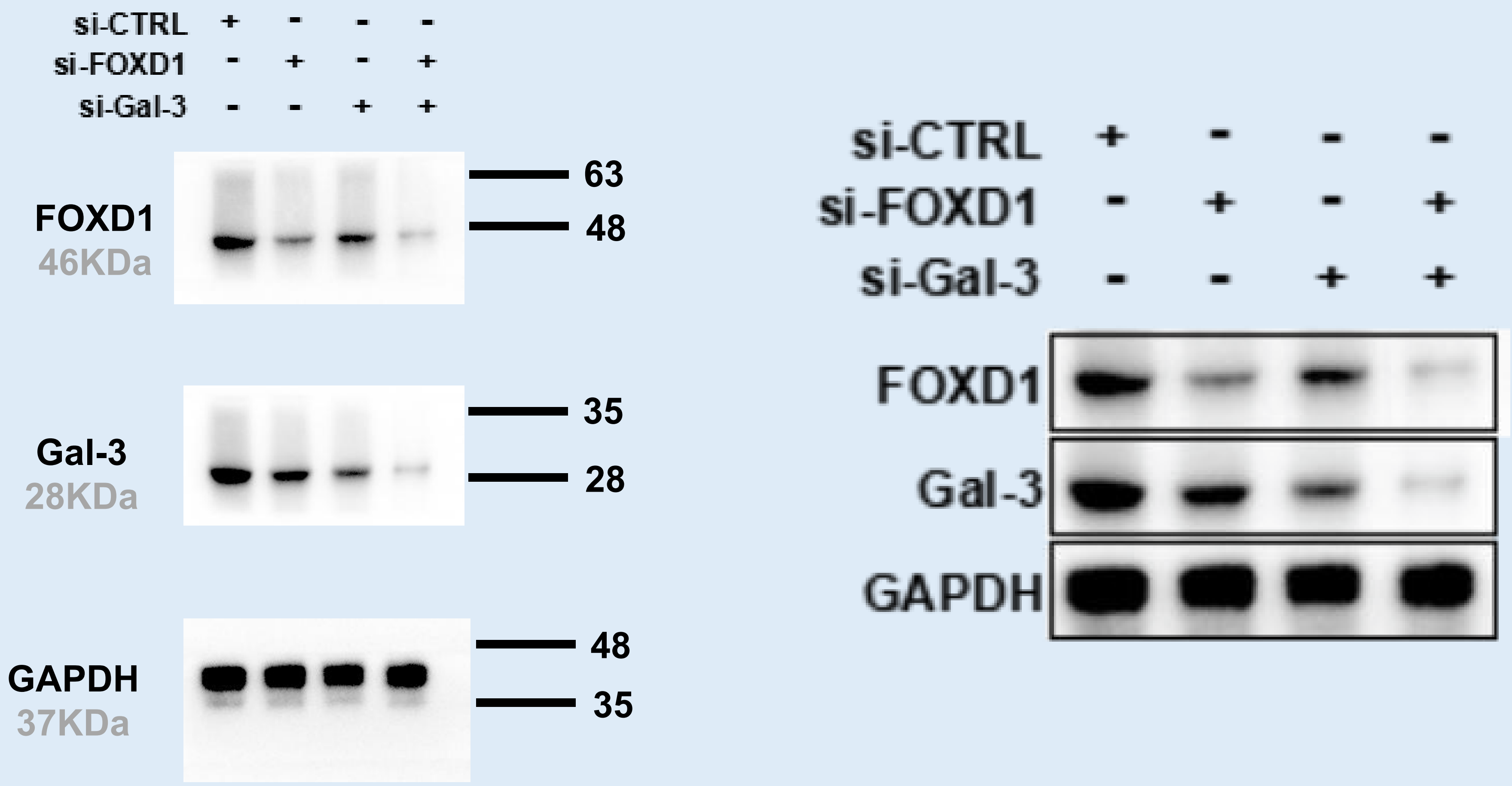

Figure 5B
RAW data

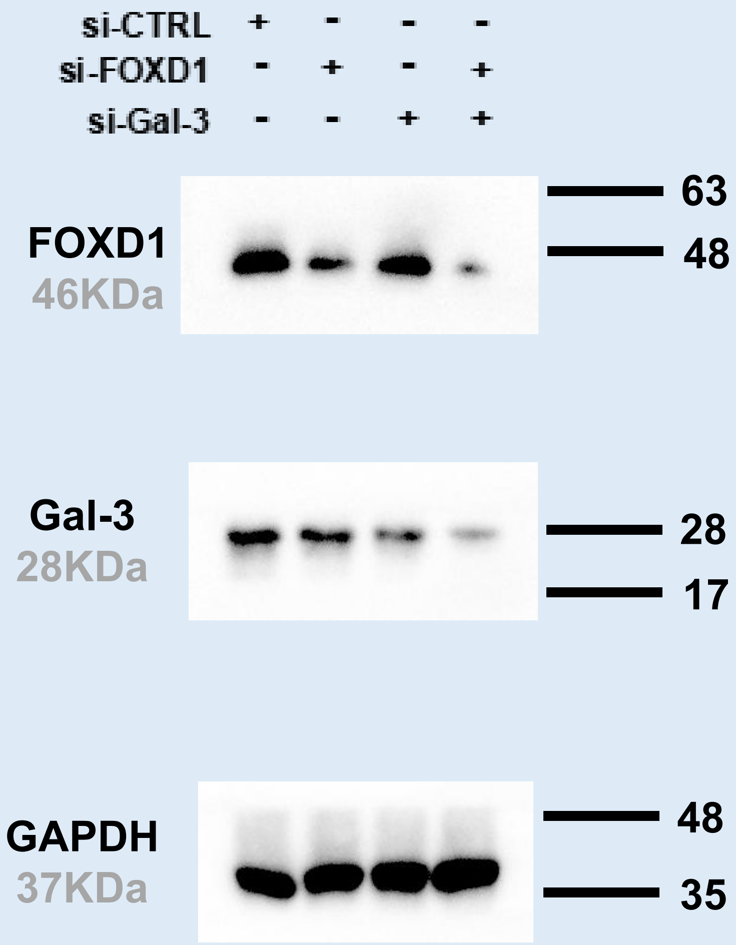

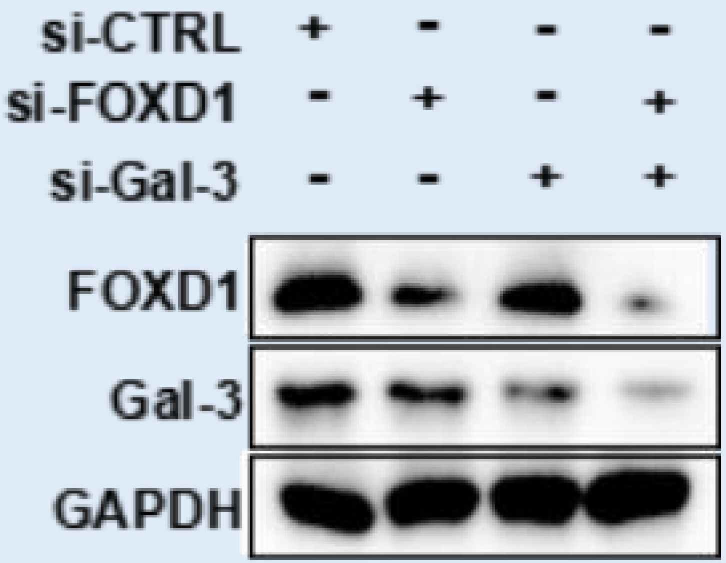

Figure 5C
RAW data

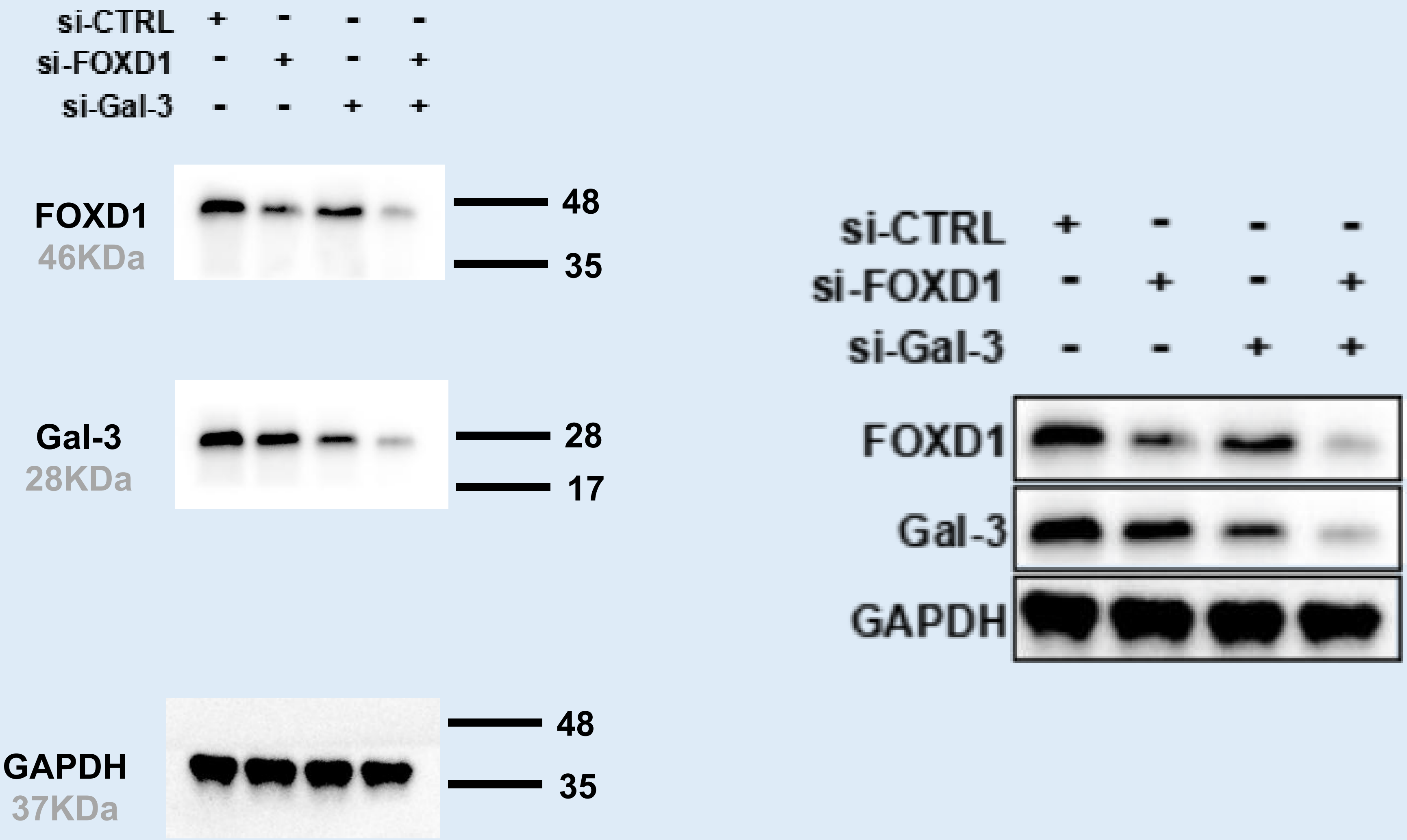

Figure 5D
RAW data

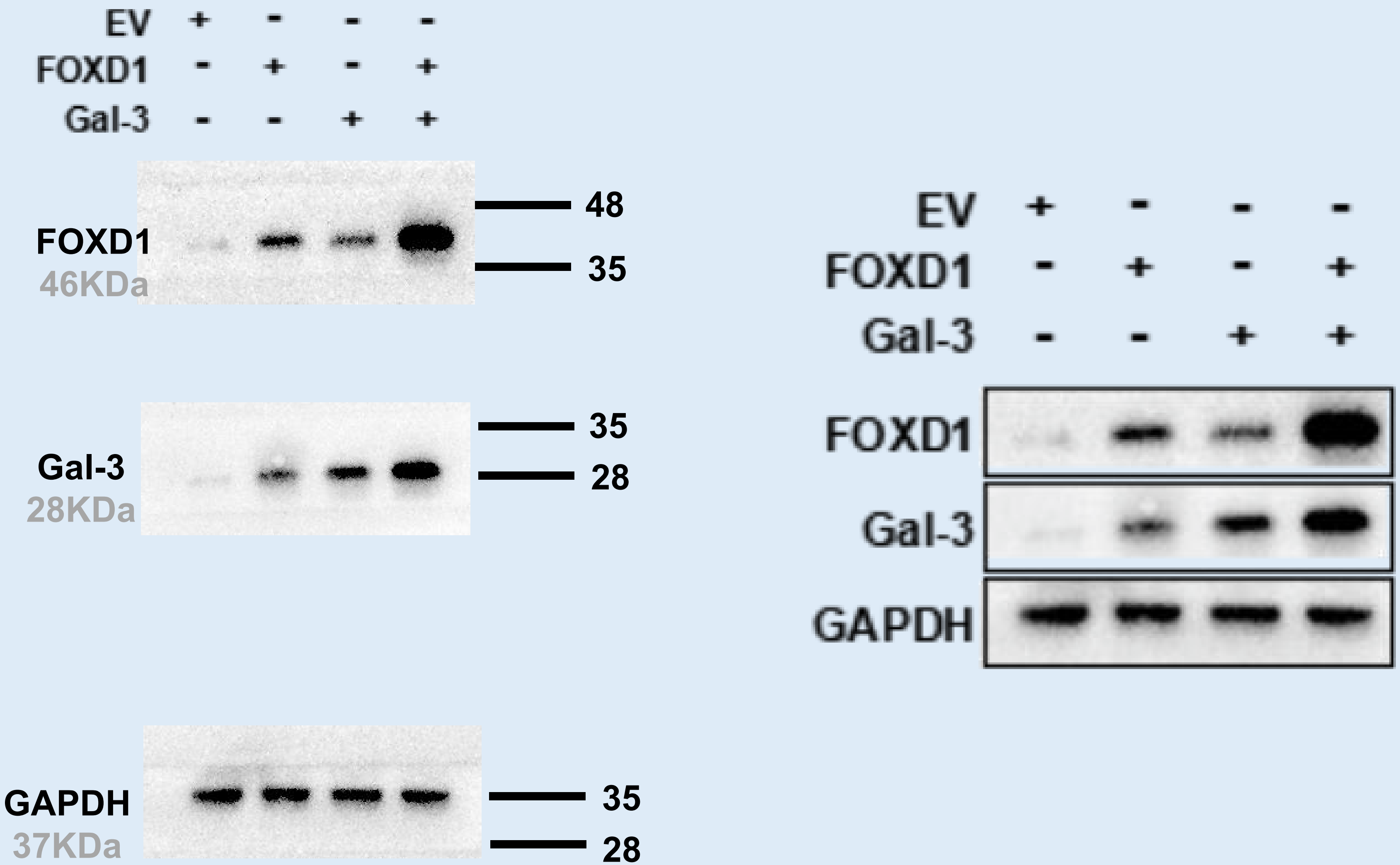

Figure 5E
RAW data

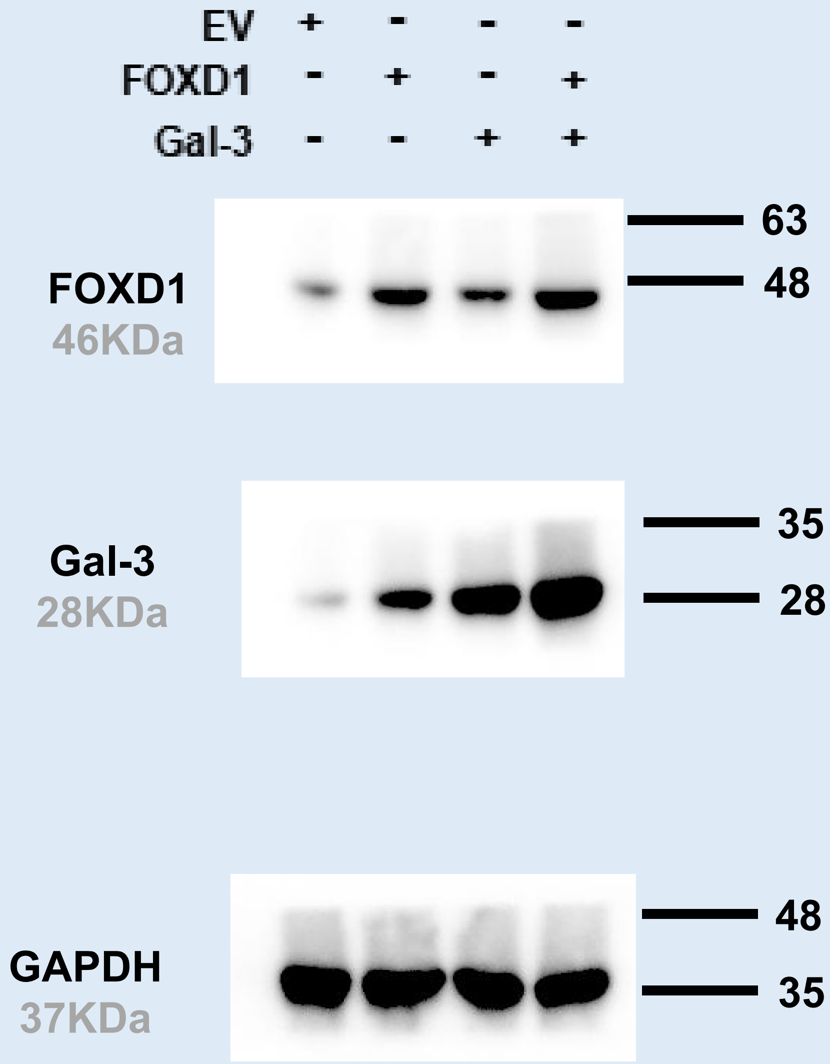

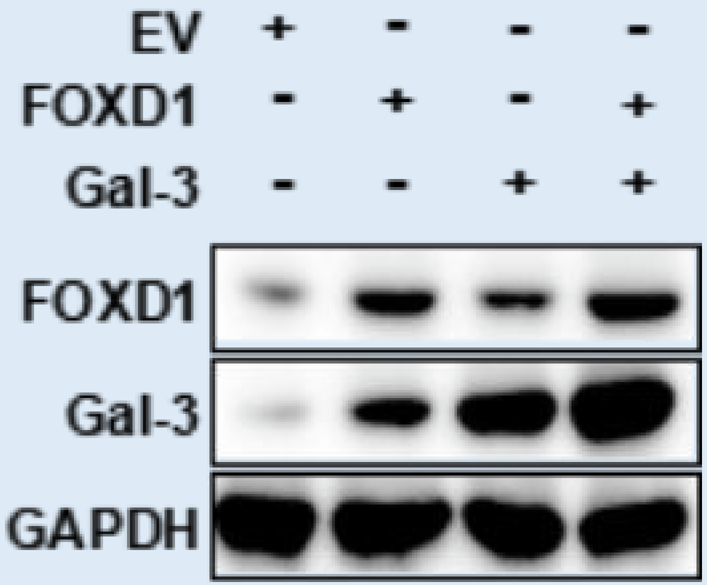

Figure 5F
RAW data

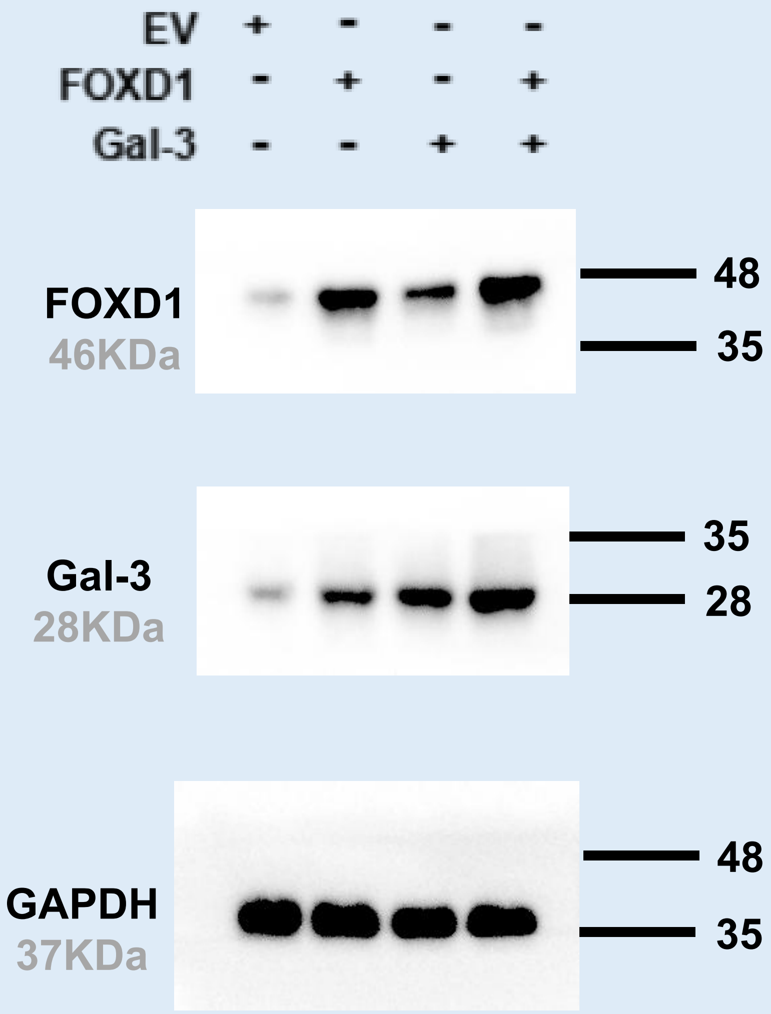

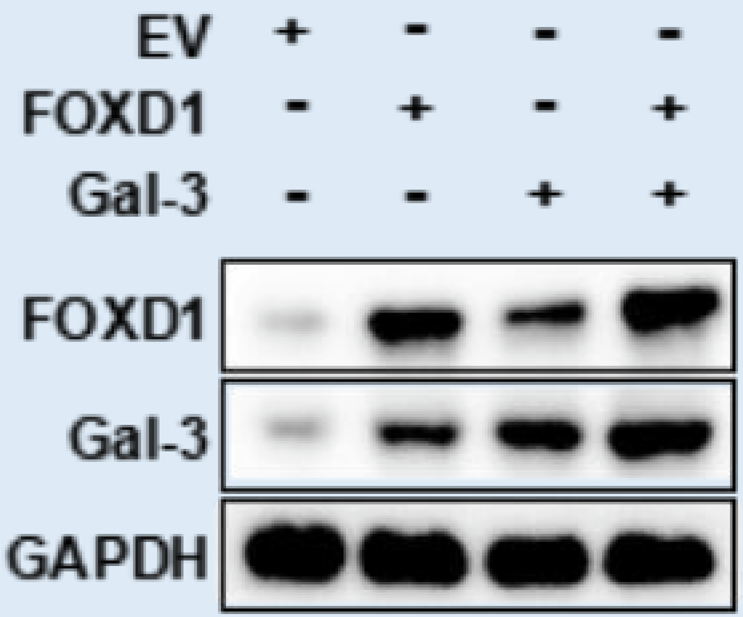

Figure S2C(L)
RAW data

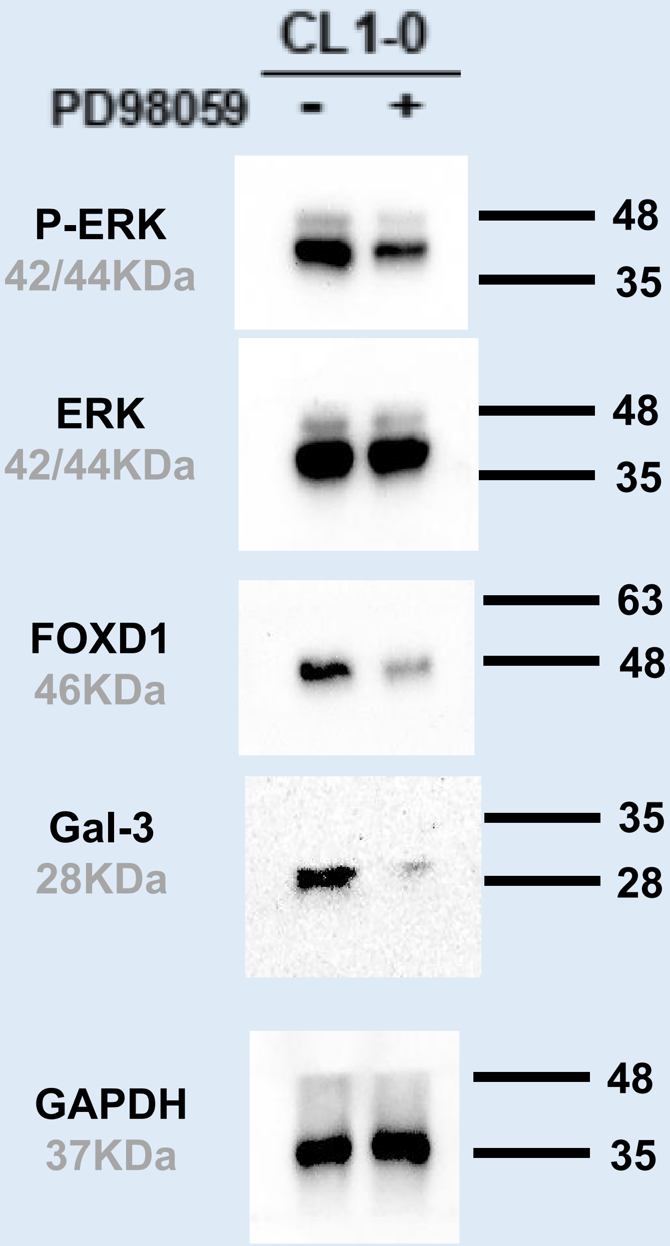

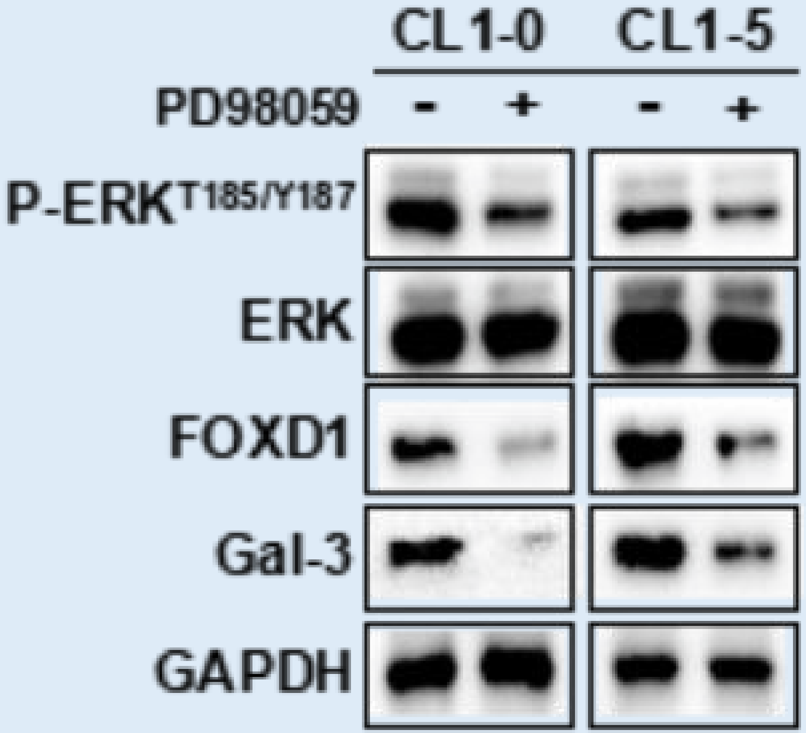

Figure S2C(R)
RAW data

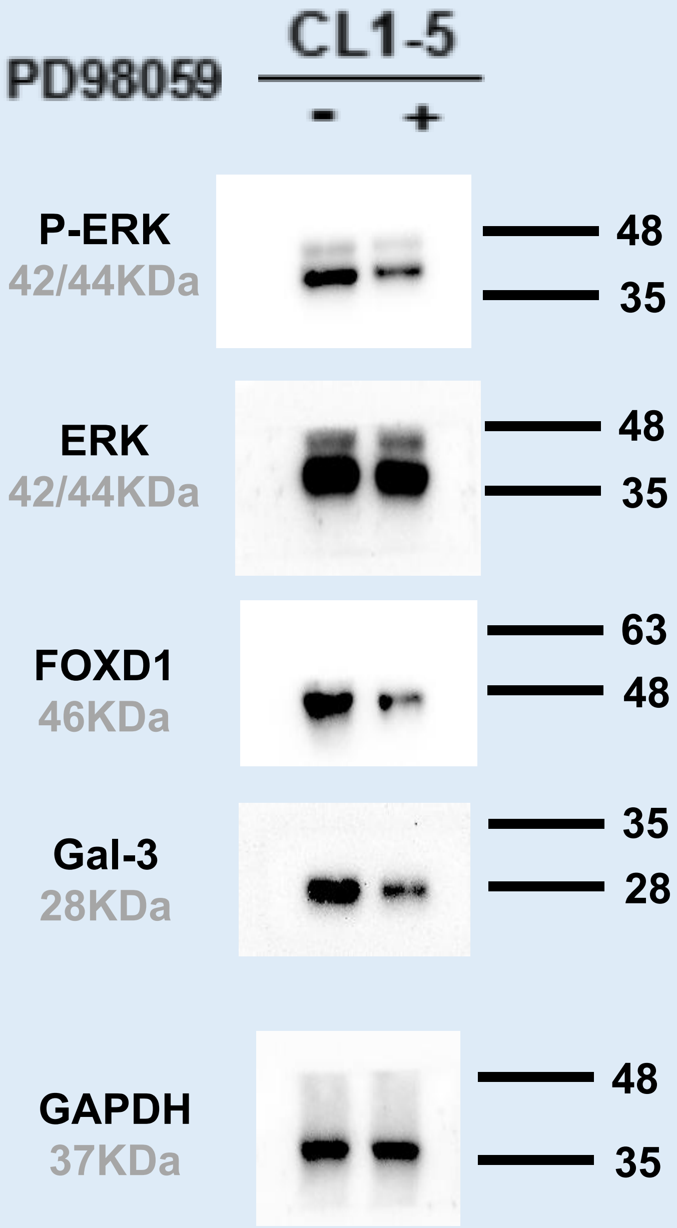

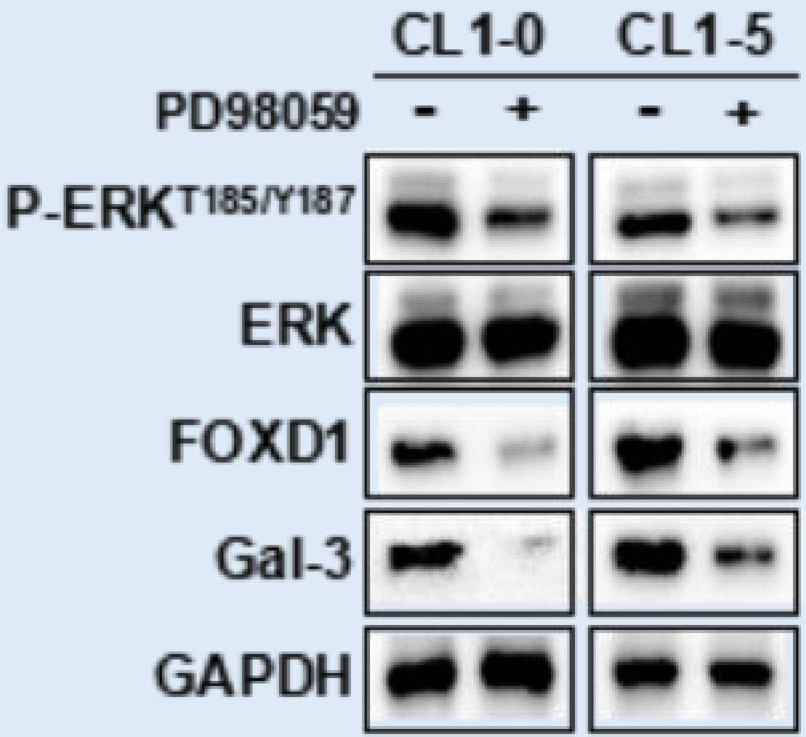

Figure S2D
RAW data

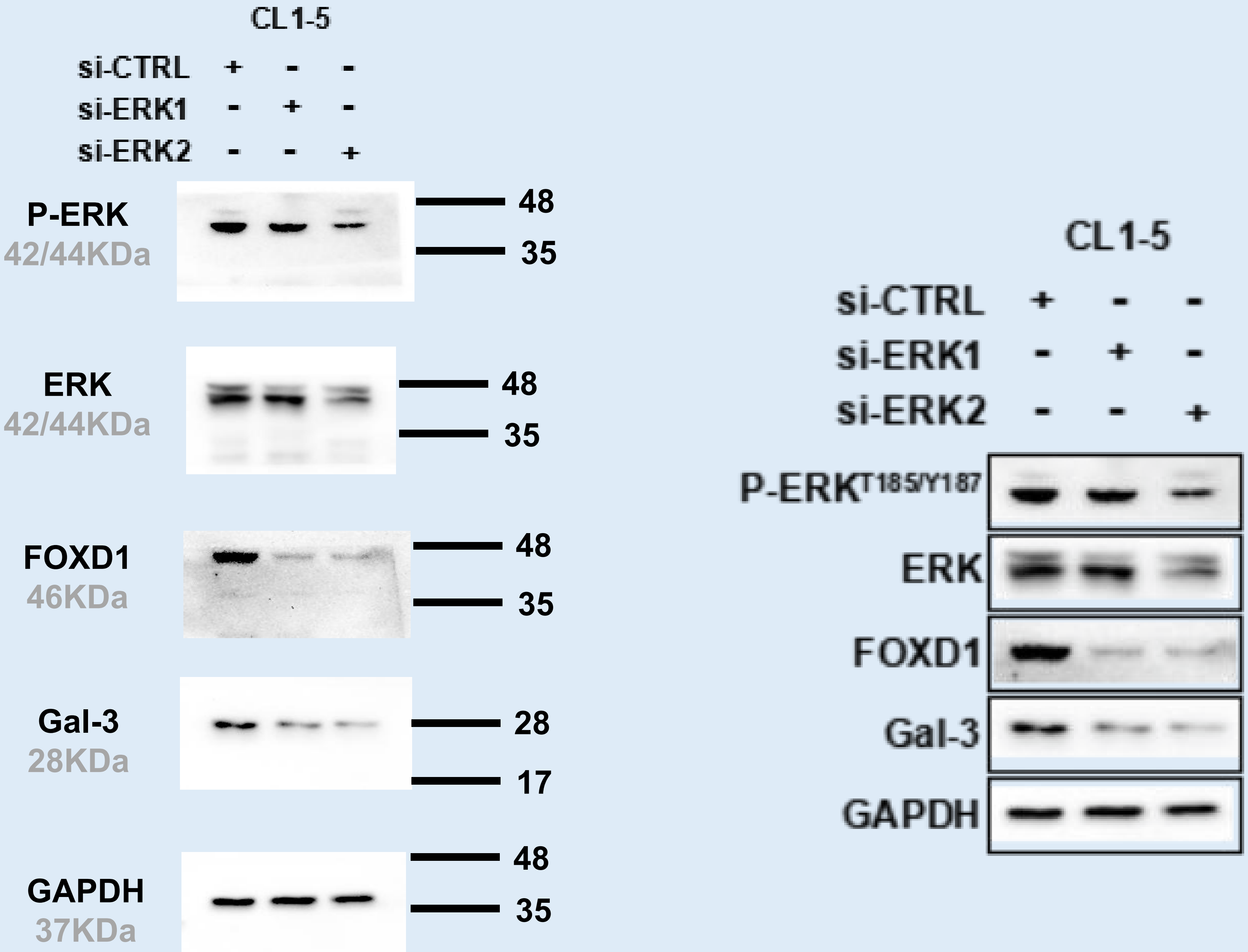

Figure S2E(L)
RAW data

Cytosol

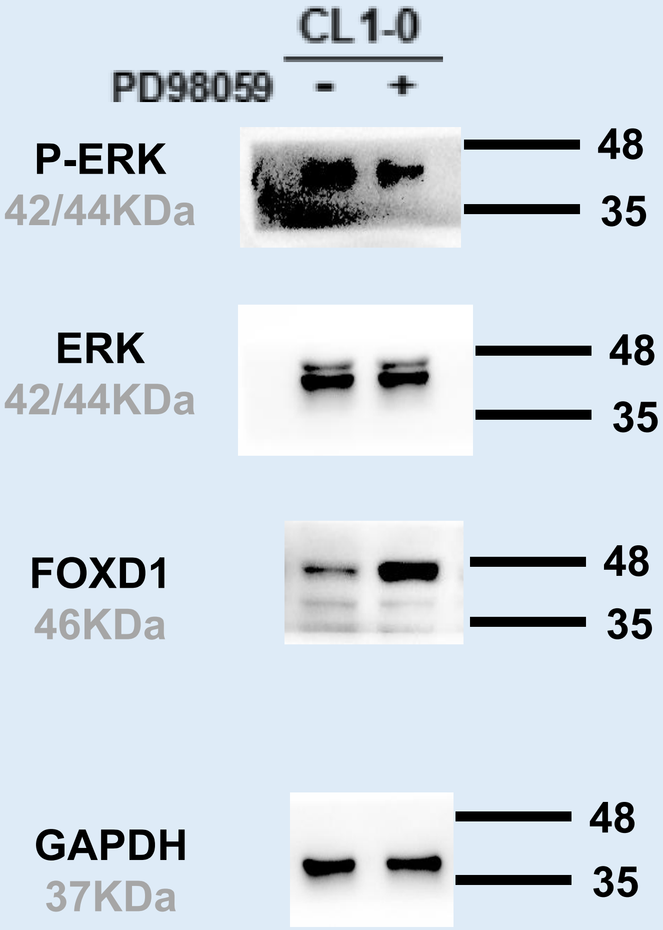

Nucleus

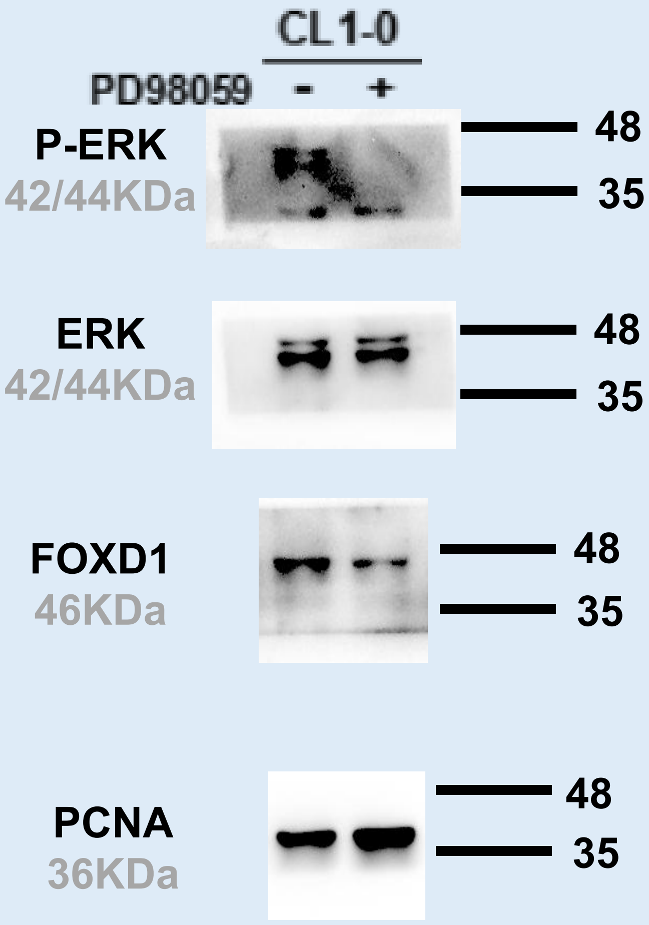

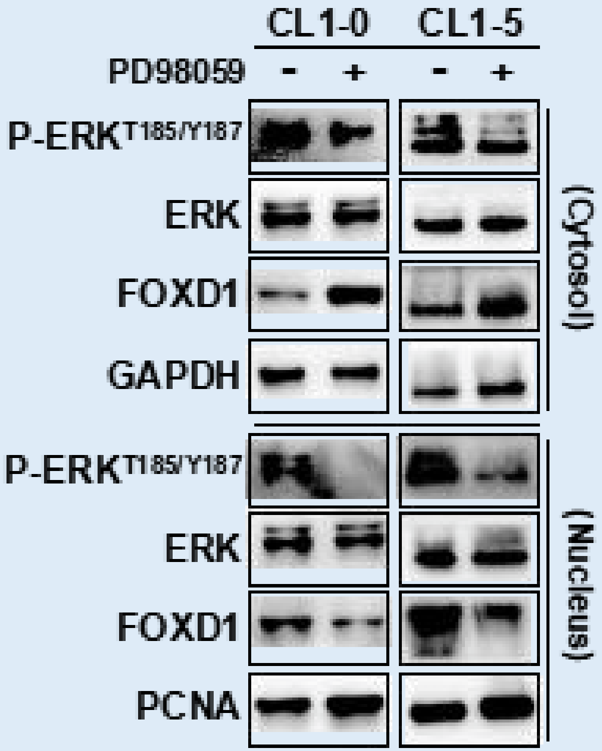

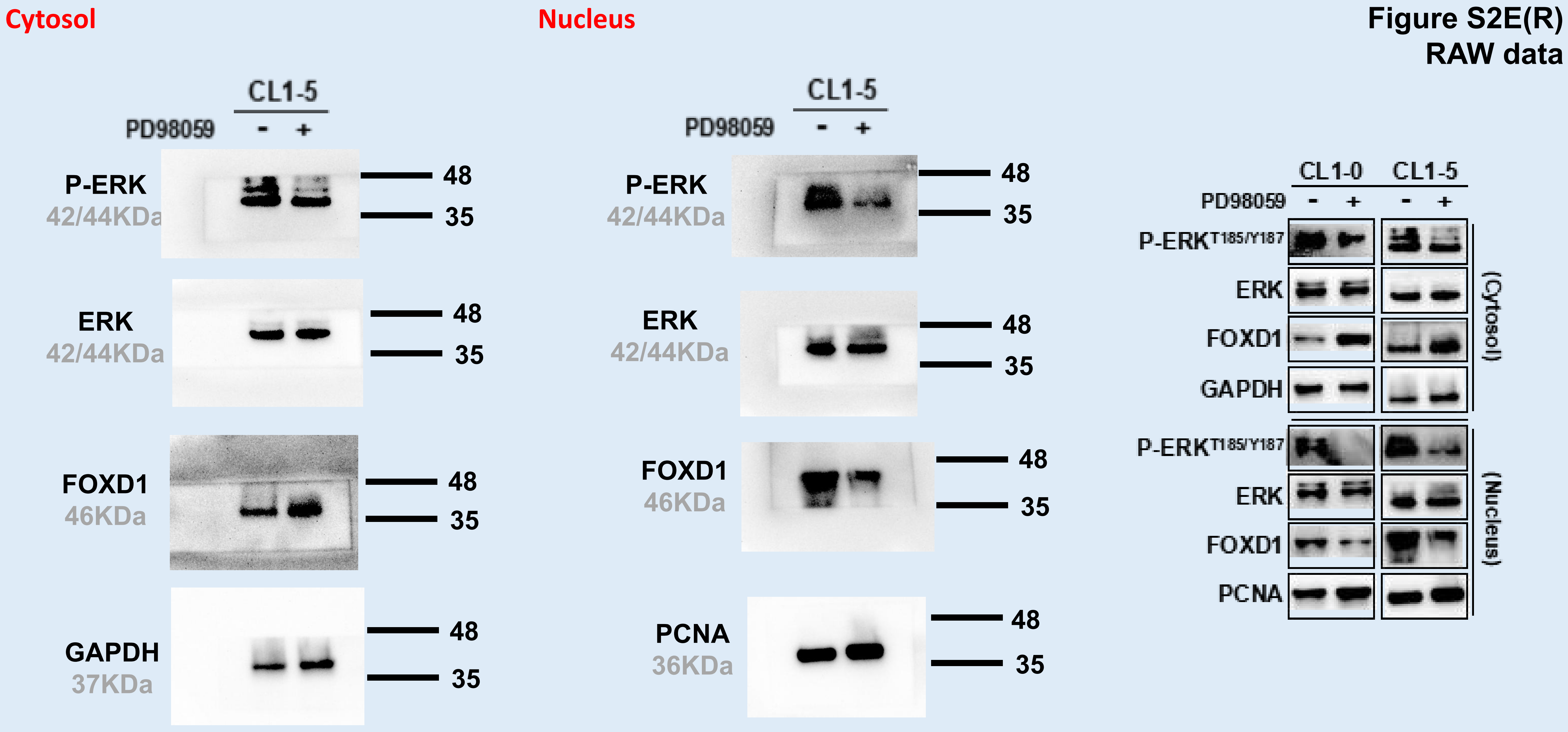
Cytosol
Nucleus
Figure S2E(R)
RAW data
CL1-5
PD98059 - +
P-ERK
42/44KDa
48
35
ERK
42/44KDa
48
35
FOXD1
46KDa
48
35
GAPDH
37KDa
48
35
CL1-5
PD98059 - +
P-ERK
42/44KDa
48
35
ERK
42/44KDa
48
35
FOXD1
46KDa
48
35
PCNA
36KDa
48
35
CL1-0 CL1-5
PD98059 - + - +
P-ERK T185/Y187
ERK
FOXD1
GAPDH
(Cytosol)
P-ERK T185/Y187
ERK
FOXD1
PCNA
(Nucleus)

Figure S2F
RAW data

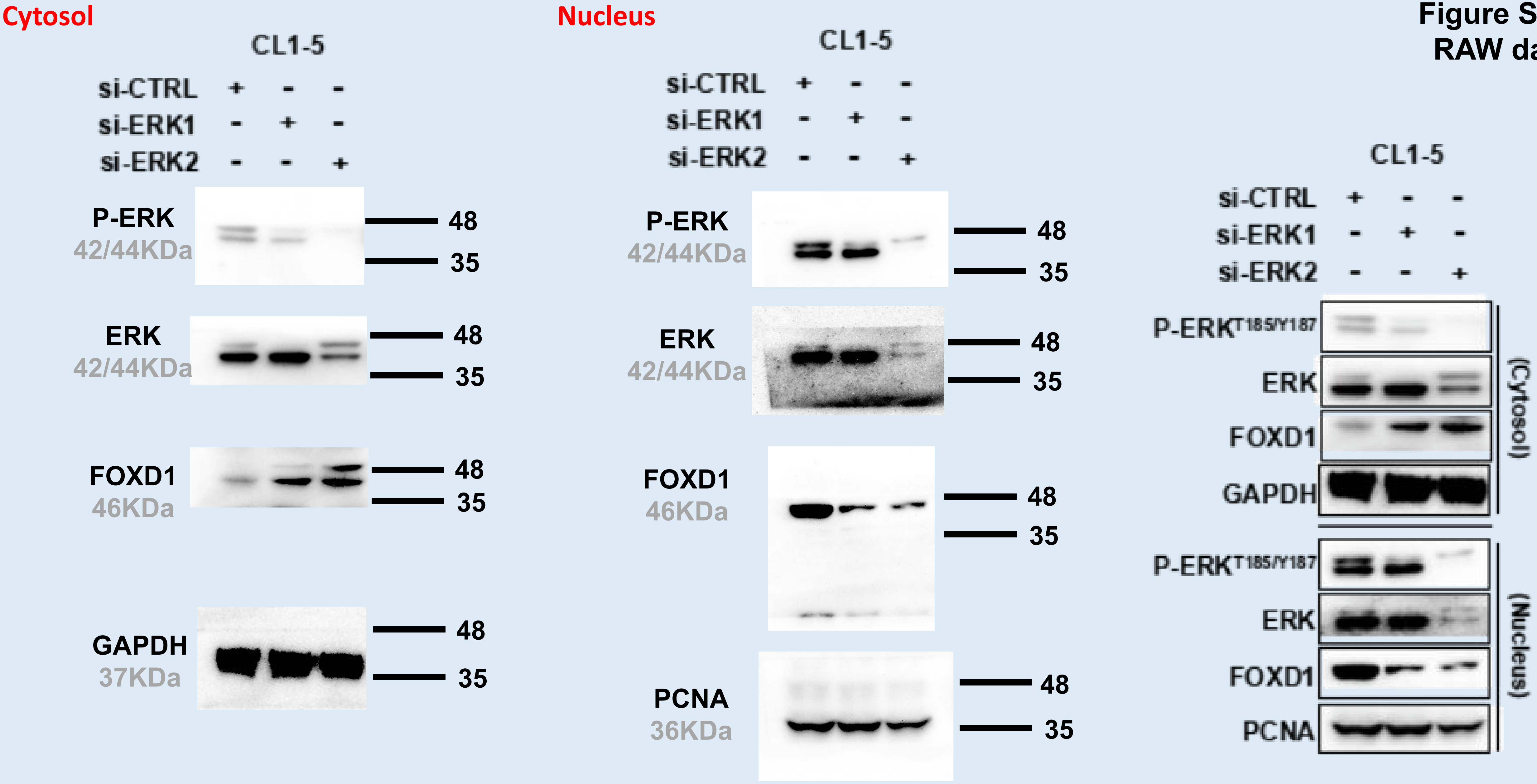

Figure S2G RAW data

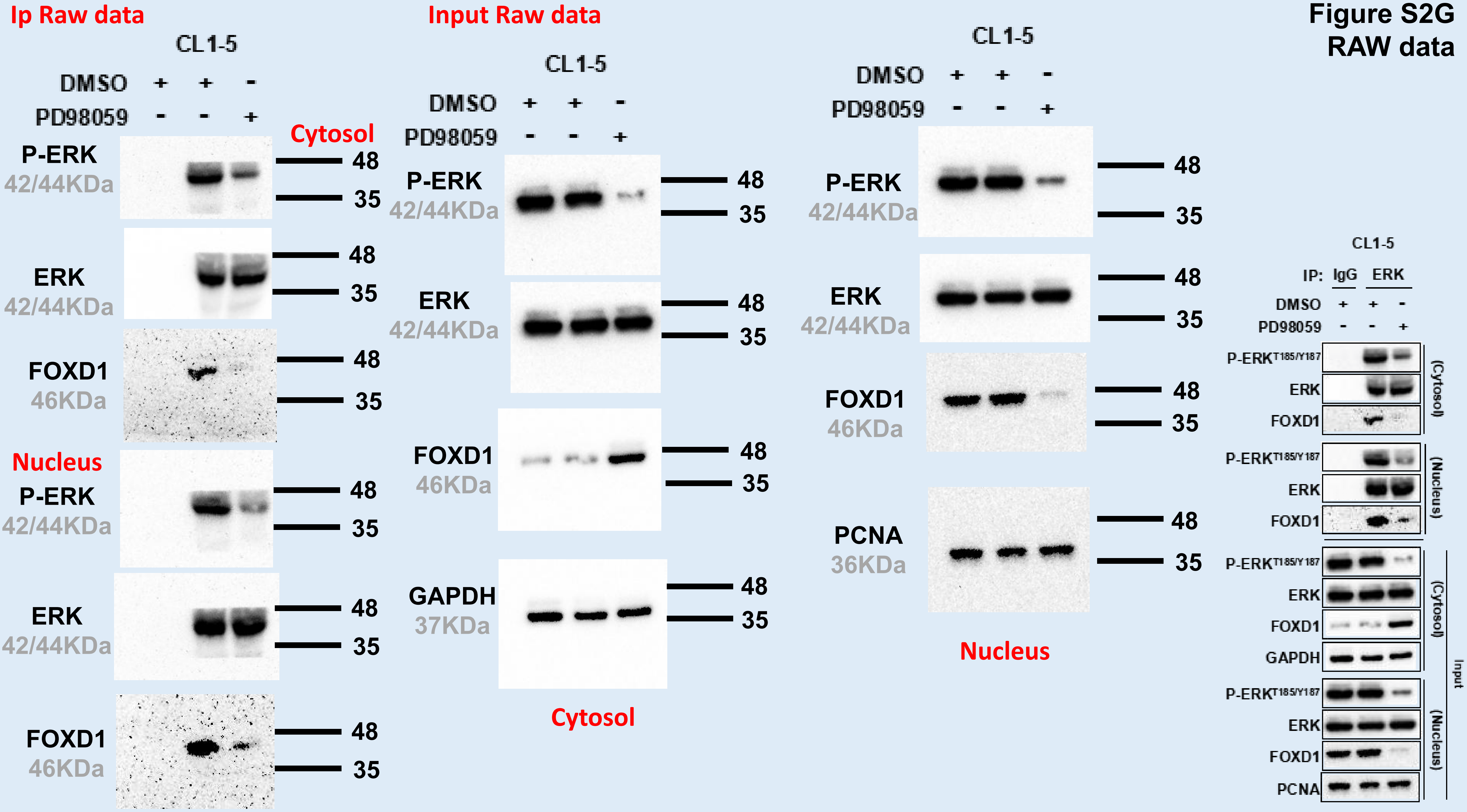

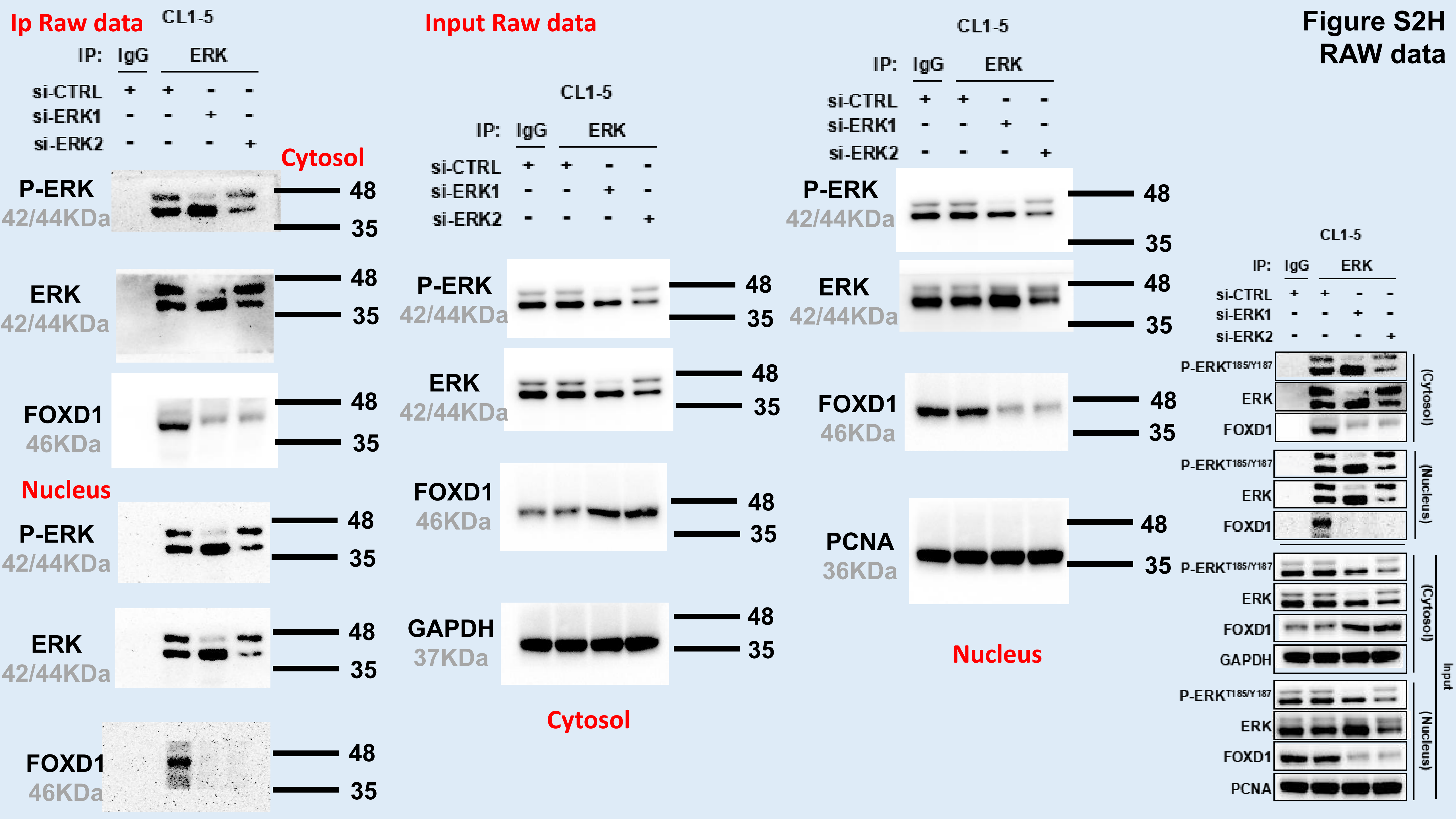
Figure S2H
RAW data
Ip Raw data
CL1-5
IP: IgG ERK
si-CTRL + + - -
si-ERK1 - - + -
si-ERK2 - - - +
Cytosol
P-ERK
42/44KDa
ERK
42/44KDa
FOXD1
46KDa
Nucleus
P-ERK
42/44KDa
ERK
42/44KDa
FOXD1
46KDa
48
35
Input Raw data
CL1-5
IP: IgG ERK
si-CTRL + + - -
si-ERK1 - - + -
si-ERK2 - - - +
P-ERK
42/44KDa
ERK
42/44KDa
FOXD1
46KDa
GAPDH
37KDa
Cytosol
CL1-5
IP: IgG ERK
si-CTRL + + - -
si-ERK1 - - + -
si-ERK2 - - - +
P-ERK
42/44KDa
ERK
42/44KDa
FOXD1
46KDa
PCNA
36KDa
Nucleus
CL1-5
IP: IgG ERK
si-CTRL + + - -
si-ERK1 - - + -
si-ERK2 - - - +
P-ERKT185/Y187
ERK
FOXD1
(Cytosol)
P-ERKT185/Y187
ERK
FOXD1
(Nucleus)
P-ERKT185/Y187
ERK
FOXD1
GAPDH
(Cytosol)
P-ERKT185/Y187
ERK
FOXD1
PCNA
(Nucleus)
Input

Figure S3C
RAW data

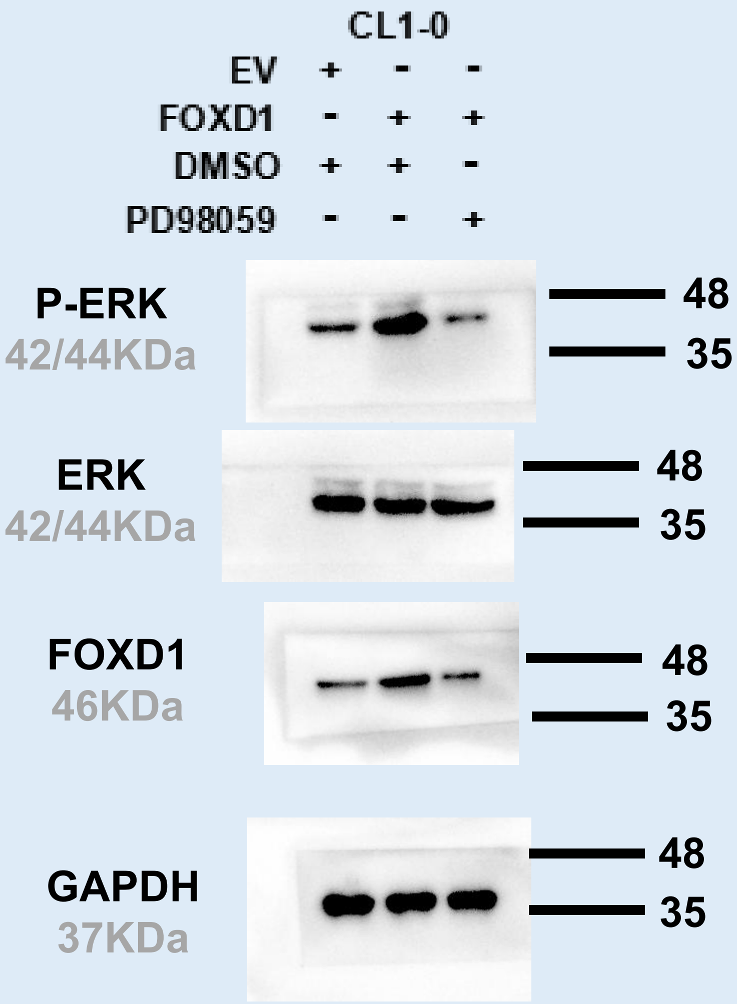

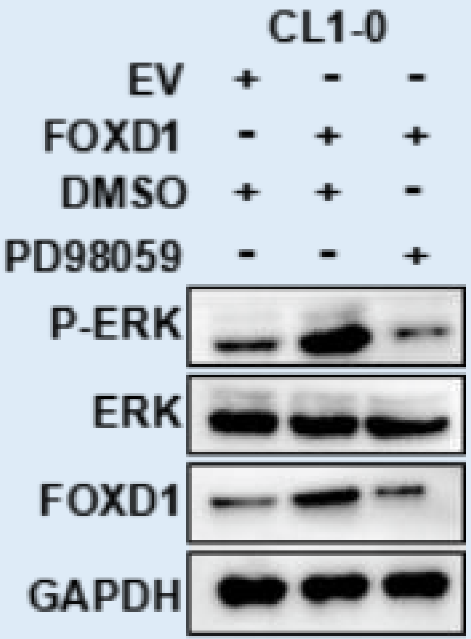

Figure S3D
RAW data

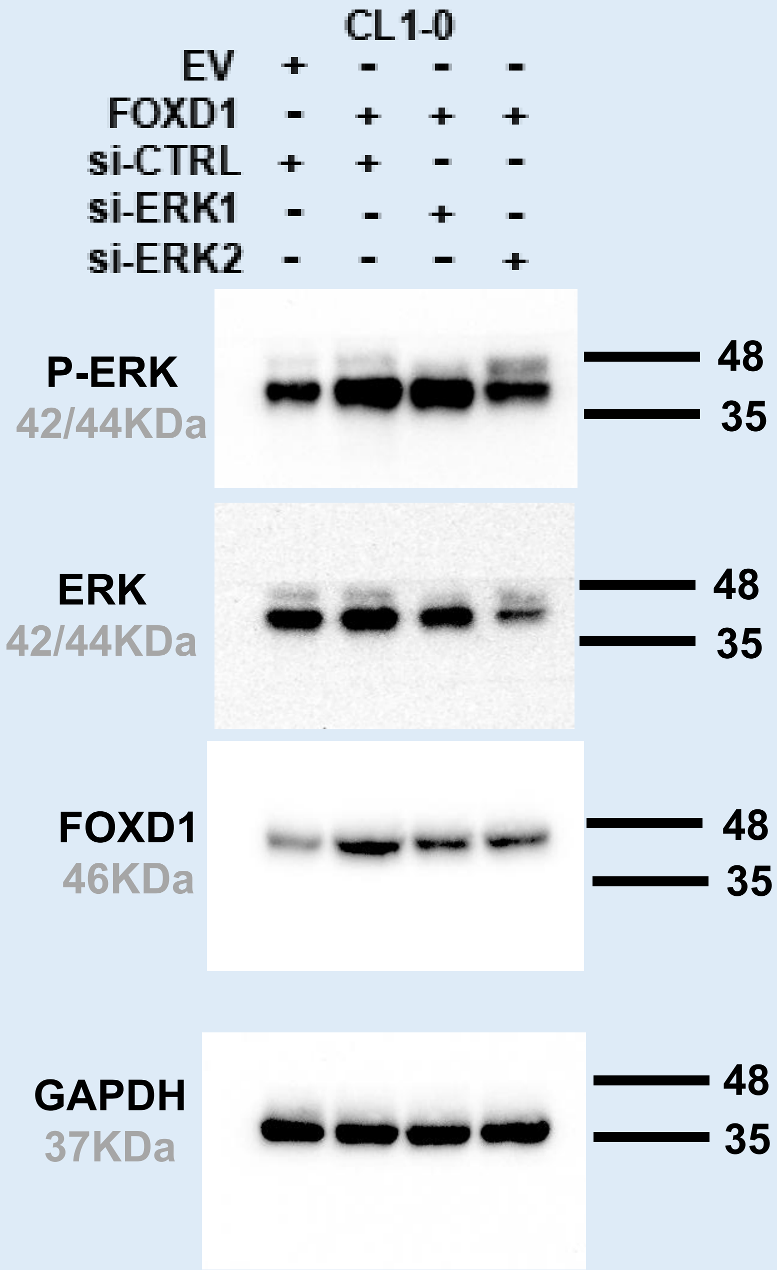

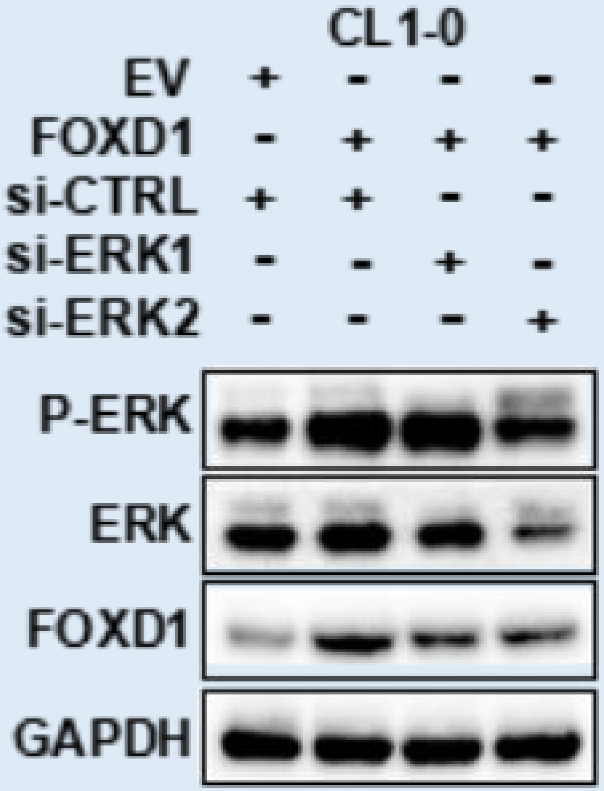

Figure S3E
RAW data

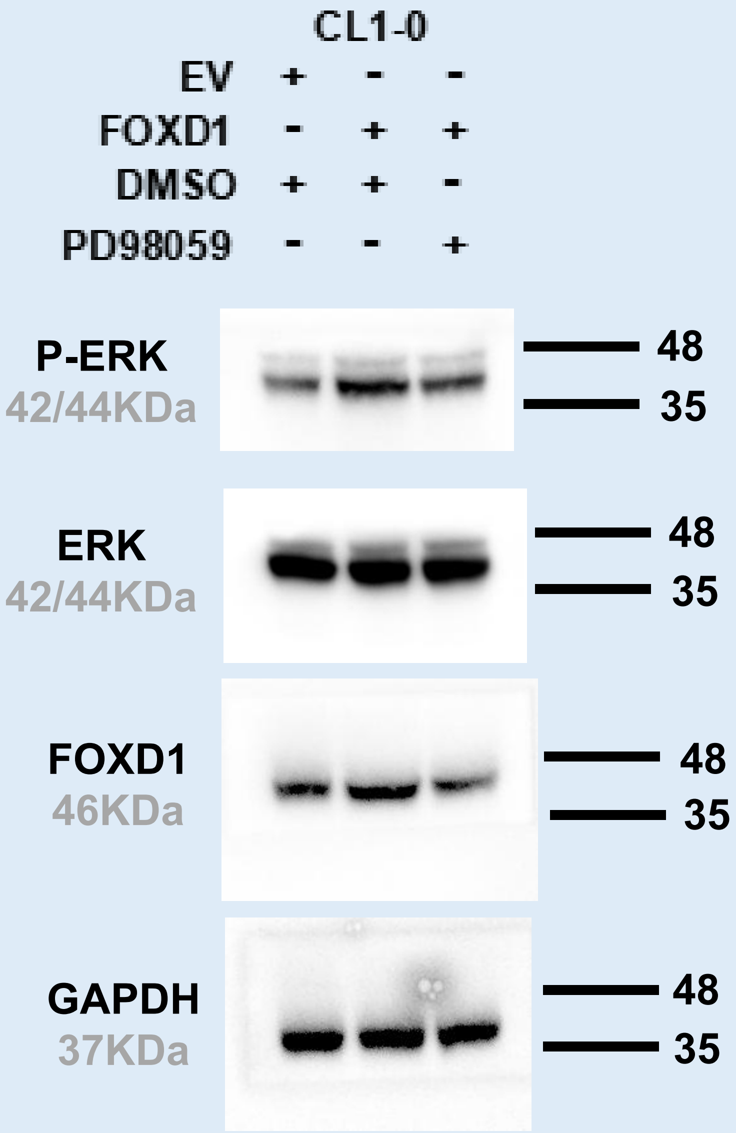

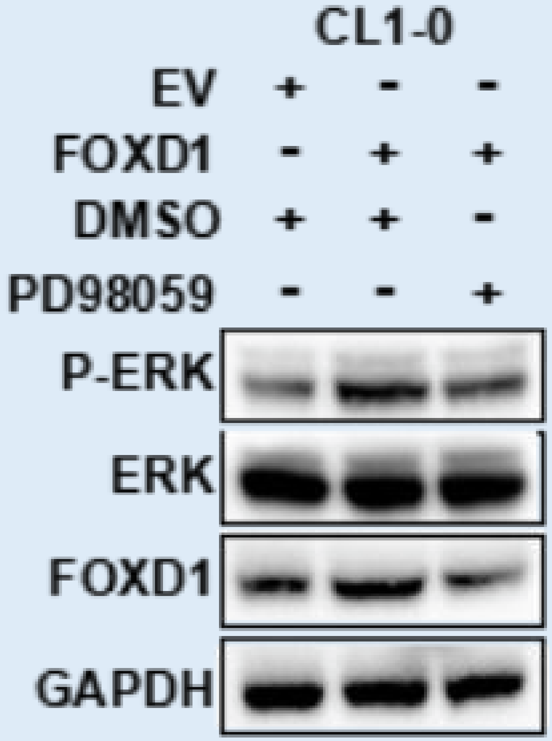

Figure S3F
RAW data

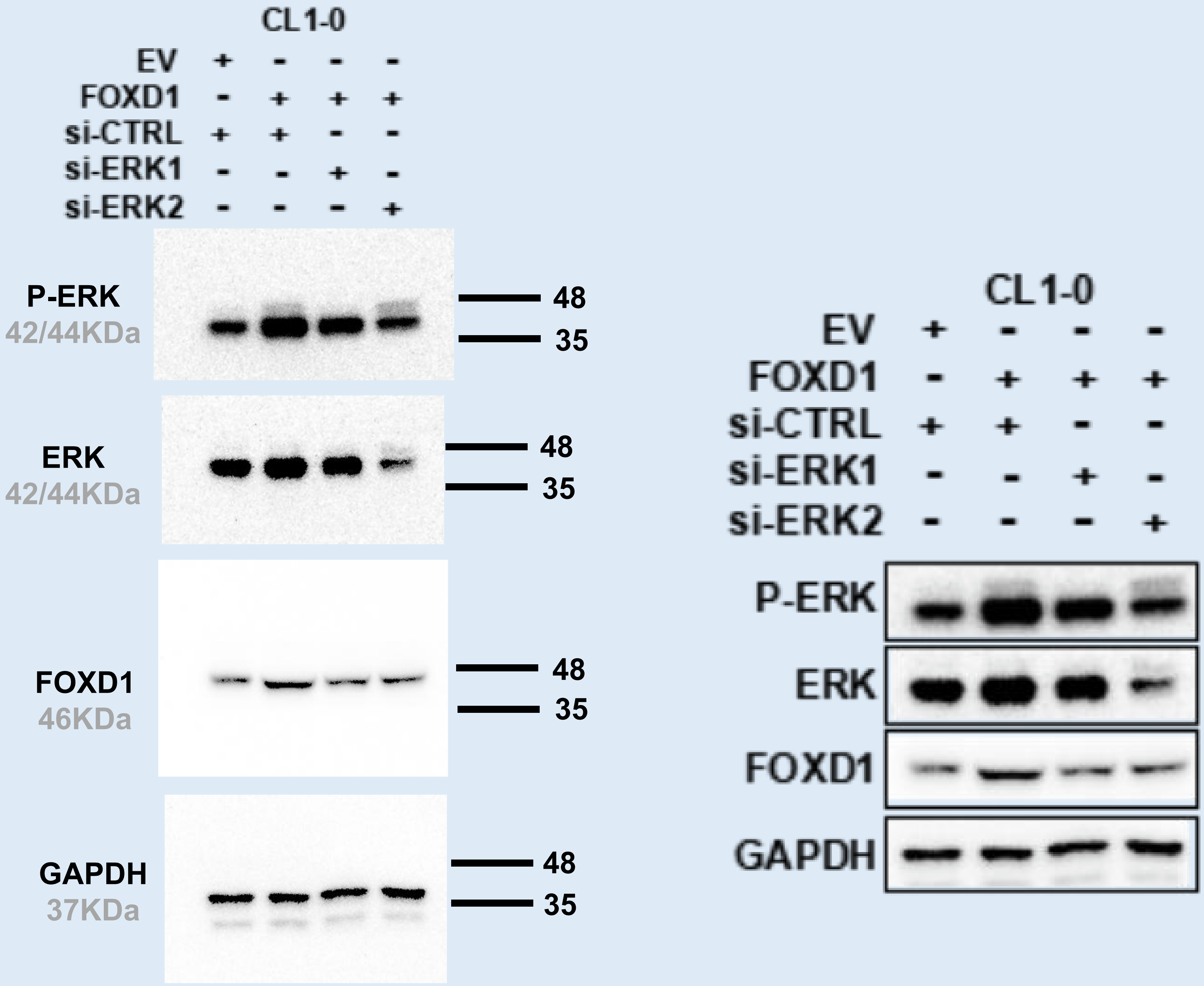

Figure S3G
RAW data

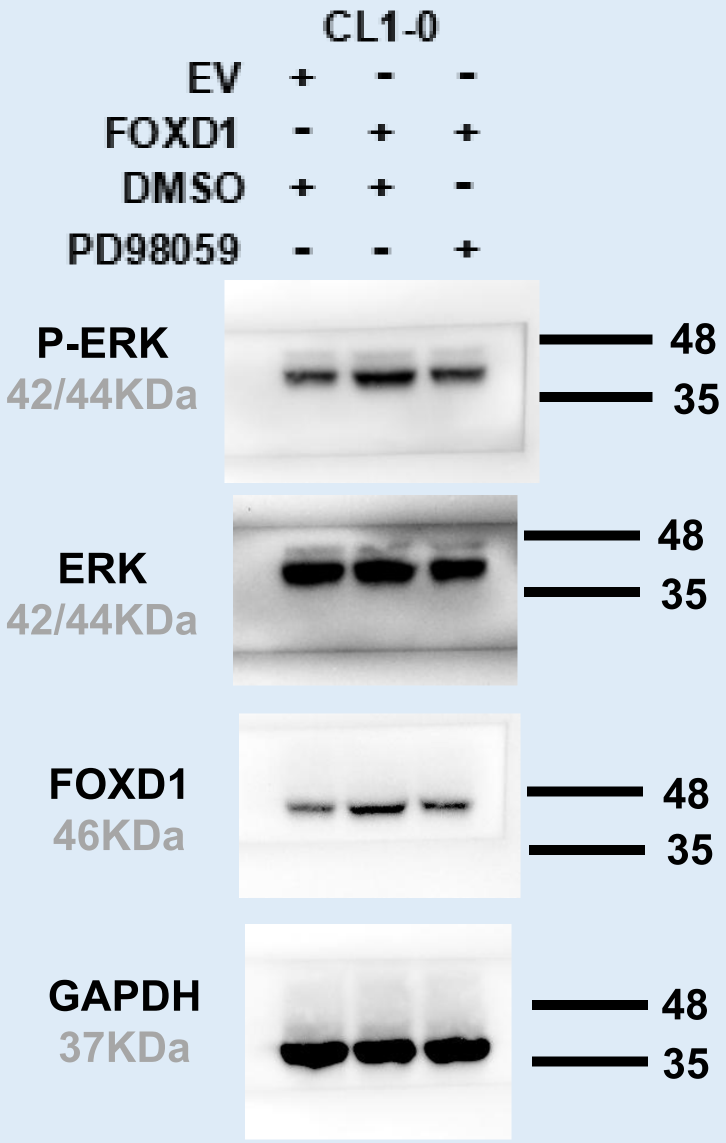

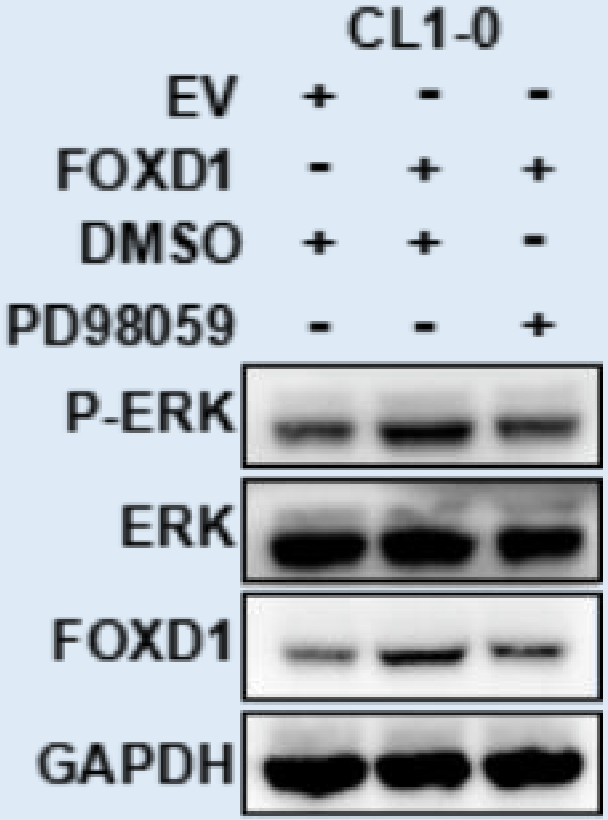

Figure S3H
RAW data

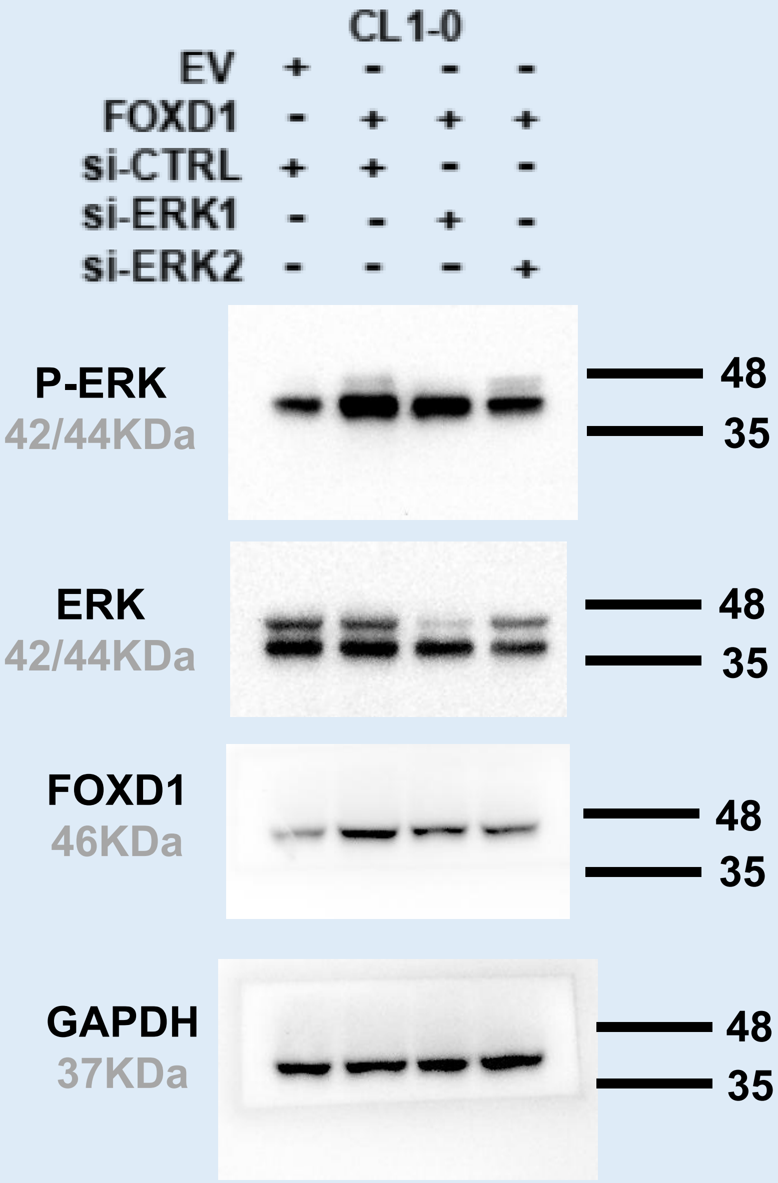

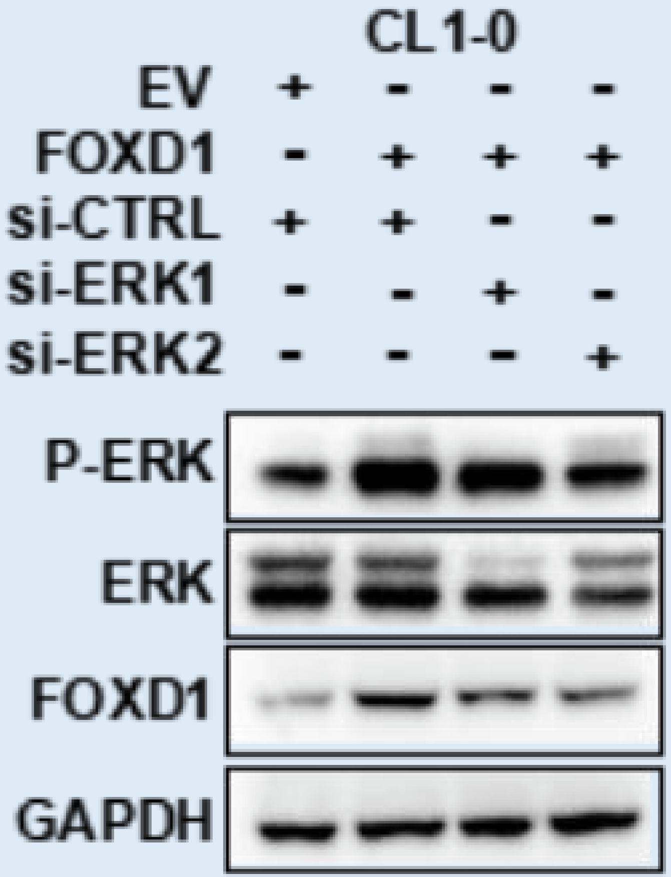

Figure S4
RAW data

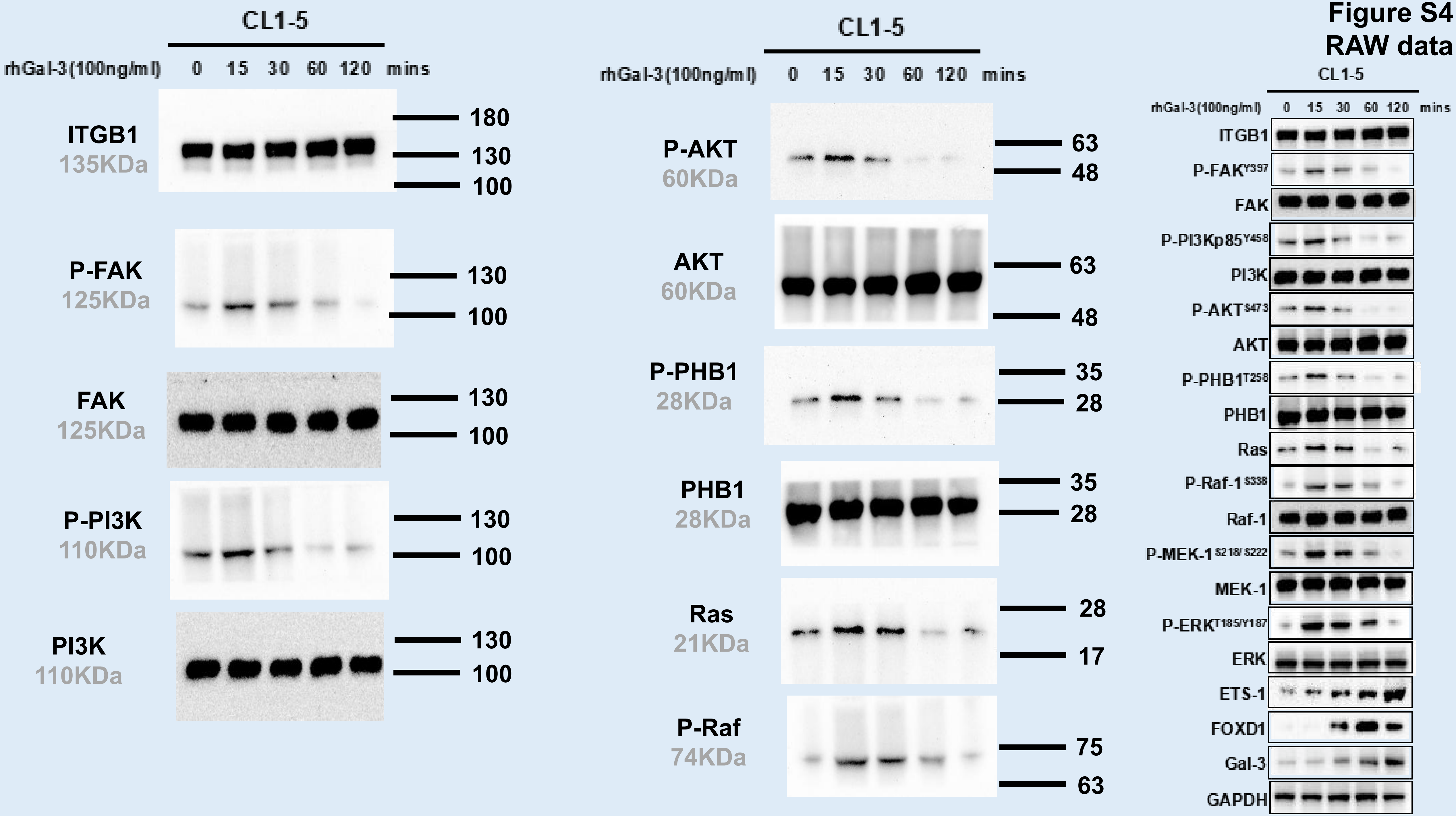

Figure S4
RAW data

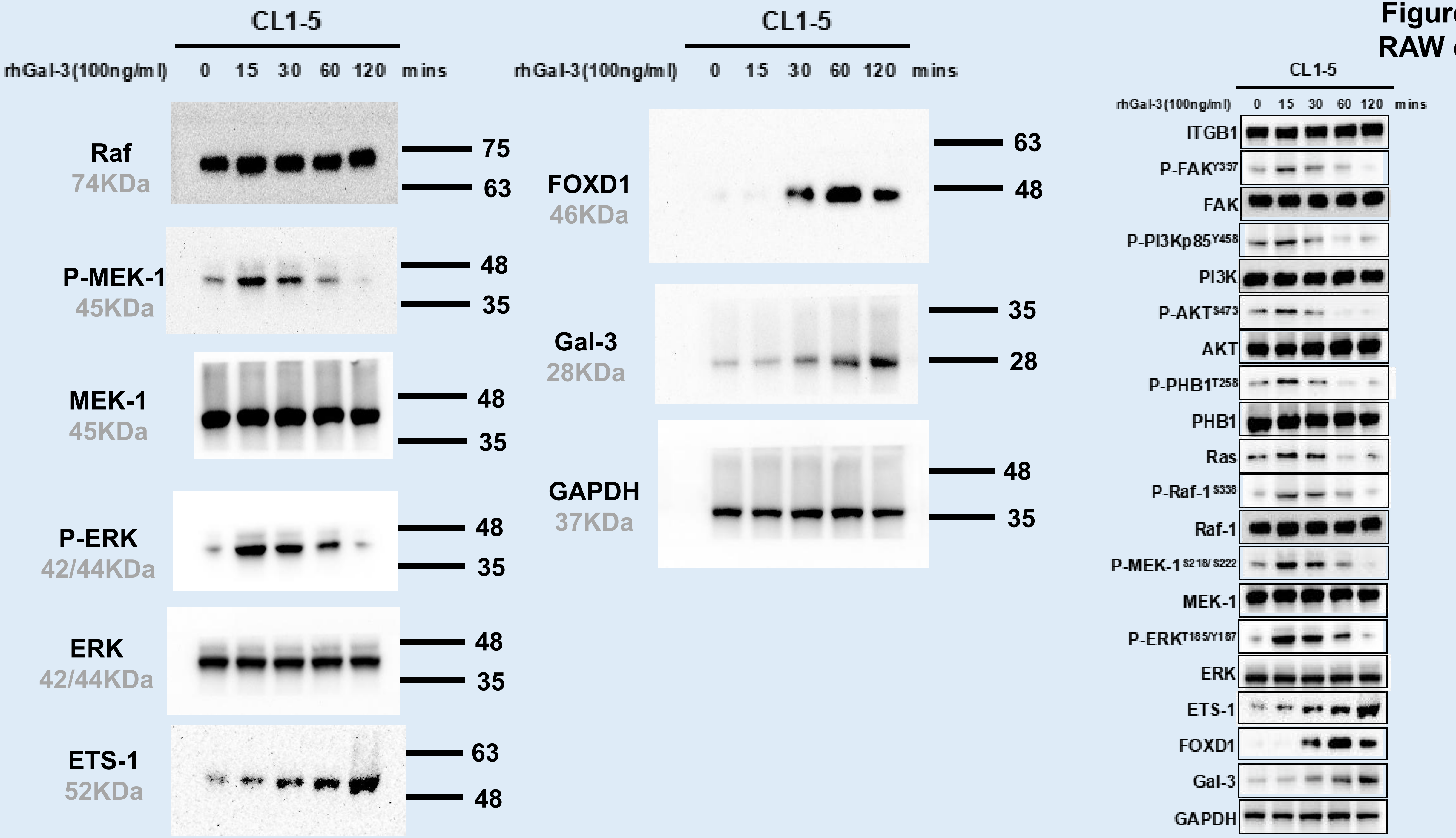

Figure S5C
RAW data

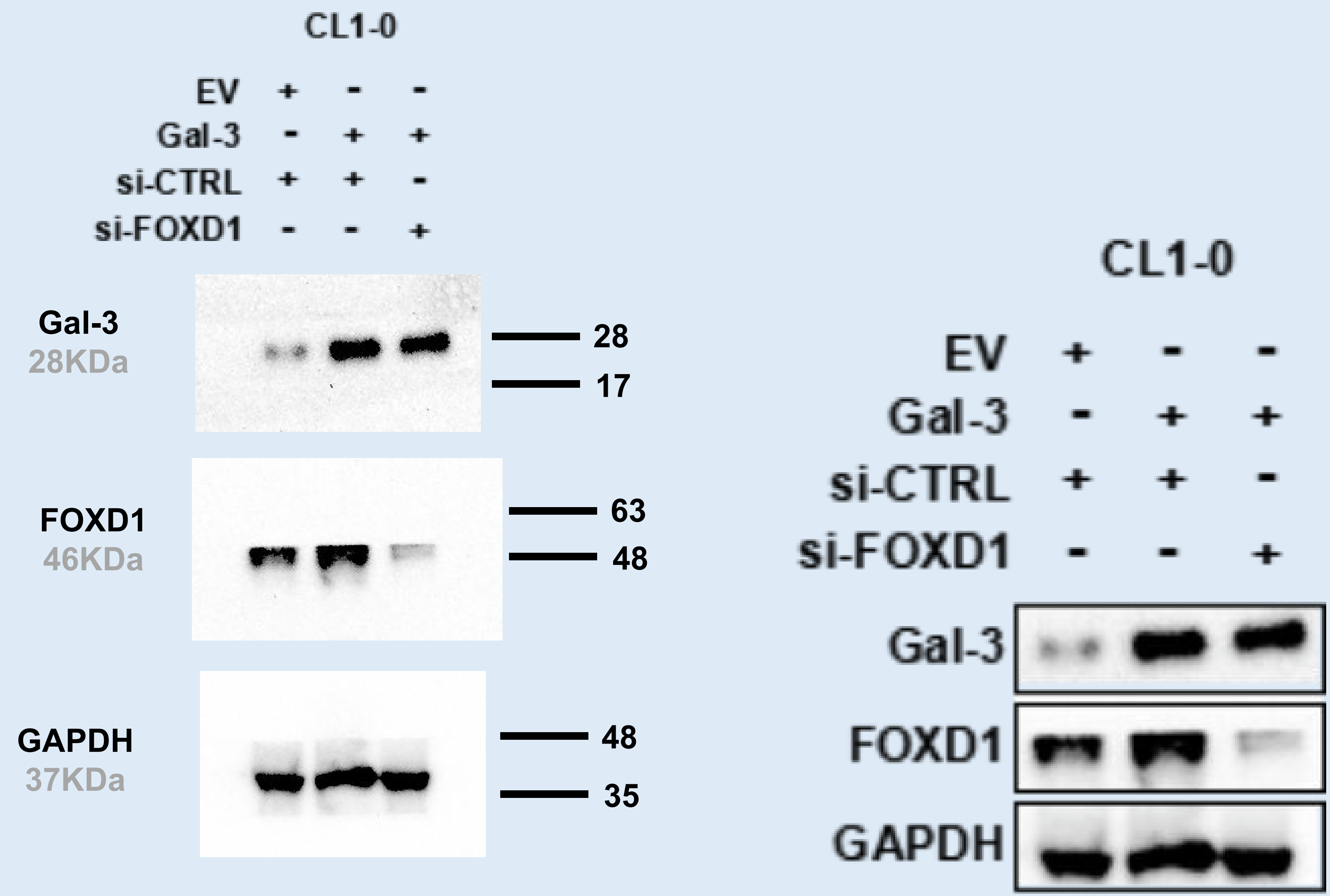

Figure S5D
RAW data

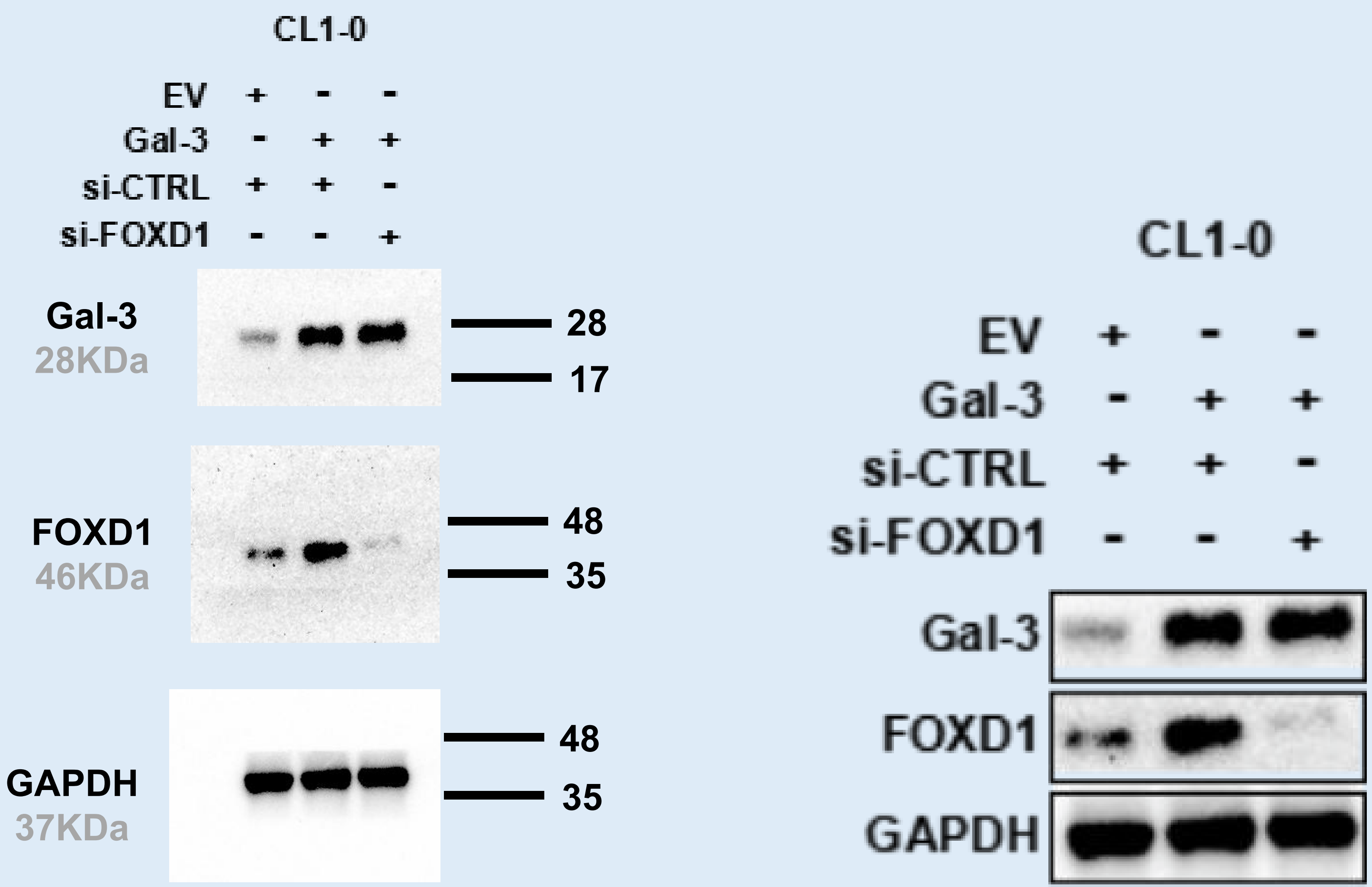

Figure S5E
RAW data

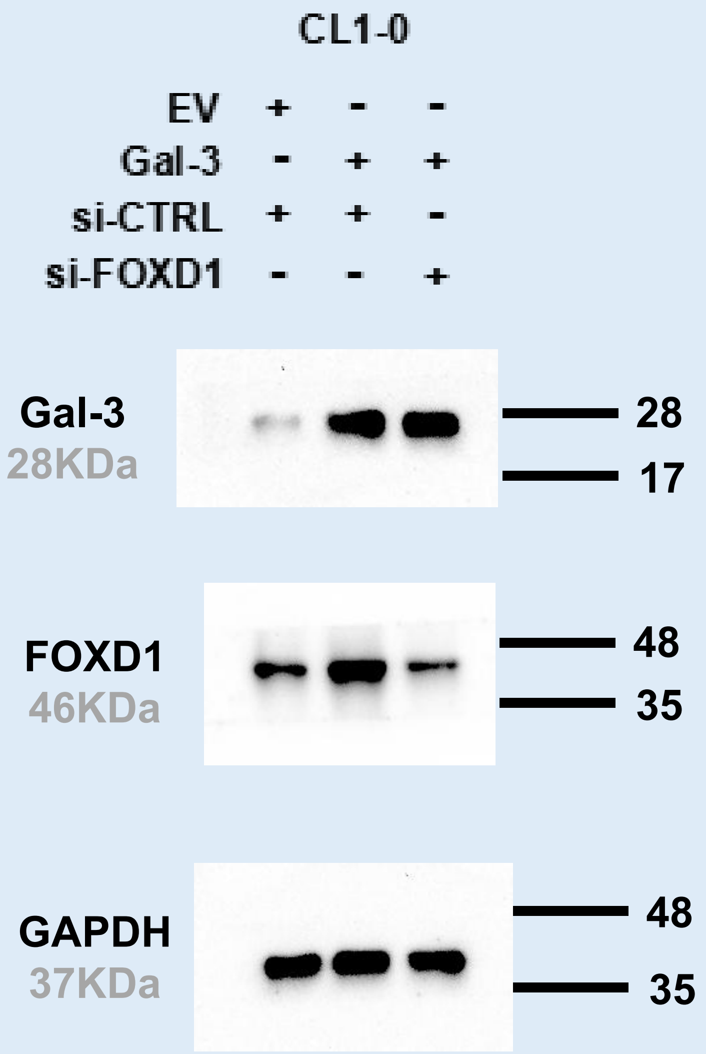

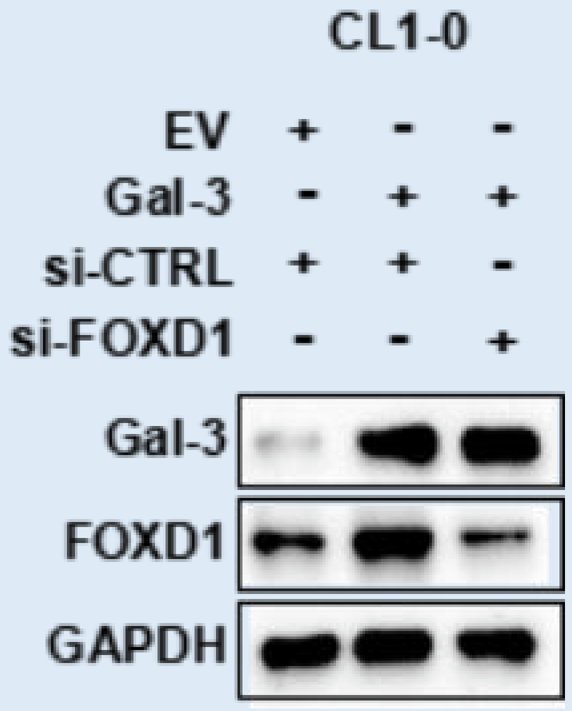

Figure S6A
RAW data

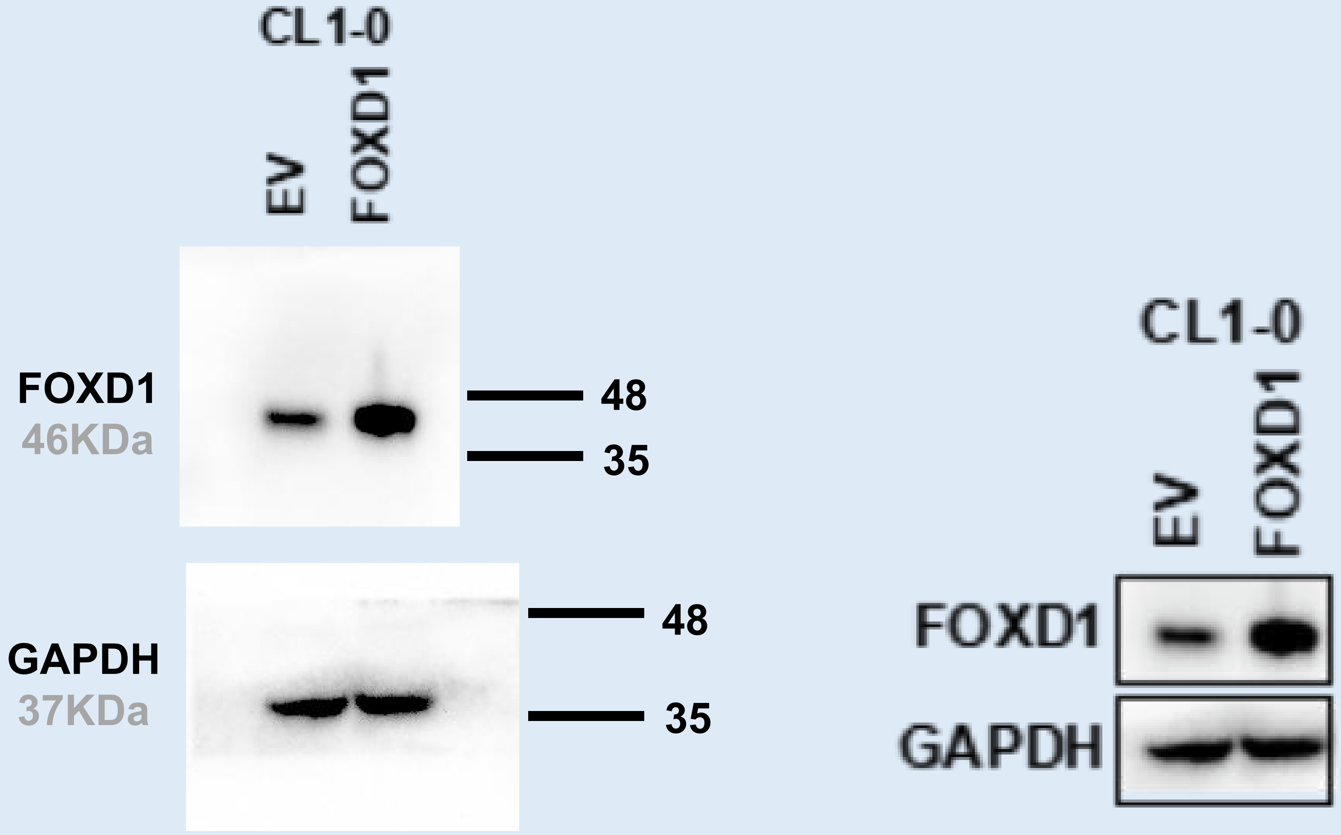

Figure S6B
RAW data

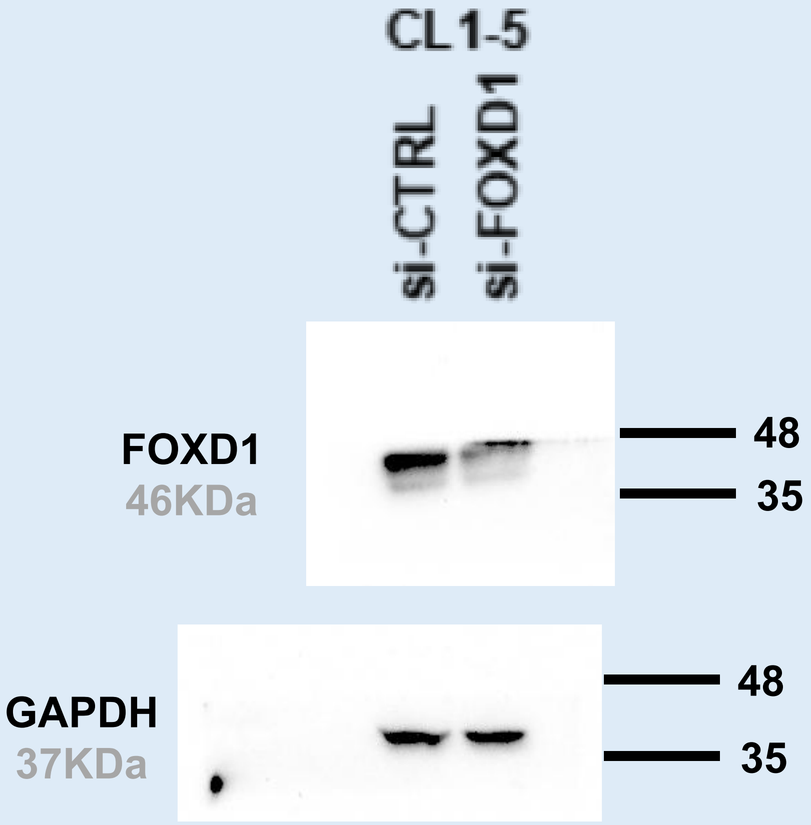

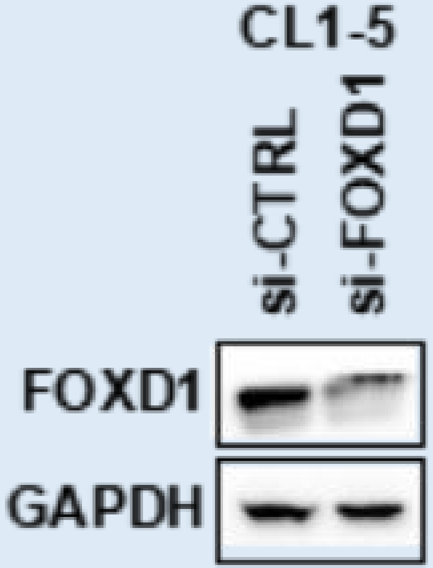

Figure S6C
RAW data

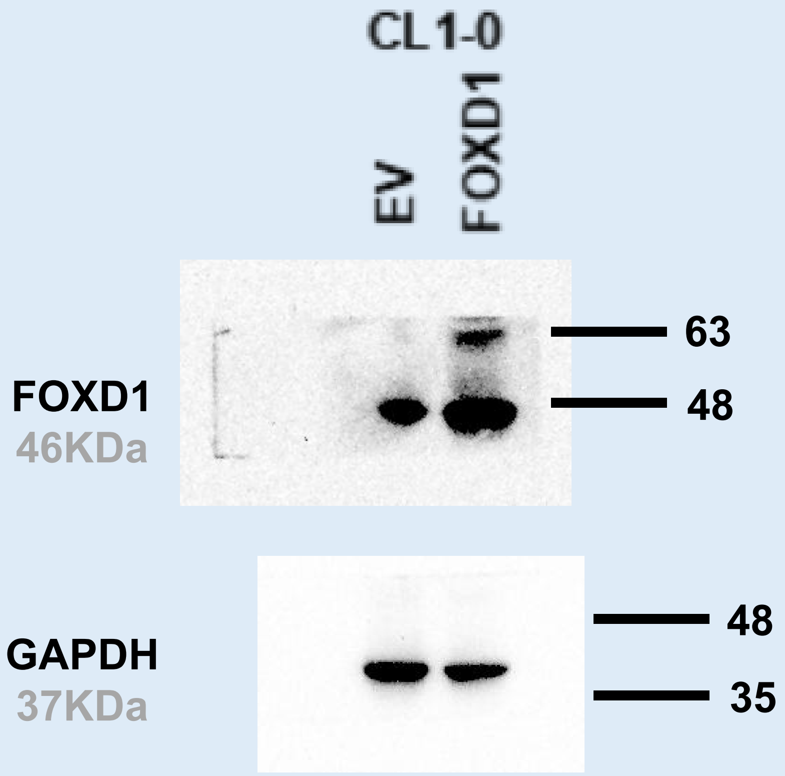

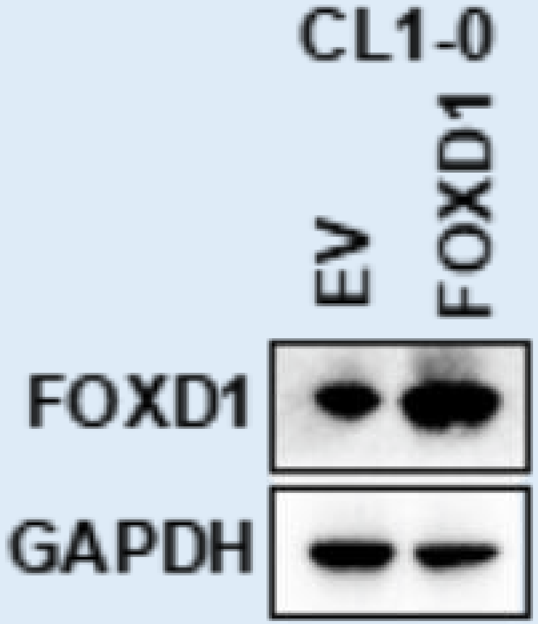

Figure S6D
RAW data

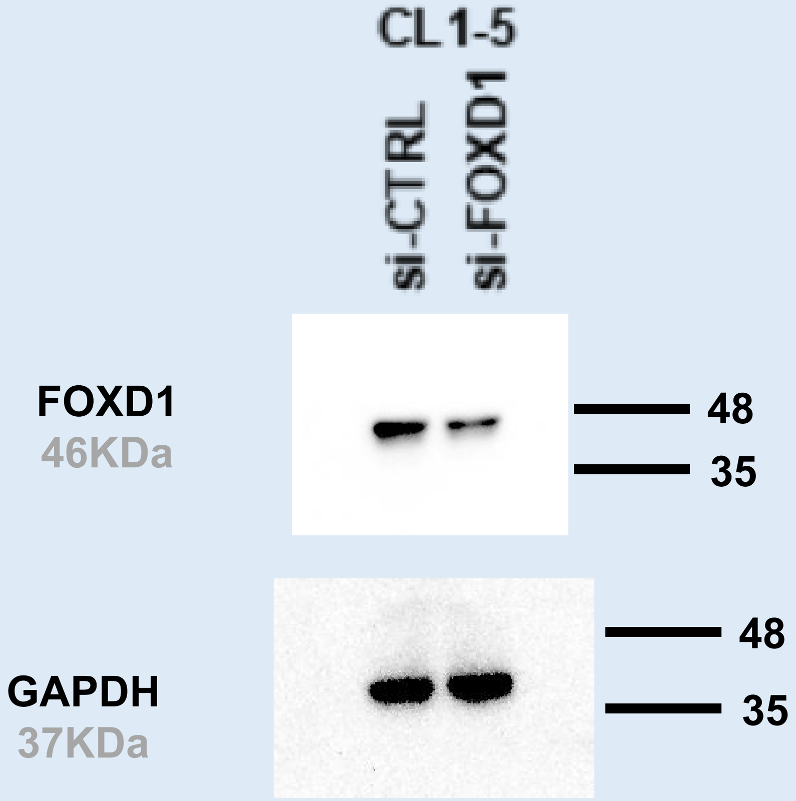

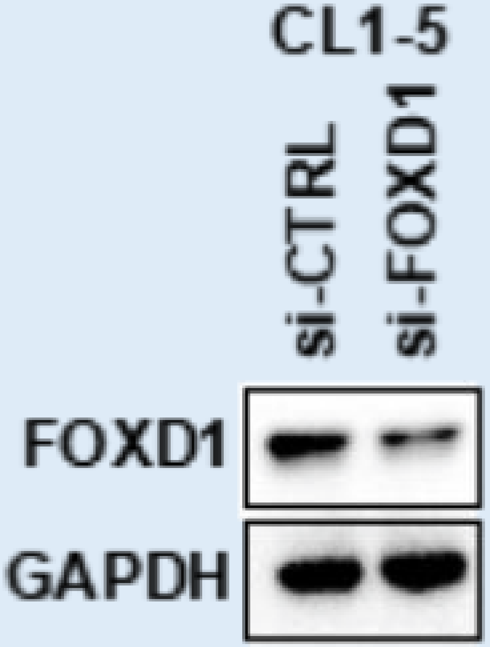

Figure S6E
RAW data

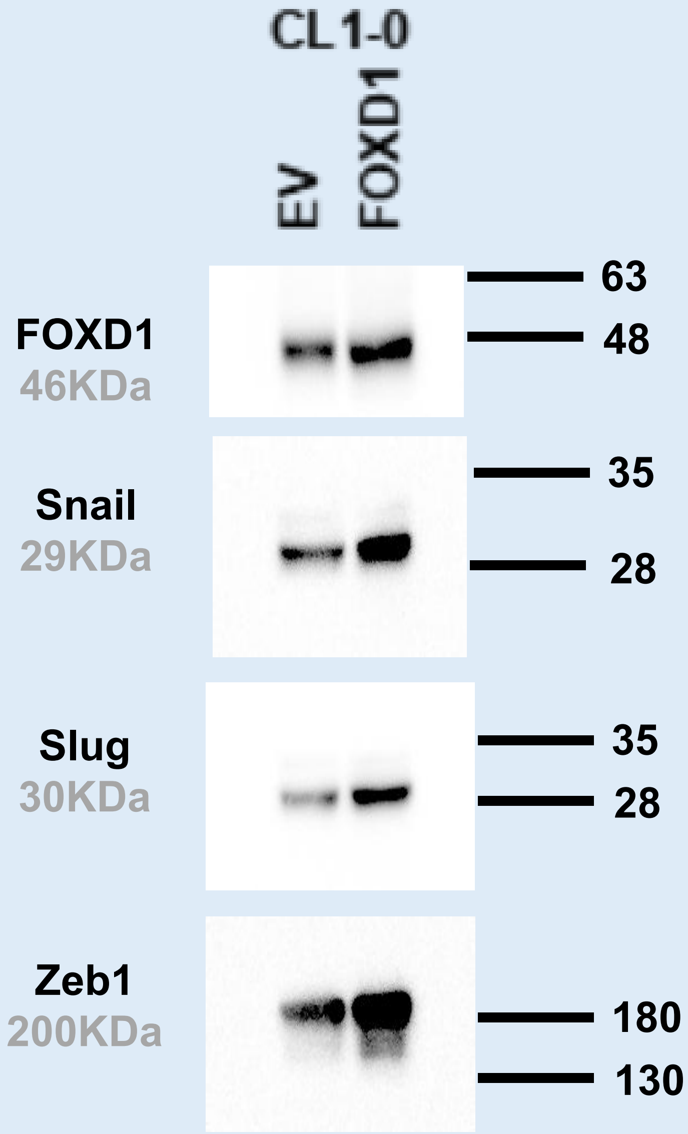

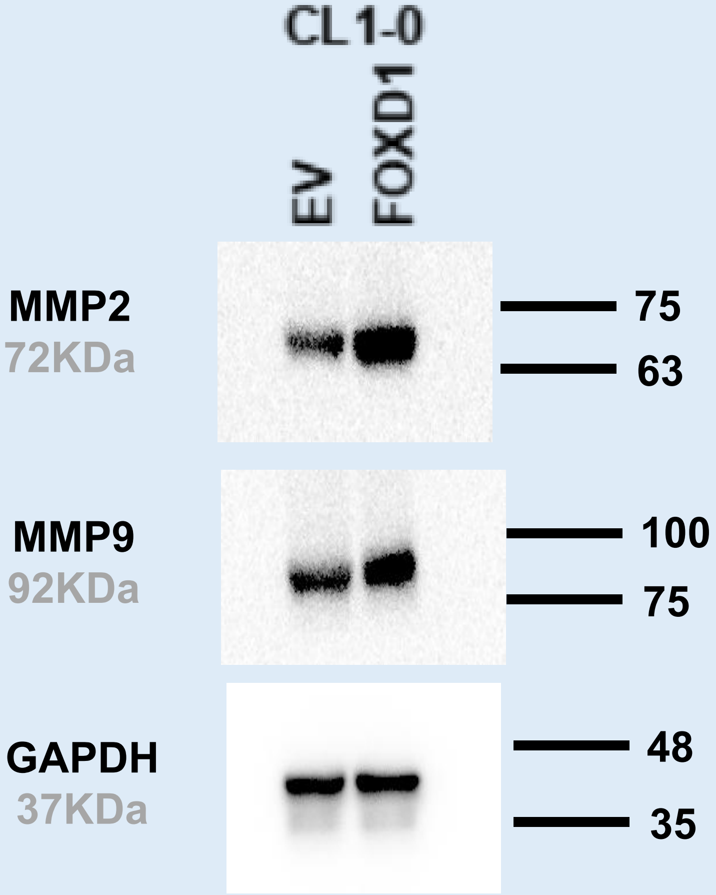

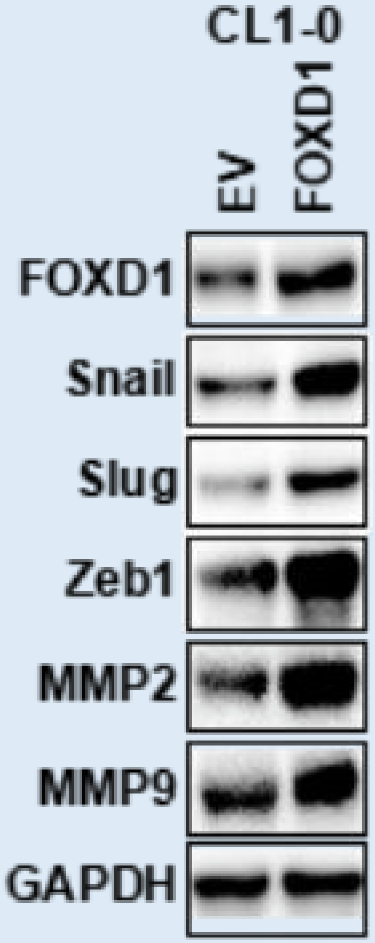

Figure S6F
RAW data

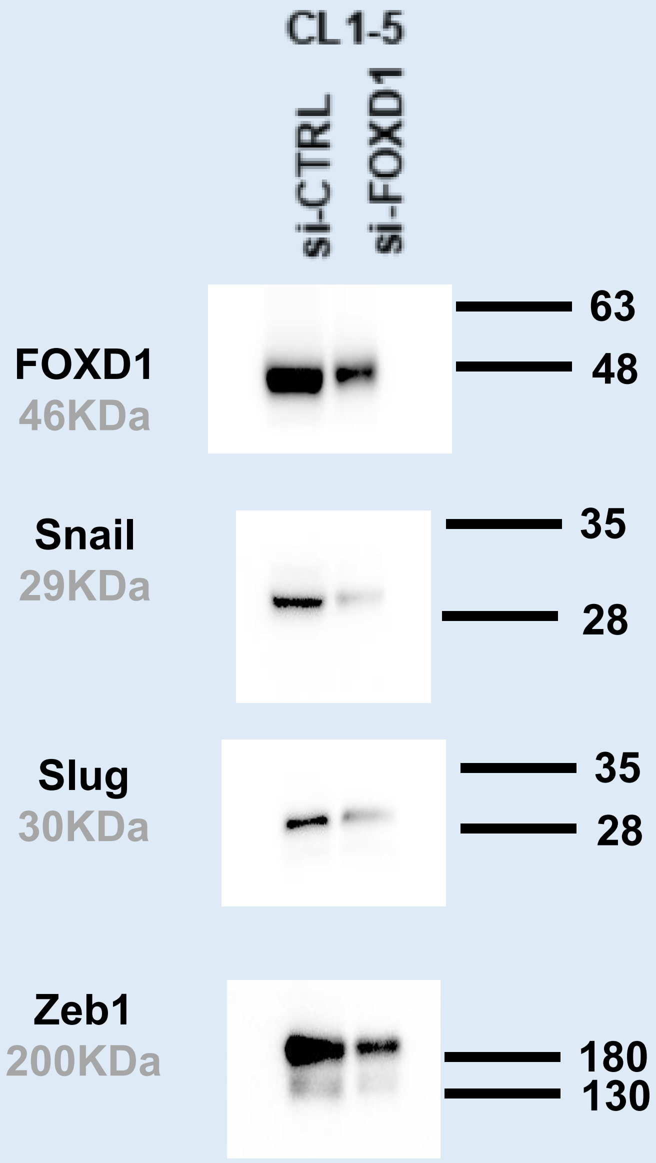

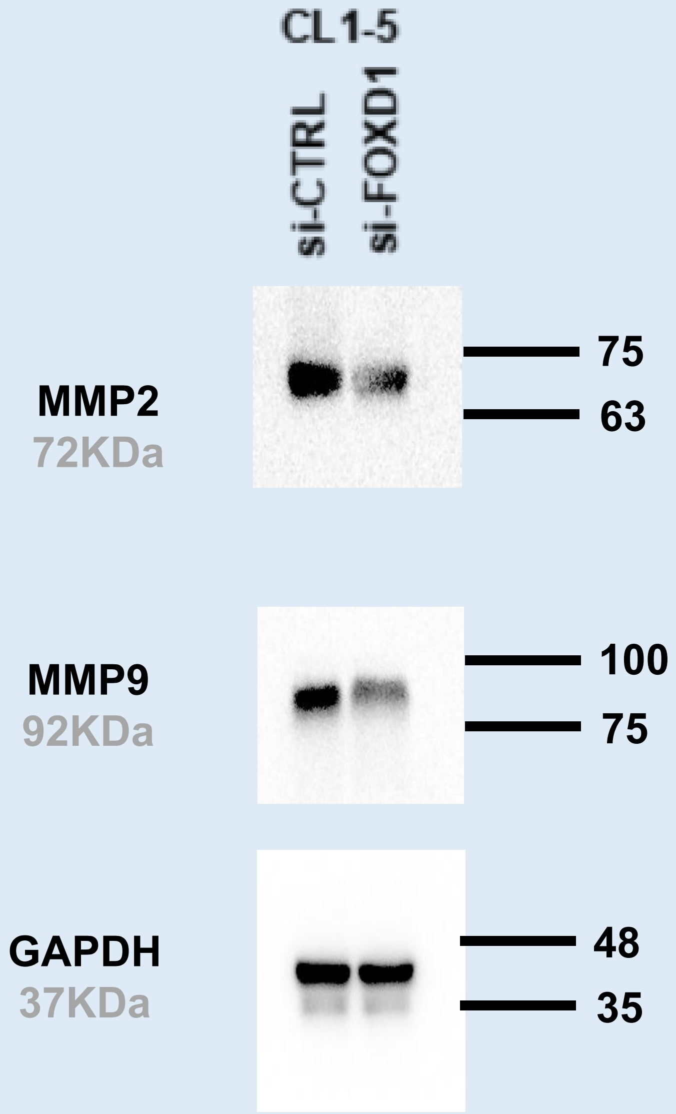

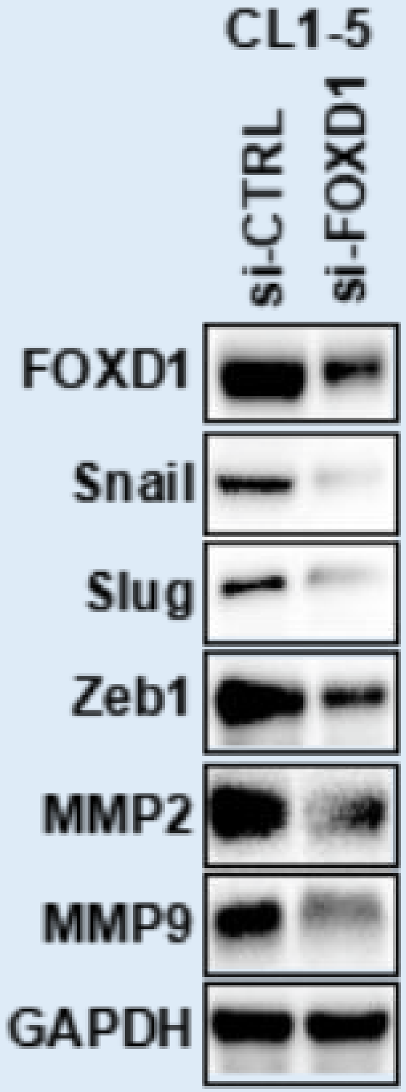

Figure S7A
RAW data

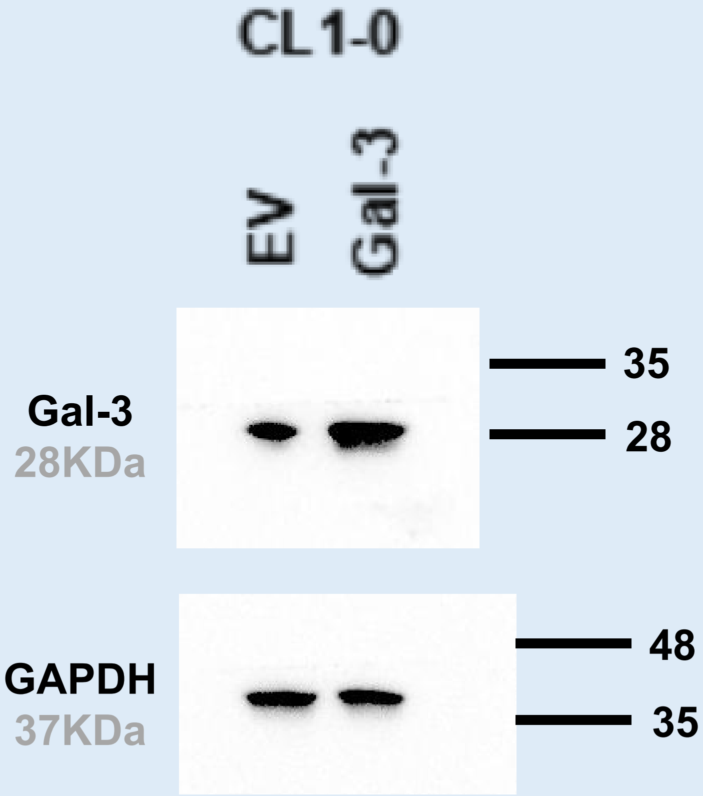

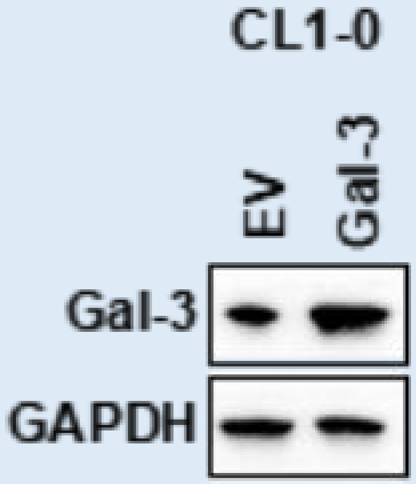

Figure S7B
RAW data

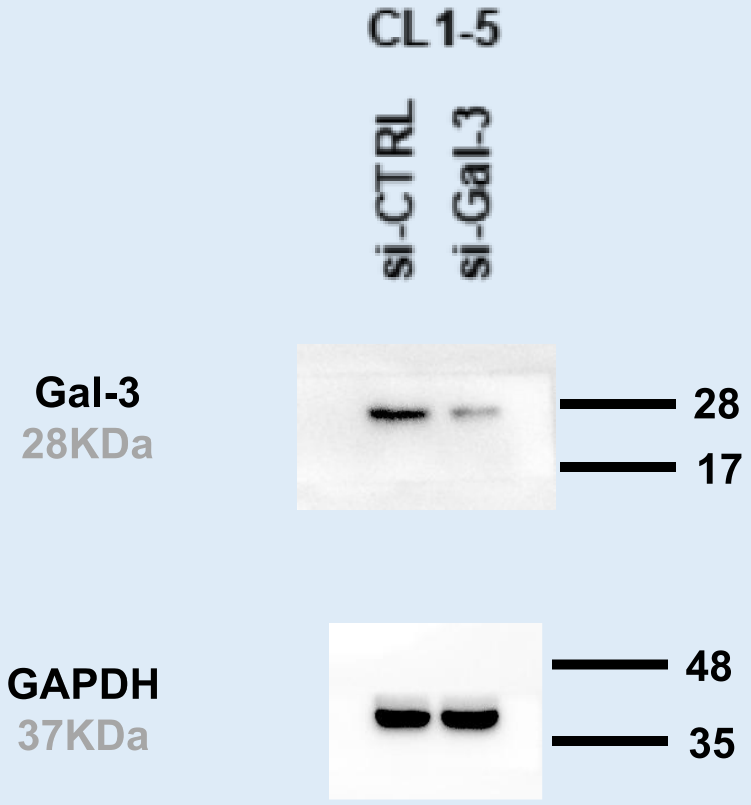

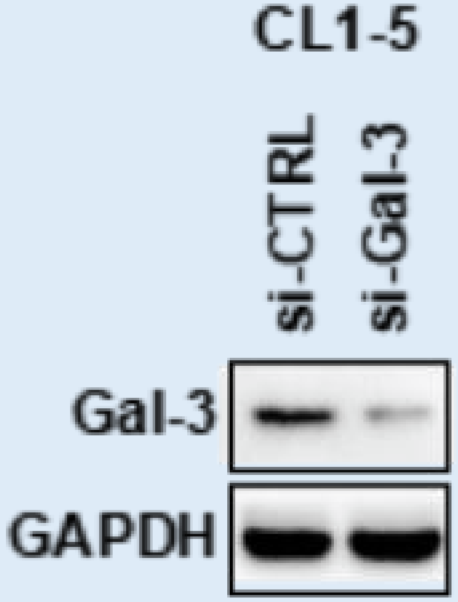

Figure S7C
RAW data

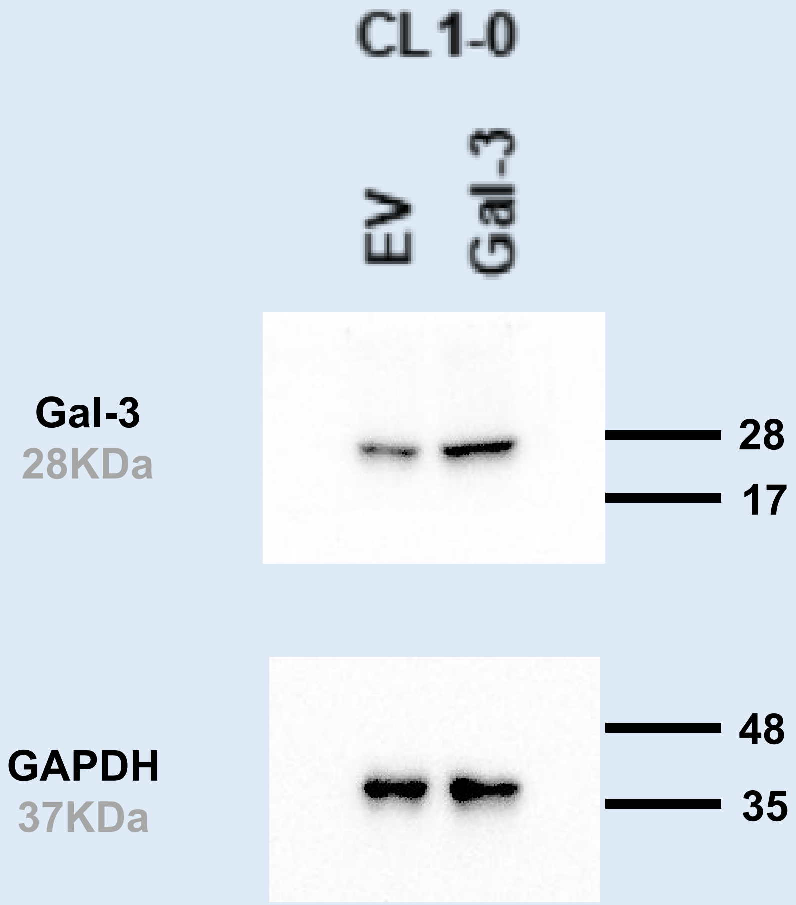

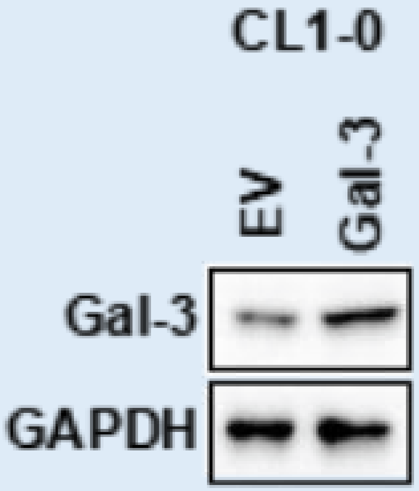

Figure S7D
RAW data

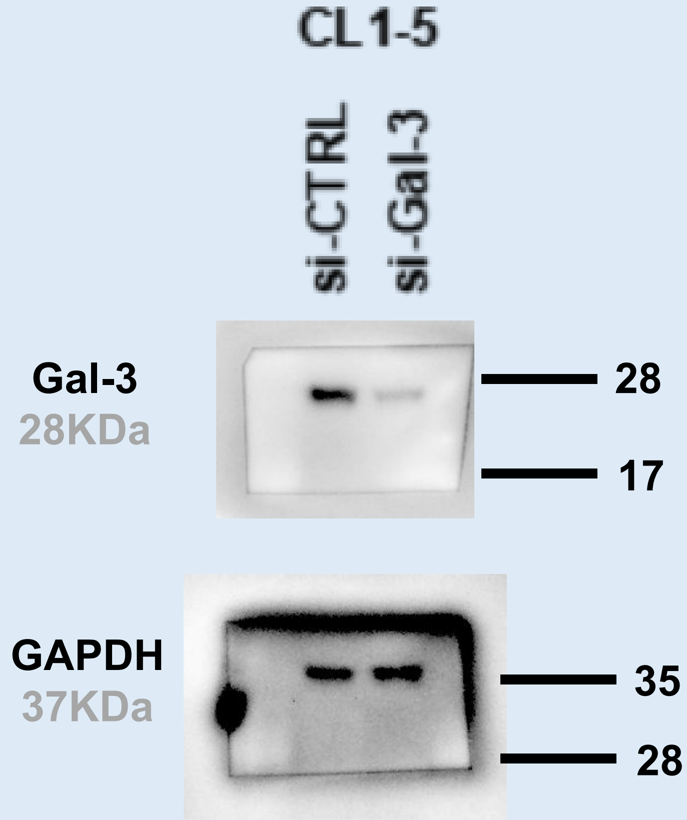

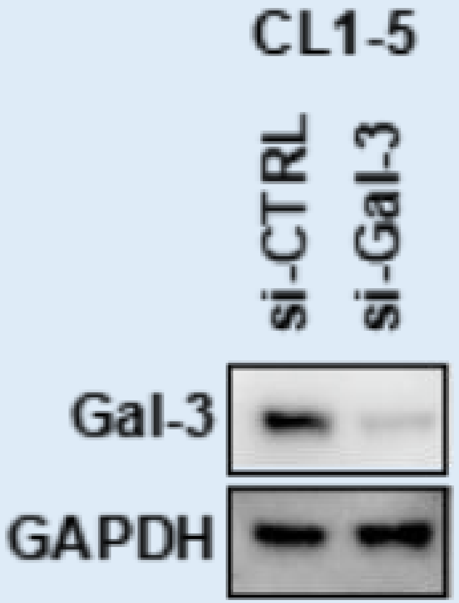

Figure S7E
RAW data

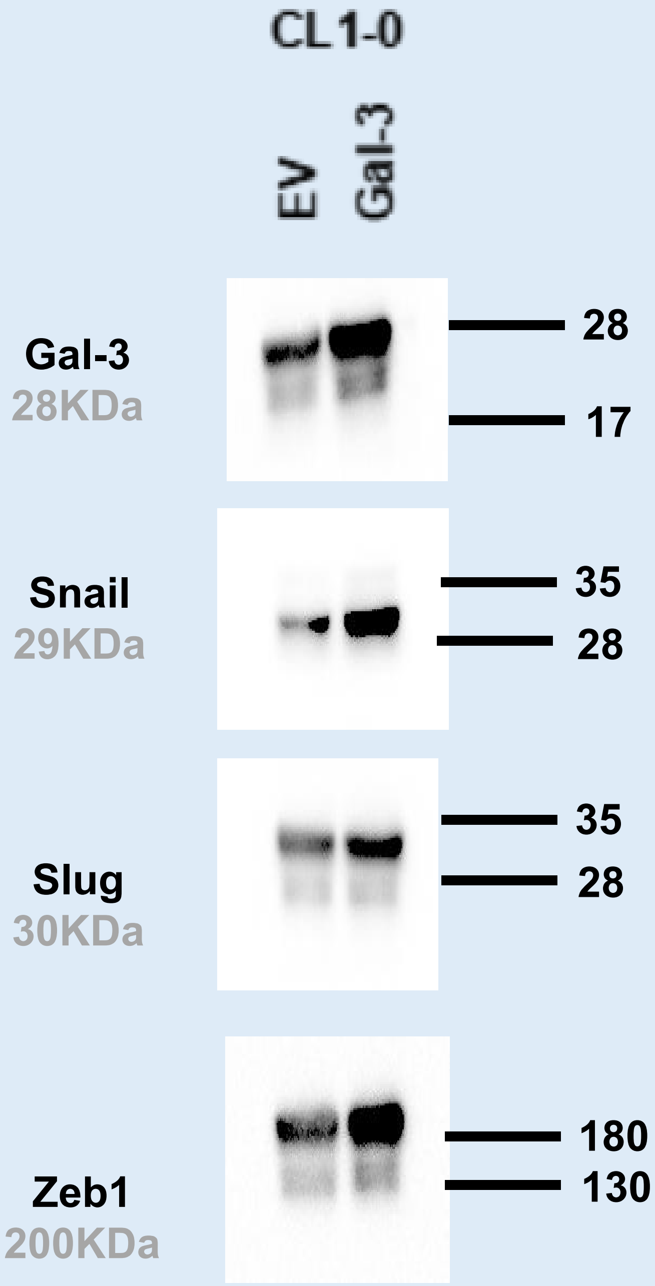

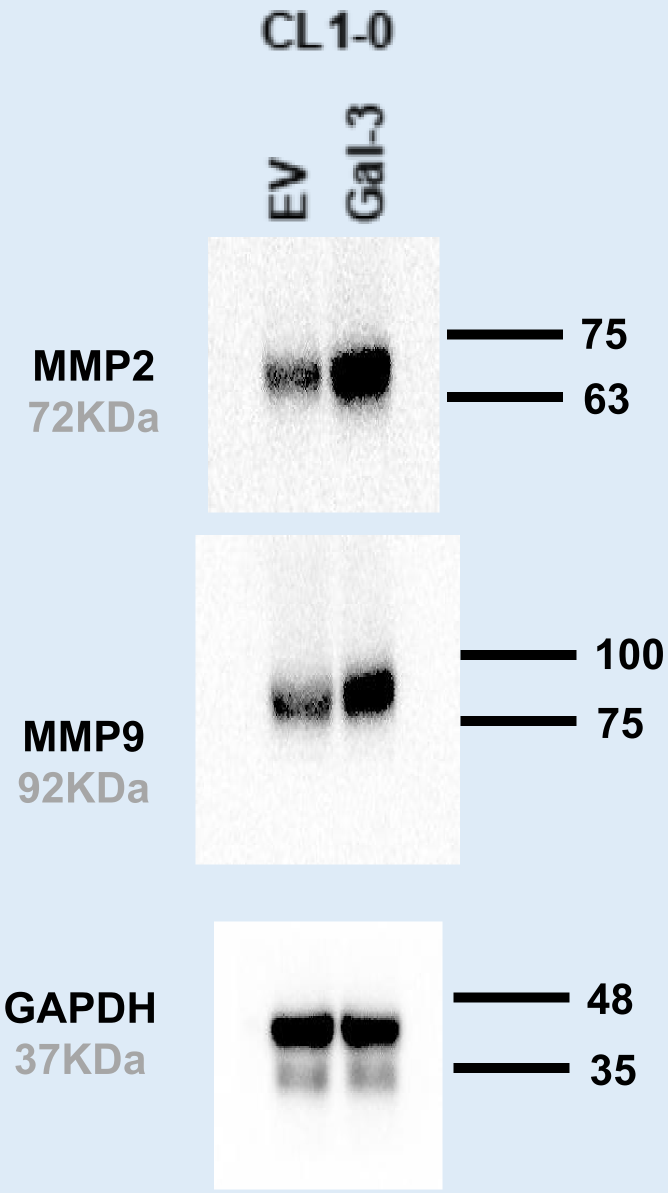

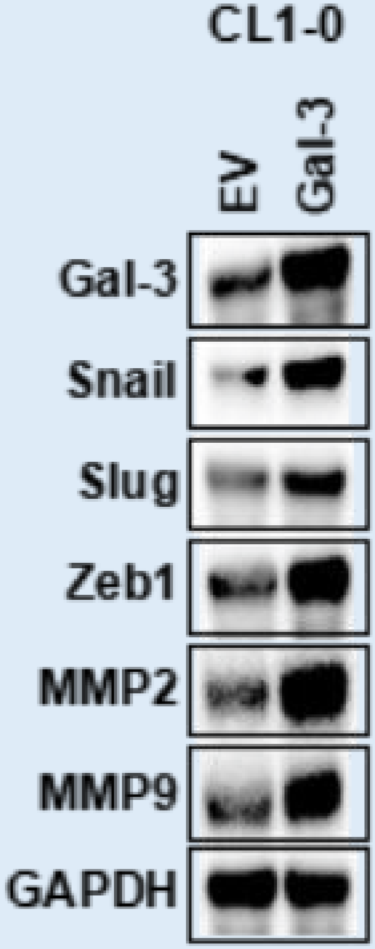

Figure S7F
RAW data

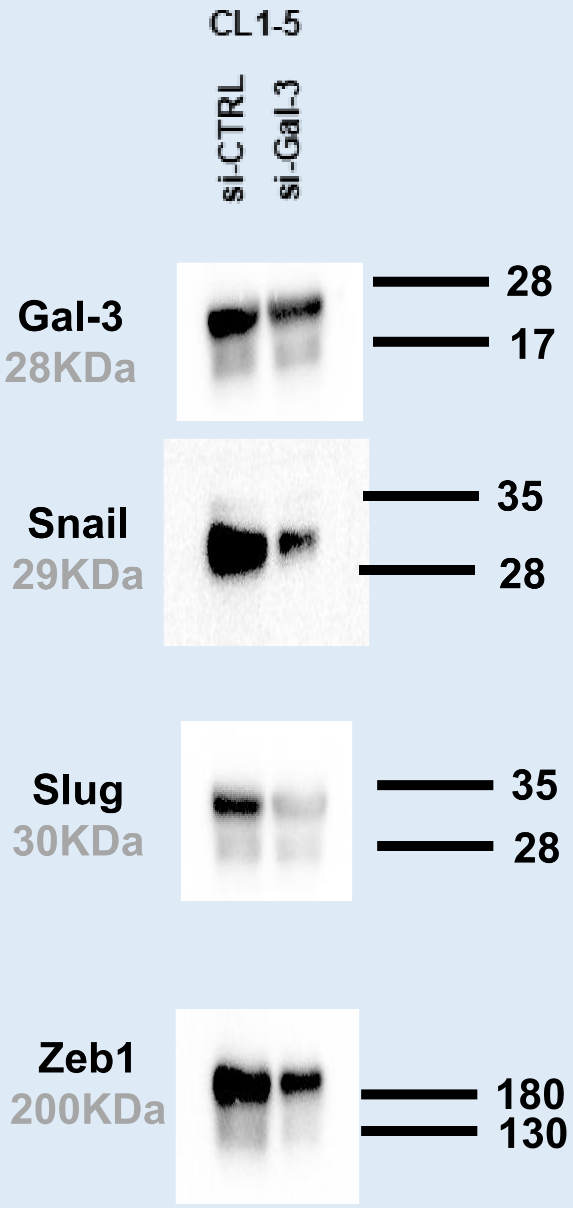

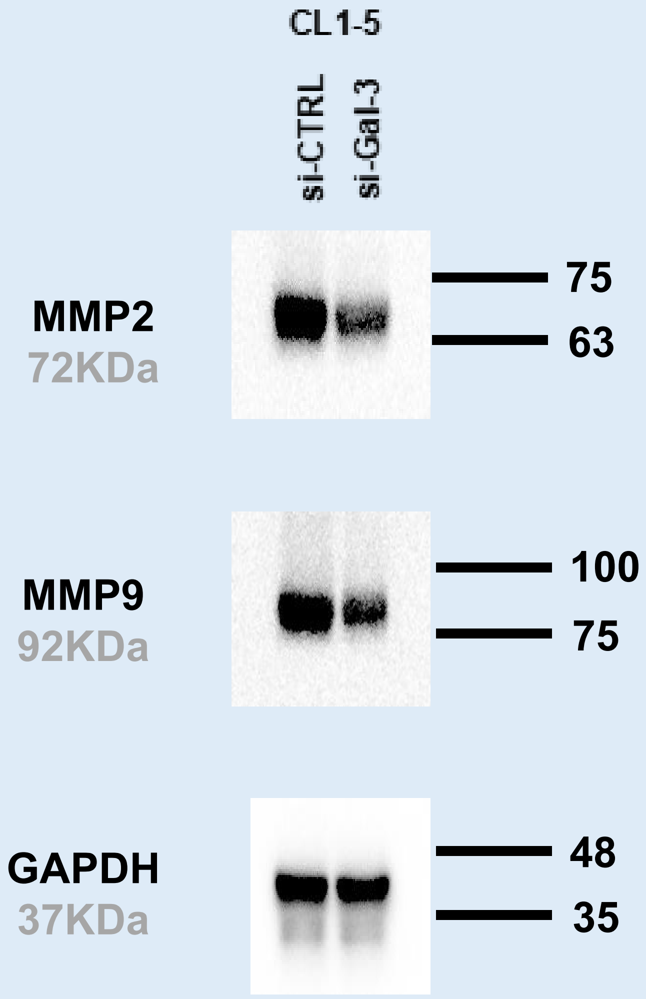

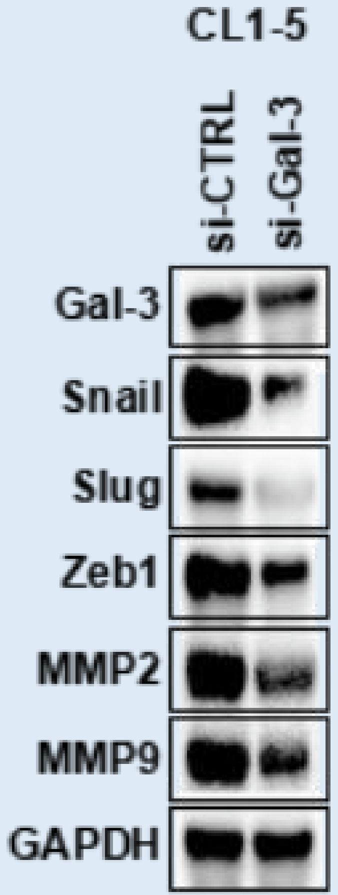

Supplement: Supplementary file 1 [file cancers-11-01897-s001.zip › cancers-644902-supplementary/Supplementary/WB Raw data.pdf]
